# Supplementary material for: Tailoring d-band center of high-valent metal-oxo species for pollutant removal via complete polymerization
Source: Nat Commun. 2024 Mar 14;15:2327. doi: 10.1038/s41467-024-46739-1 (PMC10940690; doi:10.1038/s41467-024-46739-1)
Supplement: Supplementary file 1 — Supplementary Information [file 41467_2024_46739_MOESM1_ESM.pdf]

Supplementary Information for

Tailoring *d*-band center of high-valent metal-oxo species for pollutant removal via complete polymerization

Hong-Zhi Liu[1†], Xiao-Xuan Shu[1†], Mingjie Huang[1, 2, *], Bing-Bing Wu[3], Jie-Jie Chen[1,*], Xi-Sheng Wang[3], Hui-Lin Li[1], Han-Qing Yu[1, *]

[1]CAS Key Laboratory of Urban Pollutant Conversion, Department of Environmental Science and Engineering, University of Science and Technology of China, Hefei, 230026, China

[2]School of Environmental Science and Engineering, Huazhong University of Science and Technology, Wuhan 430074, China

[3] Department of Chemistry, University of Science and Technology of China, 230026 Hefei, China

†These authors contributed equally to this work.

* Corresponding authors:

Dr. Mingjie Huang, E-mail: mingjiehuang@hust.edu.cn

Prof. Jie-Jie Chen, E-mail: chenjiej@ustc.edu.cn

Prof. Han-Qing Yu, E-mail: hqyu@ustc.edu.cn

Table of Contents

1 Supplementary Methods 3
1.1 Characterizations 3
1.2 LC-MS measurement 4
1.3 Kinetic isotope effect (KIE) experiments 5
1.4 Quantification of pollutants and oxidants 5
1.5 Total organic carbon (TOC) measurement 5
1.6 Pre-oxidation experiments 6
1.7 Collection and identification of polymerization products 6
1.8 MALDI-TOF measurement 6
1.9 C-NMR characterization 7
1.10 GPC measurement 7
1.11 Electrochemical tests 7
1.12 Computational methods 8
2 Supplementary Figures 11
3 Supplementary Tables 98
4 Supplementary Notes 101
4.1 Supplementary Note 1 | Identification of the TM (Cu, Ni, Fe)-N_4 site ... 101
4.2 Supplementary Note 2 | Confirming the critical role of high-valent metals (Cu(III)-OH, Fe(IV)=O, and Ni(IV)=O) for pollutant removal 101
4.3 Supplementary Note 3 | Polymerization removal of pollutants in TM-SA/PN-g-C_3N_4 catalytic PMS systems 104
4.4 Supplementary Note 4 | High-valent metals facilitate the oxidative polymerization of pollutants by generating phenoxyl radicals 106
4.5 Supplementary Note 5 | A note on the PT ratio 107
4.6 Supplementary Note 6 | Revelation of the over-oxidation feature in the Fe-SACs catalytic system 107

1 Supplementary Methods

1.1 Characterizations

The crystal structure and morphology of the TM-SA-g-PN-g-C_3N_4 catalysts were characterized by X-ray powder diffraction (XRD, Analytical B.V., the Netherlands) with Cu $K\alpha$ radiation (γ = 1.5418 Å) in a 2θ range of 5 to 65°. The BET-specific surface areas with N_2 adsorption/desorption isotherms were obtained from an ASAP2460 analyzer (Micromeritics Inc., Norcross, GA). The valence state and chemical composition of elements were analyzed by X-ray photoelectron spectroscopy (XPS, ESCALAB 250Xi, Thermo Fisher Scientific Inc., USA) with an Al $K\alpha$ radiation source, and all the binding energies were calibrated using the peak of C 1*s* at 284.8 eV. Scanning electron microscopy (SEM), transmission electron microscopy (TEM), and high-resolution transmission electron microscopy (HR-TEM) were performed on Phenom ProX (Phenom Co., the Netherlands), H7650 (Hitachi Co., Japan), and Talos F200X (Thermo Scientific Inc., USA) instruments, respectively. The high-angle annular dark field scanning transmission electron microscopy (HAADF-STEM) images and corresponding energy dispersive spectroscopic (EDS) mapping analyses were performed on a JEOL JEM0ARF200F TEM/STEM with a spherical aberration corrector (Talos F200X, FEI Co., USA). The functional groups on the catalyst surface were recognized by Fourier transform infrared spectroscopy (FTIR, Vertex 70, Bruker Co., Germany). The active species in the catalytic systems were identified using an electron paramagnetic resonance (EPR) spectrometer (JES-FA200, JEOL Co., Japan) (sweep time, 30.00 s; microwave power, 2.000 mW; field modulation amplitude, 1.000 G; and time constant, 20.48 ms). Raman spectra were recorded using a Horiba LabRAM HR Evolution instrument (Japan) with laser excitation at 514.5 nm at room temperature.

Soft X-ray absorption spectra (Soft-XAS, C, N K-edge, and Cu, Fe, Ni, Co L-edge) were obtained at catalysis and surface science Endstation at the BL11U beamline and Soft X-ray magnetic circular dichroism (XMCD) at the BL12B beamline in the National Synchrotron Radiation Laboratory (NSRL) in Hefei, China. X-ray absorption

fine structure (XAFS) spectroscopy measurements were performed at the Cu, Fe, Ni, and Co K-edges. Cu, Fe, Ni, and Co K-edge analyses were performed with Si (111) crystal monochromators at the BL11B beamlines at the Shanghai Synchrotron Radiation Facility (SSRF), China. Before the analysis at the beamline, samples were pressed into thin sheets 1 cm in diameter and sealed using Kapton tape films. The XAFS spectra were recorded at room temperature using a 4-channel Silicon Drift Detector (SDD) Bruker 5040. Cu, Fe, Ni, and Co K-edge extended X-ray absorption fine structure (EXAFS) spectra were recorded in transmission mode. Negligible changes in the line shape and peak position of Cu, Fe, Ni, and Co K-edge XANES spectra were observed between two scans taken for a specific sample. The XAFS spectra of these standard samples (TM-Pc and metallic oxides) were recorded in transmission mode. The spectra were processed and analyzed by the software codes Athena and Artemis. For wavelet transform analysis, the $\chi(k)$ exported from Athena was imported into the Hama Fortran code. The parameters were as follows: R range, 1 - 4 Å, k range, 0 - 10.0 $Å^{-1}$ for sample; k weight, 3; and Morlet function with $\kappa=5$, $\sigma=1$ was used as the mother wavelet to provide the overall distribution.

1.2 LC-MS measurement

The degradation intermediates and phenoxyl radical capture adducts were analyzed by using liquid chromatography-mass spectrometry (LC-MS, WatersXevoG2-XS, QTOF, Waters Co., USA) equipped with an ultra-high performance liquid chromatography (UPLC) system and an electron spray ionization source. A Waters C18 column (4.6 × 50 mm, 1.7 μm particle size) was used for UPLC separation.

For the detection of degradation products, the mobile phase was a mixture of acetonitrile : water (0.1% formic acid), the solvent ratio of the mixture was 40:60 (v:v) with a flow rate of 0.4 mL/min, and the detection wavelength was 273 nm. An ESI source in negative ionization mode was used for MS analysis. The mass calibration range was between 50 and 1000 Da and the resolution was kept above 24000.

For the identification of phenoxyl radical capture adducts, the mobile phase was a

mixture of acetonitrile: 5 mM ammonia water, the solvent ratio of the mixture was 40: 60 (v: v) with a flow rate of 0.4 mL/min, and the detection wavelength was 211 nm for CHANT and 244 nm for TEMPO. An ESI source in the positive ionization mode was used for MS analysis. The mass calibration range was between 50 and 1000 Da and the resolution was kept above 24000.

1.3 Kinetic isotope effect (KIE) experiments

Replacing H_2O with D_2O to investigate the effect of $^1O_2$. In addition, aniline was used as a model compound to investigate the formation of phenoxyl free radicals using the KIE.

1.4 Quantification of pollutants and oxidants

To quantify pollutants, ultra-high performance liquid chromatography (UHPLC, 1290 Infinity, Agilent, Inc. USA) was used with a C18 column and acetonitrile/water (0.1% formic acid) mixture as the mobile phase. For PhOH and 2,6-M-PhOH measurements, the solvent ratio of the mixture was 40:60 (v:v) with a flow rate of 0.4 mL/min, and the detection wavelength was 273 nm. For the detection of PMSO and $PMSO_2$, the wavelength was set as 215 nm.

For the quantification of oxidants (i.e., PMS or PDS), the KI spectrophotometry method[1] was used. First, a mixed solution of 10 mM KI (0.166 g KI and 0.04 g $NaHCO_3$/100mL) was prepared. Then, 0.1 mL of the filtrate was withdrawn after 0.22-μm polytetrafluoroethylene filtration at the kinetic sampling time and added to the prepared 4.9 mL KI solution. After reacting for 5 min, the mixture was analyzed at 352 nm on a UV-visible absorption spectrometer.

1.5 Total organic carbon (TOC) measurement

At given time intervals, ca. 10 mL of suspension was withdrawn from the reaction system and filtered immediately with a 0.22 μm PTFE syringe. Then, the TOC concentration of the filtrate was analyzed using a TOC analyzer (Muti N/C 2100,

Analytik Jena AG, Germany).

1.6 Pre-oxidation experiments

First, ca. 55 mg catalyst (TM-SA/PN-g-C_3N_4 or PN-g-C_3N_4) was added to a 10 mL centrifuge tube, 4 mL of 80 mM PMS (pH=2.8) was subsequently added and underwent further reaction for 30 min. Then, the solid catalysts were centrifuged and washed repeatedly with DI pure water until negligible PMS could be detected by the KI spectrophotometry method in the supernatant. Finally, the pre-oxidation catalyst was obtained by vacuum drying at 60 °C for 10 h.

For the pre-oxidation of pollutants, 50 mg of the above synthesized TM-SA/PN-g-C_3N_4 catalyst was added to a 10 mL reaction solution containing 0.5 mM pollutants under magnetic stirring. The contributions of adsorption by the TM-SA/PN-g-C_3N_4 catalyst and oxidation by the PN-g-C_3N_4 substrate were also studied to reveal the direct oxidative degradation of pollutants by high-valent metal species.

1.7 Collection and identification of polymerization products

To collect the polymerization products on the catalyst surface, the reaction system was amplified five times with the reaction conditions of [Cat] = 1.0 g/L, [PMS] = 1.0 mM, and [2, 6-M-PhOH] = 0.5 mM. After the reaction, the catalyst was repeatedly washed with THF to obtain a yellow solution. Then, the solvent was dried in an oven at 80 °C to obtain a soft brown solid for further characterizations.

1.8 MALDI-TOF measurement

The above soft brown polymer solids were re-dissolved in THF and characterized by matrix-assisted laser desorption/ionization time-of-flight mass spectrometry (MALDI-TOF MS) (Atouflex Speed, Bruker Inc., USA).

1.9 C-NMR characterization

The above soft brown polymer solids were dissolved in $CDCl_3$, and their structure was identified by nuclear magnetic resonance (NMR) spectroscopy in a NMR spectrometer (AVANCE III 500 MHz/54 mm, Bruker Inc., USA).

1.10 GPC measurement

The above soft brown polymer solids were re-dissolved in THF, and the molecular weight and polymer dispersity index (PDI) were measured using an 150C gel permeation chromatography (GPC, Waters Inc., USA) equipped with Microstyragel columns and an RI 2414 detector at 35 ºC. The flow rate of the THF eluent was controlled at 1.0 mL/min. The final results were calibrated using monodispersed polystyrene standards for molecular weight identification.

1.11 Electrochemical tests

Preparation of catalyst-coated glassy carbon electrode (catalyst-GCE)

Catalyst ink was prepared by adding 10 mg catalyst (TM-SA/PN-g-C_3N_4) into a mixed solution containing 0.05 mL Nafion perfluorinated resin solution and 0.75 mL ethanol. The mixture was sonicated for 30 min to evenly disperse the catalyst, and then 6 μL of the mixture was dripped onto the surface of a glassy carbon electrode (GCE, Φ3 mm) and dried at 60 ºC.

Linear sweep voltammetry (LSV) analysis

LSV analyses were performed between 0.0 V and 1.0 V at a scan rate of 20 mV s^{-1} on a CHI 760E electrochemical workstation. The experiments were conducted in 0.1 M Na_2SO_4 solution and 2.5 mM pollutant (PhOH) at pH = 6.9 with a three-electrode cell configuration including a working electrode (catalyst-GCE), a counter electrode (platinum electrode, 10 × 10 × 0.1 mm), and a reference electrode (Ag/AgCl electrode). All the potential values were normalized to the reversible hydrogen electrode (RHE)

before testing (eq. S1).

$$E_{vs.RHE} = E_{vs.Ag/AgCl} + 0.197 + 0.0591 \times pH \qquad (S1)$$

Cyclic voltammetry (CV) analysis

CV was used to determine the oxygen reduction potential of the high-valence metal species. Unlike the LSV tests, no PhOH was added to the CV measurement systems. The voltage setting ranges of Cu, Fe, Ni, and Co-SA/PN-g-C_3N_4 were -1.0~1.5, -0.5~2.2, -0.4~2.0, and 0.6 to 2.0 V_{RHE}, respectively.

Chronoamperometry tests

Amperometric i-t curves were obtained under the same conditions as the voltammetry tests, with the working electrode biased to the applied open circuit voltage of the corresponding system (vs Ag/AgCl). PMS or phenol was added to the solution to monitor the changed current.

Open circuit potential measurements

Using a Ag/AgCl electrode as a reference electrode, the open circuit potential of the catalyst-GCE was detected by chronopotentiometry. All potential values were normalized to a reversible hydrogen electrode (RHE). The sampling time was set to 1200 s, PMS was added at 100 s, and PhOH was added at 600 s.

Electrochemical impedance spectroscopy (EIS) measurements

The EIS measurements were carried out using a similar three-electrode system at a potential bias applied to 10 mV (V_{RHE}) and a scanning frequency in the range of 0.1~100 kHz.

1.12 Computational methods

All density functional theory (DFT) calculations were performed using CASTEP

code[2]. To describe the interaction of electron-ion, PBE functional with spin polarization of the generalized gradient approximation approach (GGA-PBE) was adopted for the exchange-correlation energy[3]. van der Waals (VDW) forces were corrected with Grimme's DFT-D method[4] to depict highly accurate noncovalent forces, and the Monkhorst-Pack mesh with a 3 × 3 × 1 grid was sampled for all surface calculations. An OFTG ultrasoft pseudopotential was used with the plane wave cutoff set to 500 eV and a smearing width of 0.1 eV throughout all geometry optimization and property calculations. Moreover, geometry optimization was performed until the maximum displacement and the maximum force were within 1×10^{-3} Å and less than 0.03 eV/Å, respectively. The minimum energies were obtained until the energy was less than 1×10^{-5} eV per atom. The phonon calculations were performed by the linear response method with a norm-conserving pseudopotential.

To construct the model of the catalyst, we built a supercell (6 × 7) pyrrolic N-rich g-C_3N_4 (PN-g-C_3N_4) substrate including 16 C atoms, 19 N atoms, and 7 H atoms. The transition metal single-atom sites (SA-TM, TM = Cu, Ni, Co, Fe) were coordinated by two pyridinic N and two pyrrolic N atoms[5]. The vacuum layer was set to 20 Å along the z direction to prevent the slabs from interacting.

We first tested different adsorption sites for PMS to find the most optimized adsorption configuration. The adsorption energy (ΔE_{ads}) of PMS on the TM-SA/PN-g-C_3N_4 surface is obtained by the following formula:

$$E_{ads} = E_{*PMS} - E_{PMS} - E_{*} \tag{S2}$$

where E_{*PMS}, E_{PMS}, and E_{*} represent the energies of the surface-adsorbate, free adsorbate, and bare substrate, respectively.

Moreover, to investigate the PMS activation activity, the projected density of states (PDOS), population analysis, and difference charge density were analyzed based on the surface models. To probe the electron interaction between the adsorbate and SA/PN-g-C_3N_4 before and after PMS adsorption, the TM 3*d* band centers were determined by taking the weighted mean energy of their PDOS. Mulliken charge analysis was performed to investigate the charge transfer. The difference in charge density during

PMS adsorption is defined by the following formula:

$$d\rho = \rho_{*PMS} - \rho_{*} - \rho_{PMS} \tag{S3}$$

where $\rho_{*\mathrm{PMS}}$, ρ_{*}, and ρ_{PMS} denote the charge density of the whole system, the sole *, and the frozen PMS, respectively.

The Gibbs free energy for PMS activation and the 2, 6-M-PhOH degradation reaction is defined as follows:

$$\Delta G = \Delta E_{DFT} + \Delta ZPE + T\Delta S \tag{S4}$$

where ΔE_{DFT}, ΔZPE, ΔS, and T are the final enthalpy from DFT calculations, zero-point energy, entropic contributions, and temperature (298.15 K, in our work), respectively. Due to the minimal contribution of the catalyst surface vibrations caused by the adsorbates[6], ΔZPE and ΔS were obtained from isolated adsorbates in our work.

2 Supplementary Figures

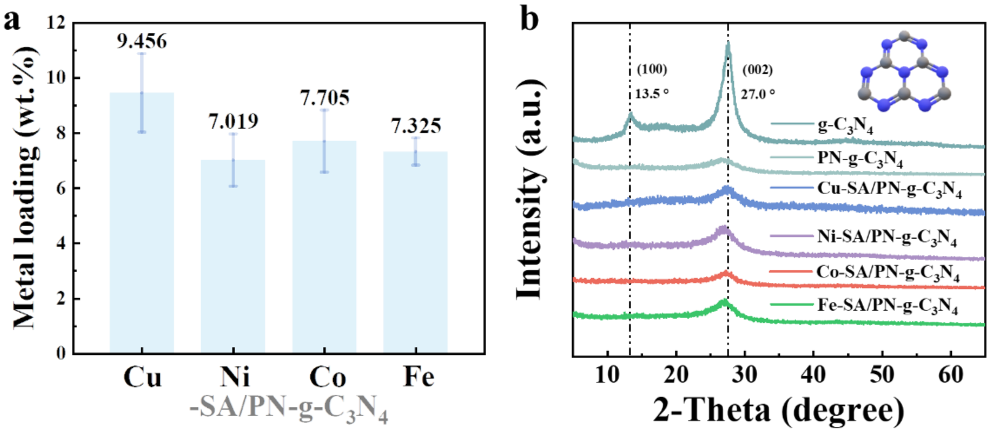

Supplementary Fig. 1 | Metal load and material base properties. a, The metal loading and **b**, XRD patterns of TM (Cu, Ni, Co, Fe)-SA/PN-g-C_3N_4. In the inset of Fig. 1b, the blue atom represents N and the gray atom represents C. Error bars represent the standard deviation, obtained by repeating the experiment two times.

The two peaks at 13.5° and 27.0° could be attributed to intralayer distances between tri-s-triazine rings and PN-C_3N_4 layers, respectively[7].

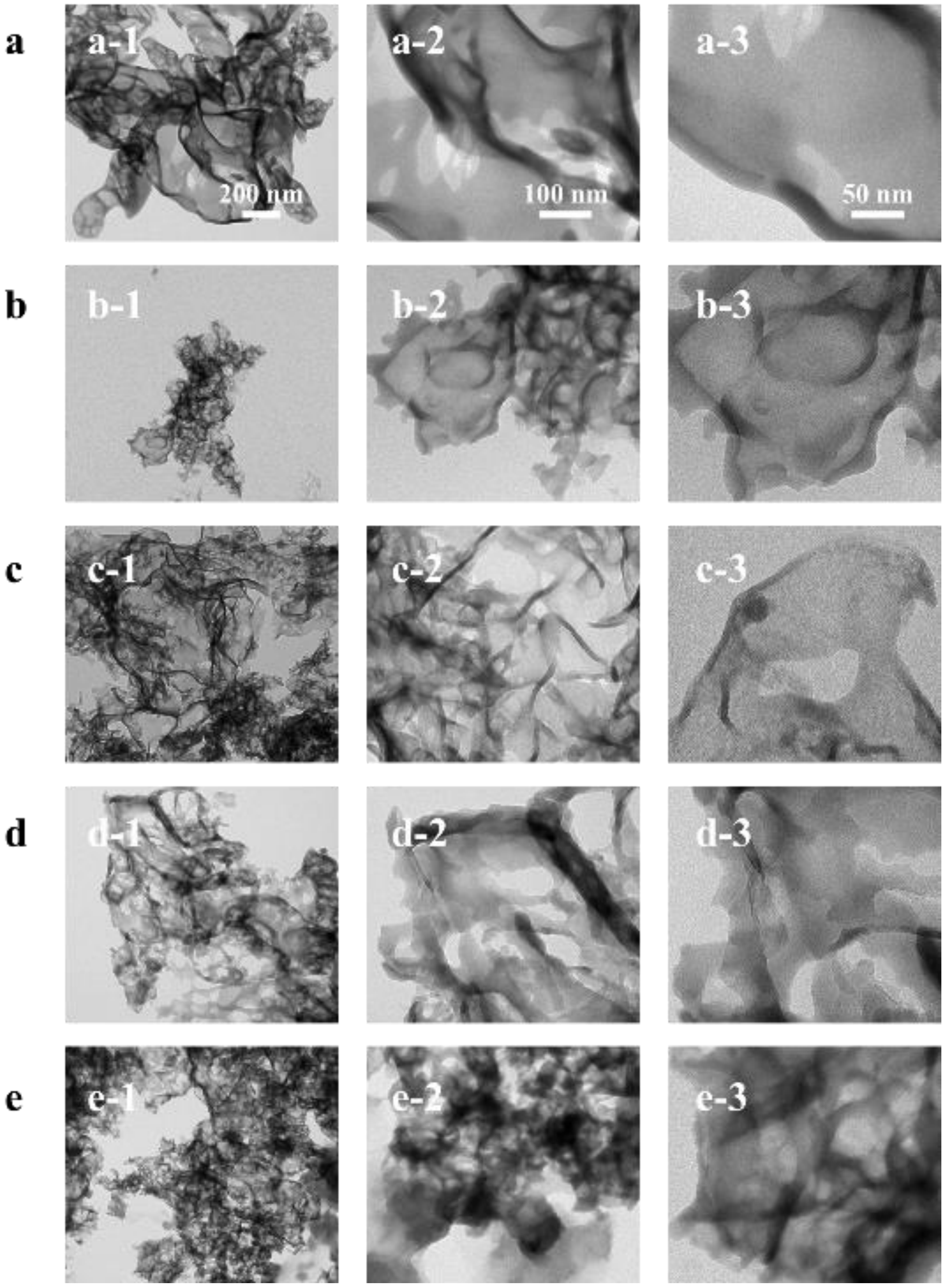

Supplementary Fig. 2 | HR-TEM morphology of materials. a-e, HR-TEM images of (**a**) PN-g-C_3N_4, **(b)** Cu-SA/PN-g-C_3N_4, (**c**) Ni-SA/PN-g-C_3N_4, (**d**) Co-SA/PN-g-C_3N_4, and **(e)** Fe- SA/PN-g-C_3N_4.

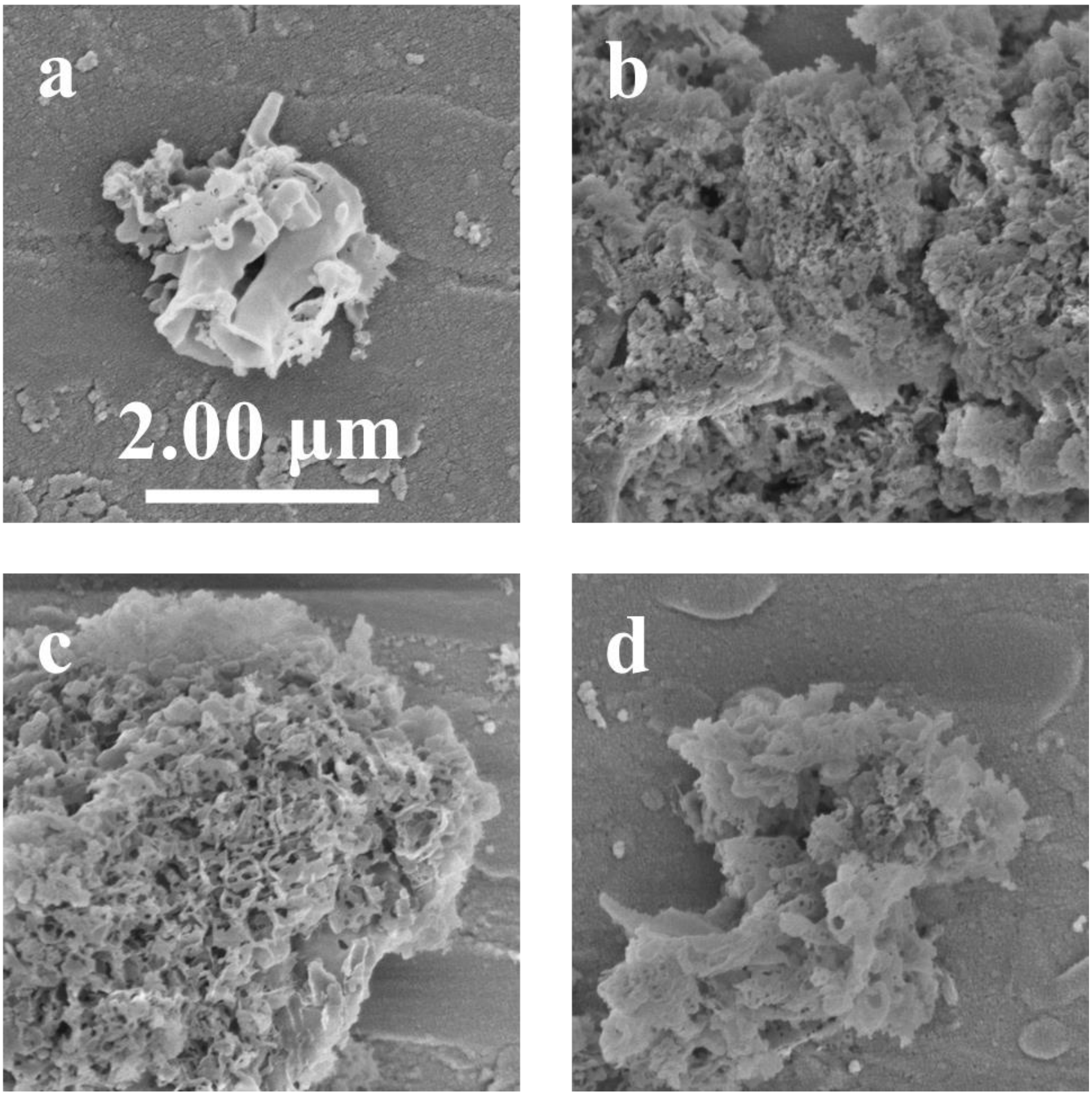

Supplementary Fig. 3 | SEM morphology of materials. a-d, SEM images of **(a)** PN-g-C_3N_4, **(b)** Cu-SA/PN-g-C_3N_4, **(c)** Ni-SA/PN-g-C_3N_4, and **(d)** Fe-SA/PN-g-C_3N_4, respectively. The scale bar in **a** applies to all other figures.

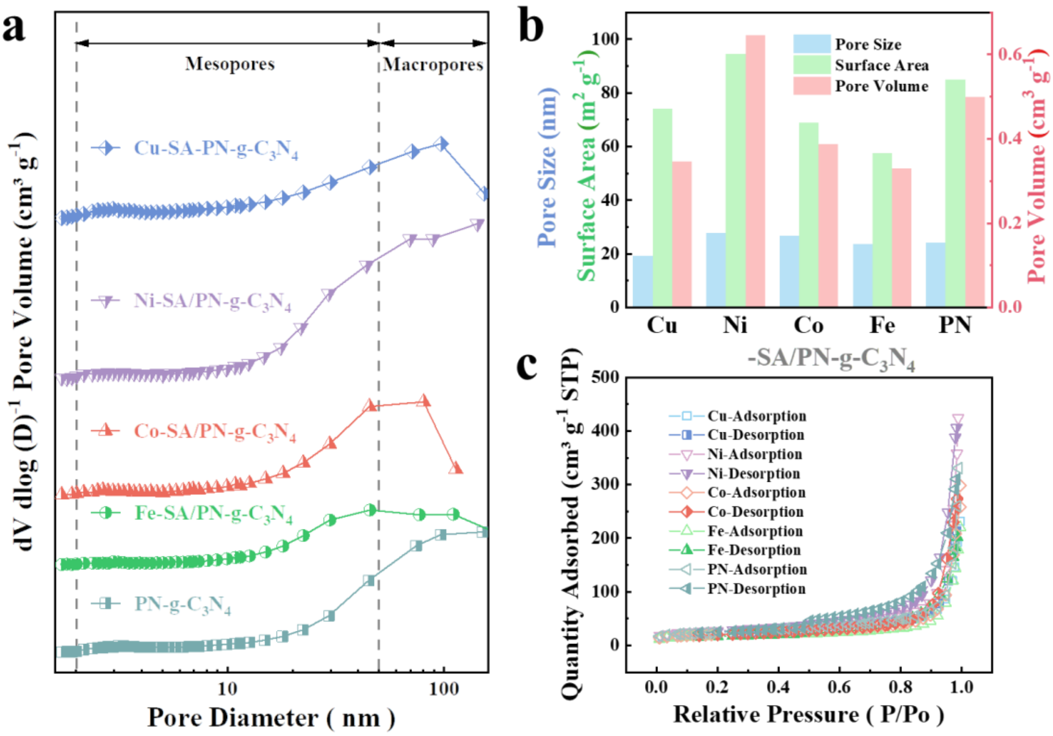

Supplementary Fig. 4 | BET characterization result. a-c, Pore size distribution of TM-SA/PN-g-C_3N_4 **(a).** Pore size, surface area, and pore volume of TM-SA/PN-g-C_3N_4 **(b)**. N_2 adsorption and desorption isotherms of TM-SA/PN-g-C_3N_4 **(c).**

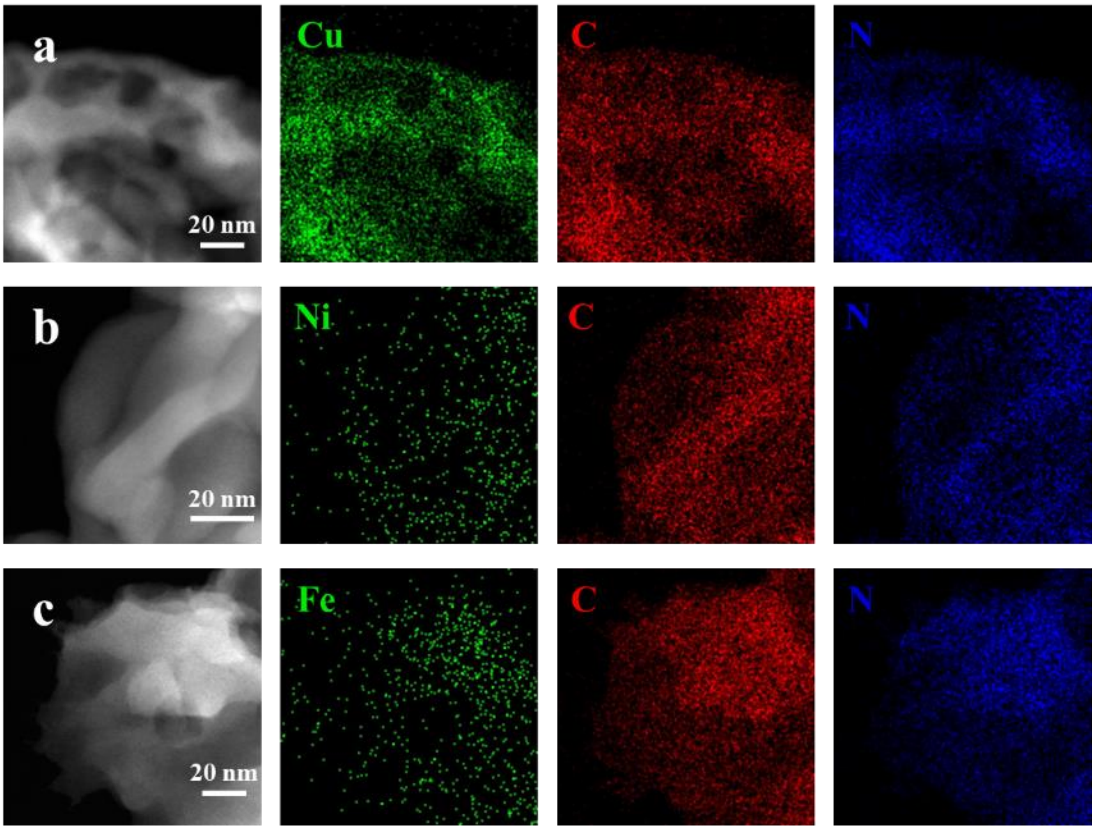

Supplementary Fig. 5 | Element distribution of materials. a-c, HAADF-STEM images and corresponding EDS elemental mappings of **(a)** Cu-SA/PN-g-C_3N_4, **(b)** Ni-SA/PN-g-C_3N_4, and **(c)** Fe-SA/PN-g-C_3N_4.

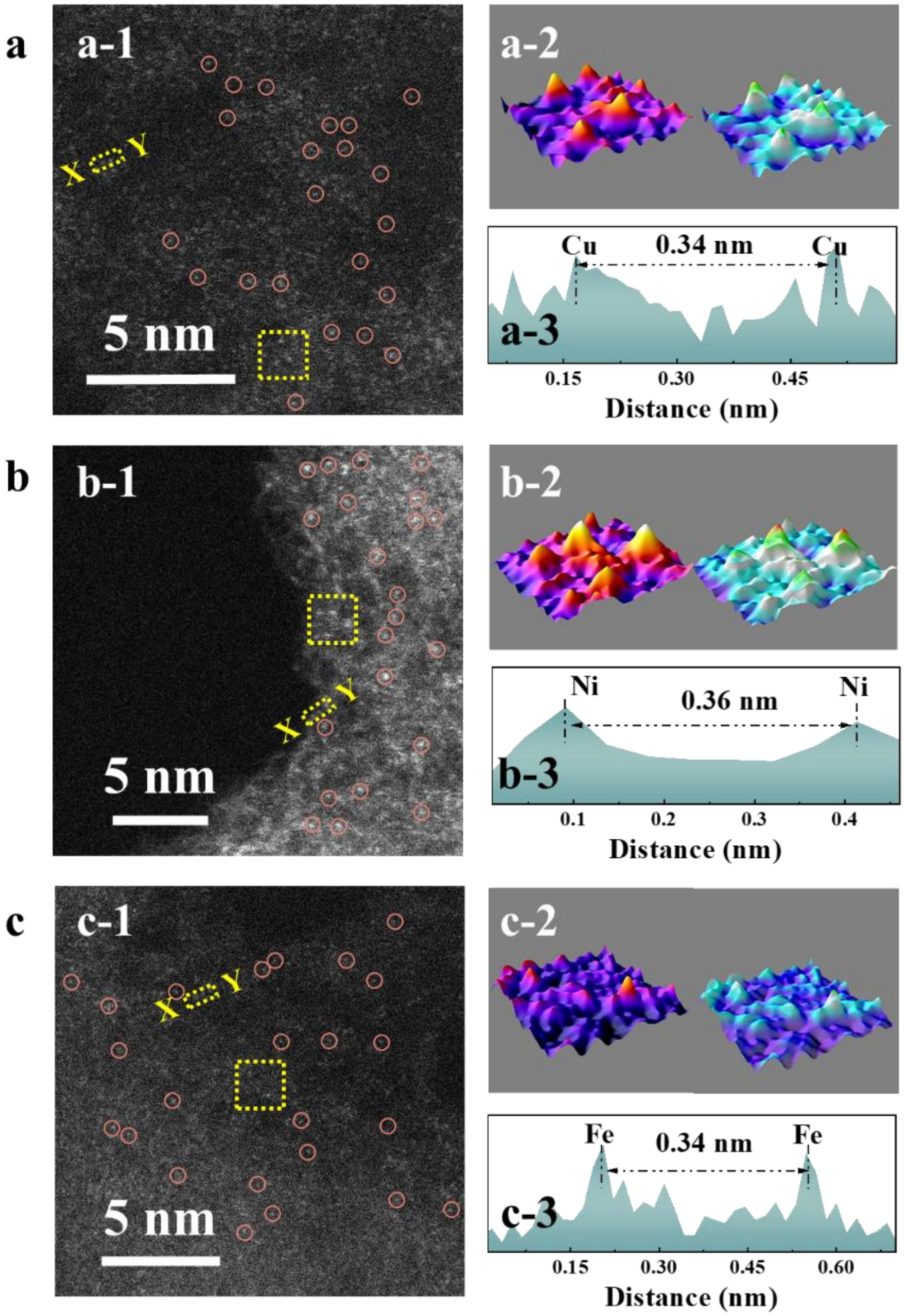

Supplementary Fig. 6 | Electron microscopic characterization of single atom. a-c, AC HAADF-STEM images of **(a)** Cu-SA/PN-g-C_3N_4, **(b)** Ni-SA/PN-g-C_3N_4, and (**c**) Fe-SA/PN-g-C_3N_4. 1, 2, 3 represent corresponding 3D isolines and atom-overlapping Gaussian-function fitting mapping of the yellow rectangle from **a-1**, **b-c**, and **c-1** and intensity profile along *X*–*Y* in **a-1**, **b-c**, and **c-1**. The single atoms are marked with isolated red circles.

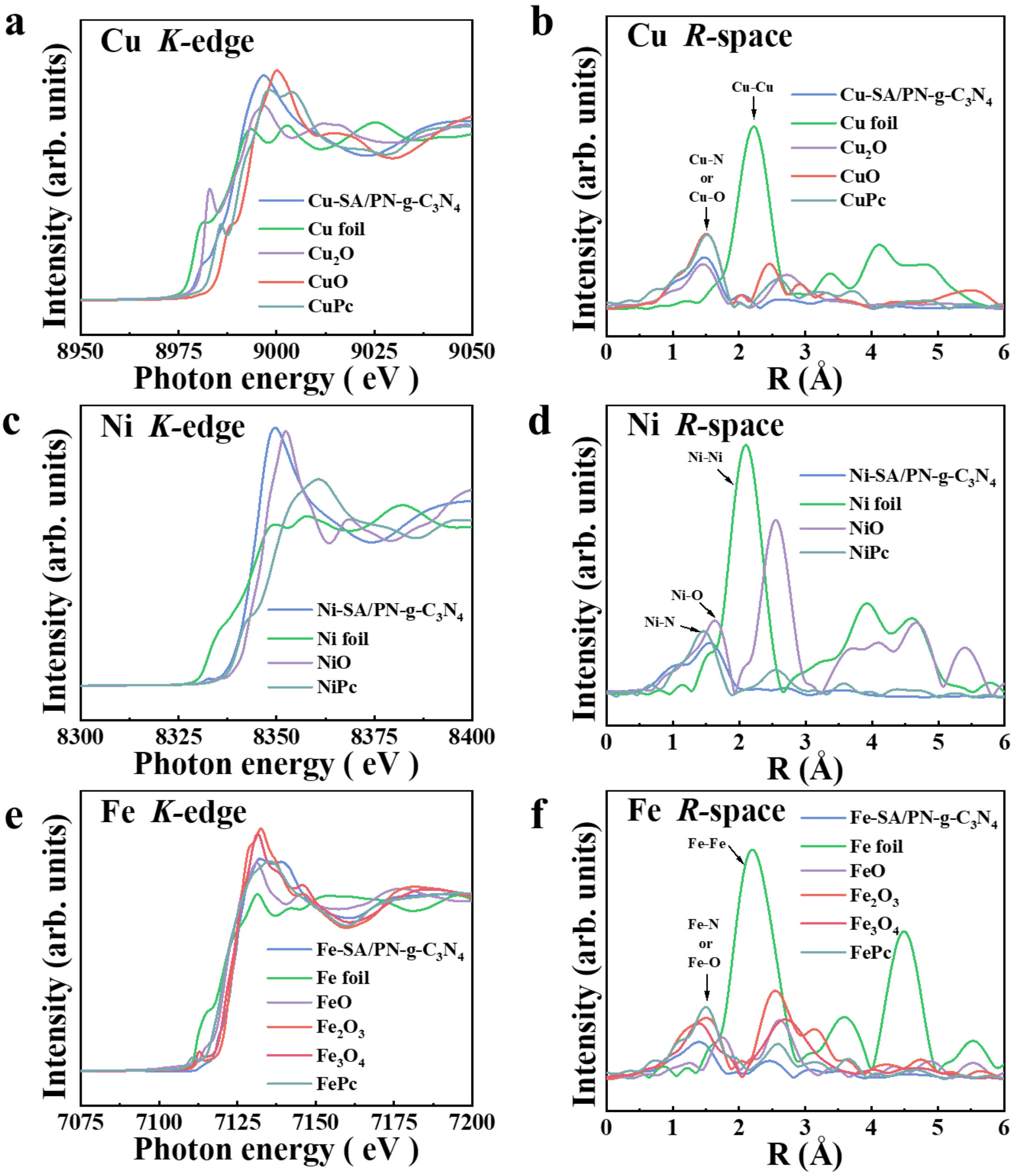

Supplementary Fig. 7 | XANES analysis a, c, e, Cu *K*-edge **(a)**, Ni *K*-edge **(c)**, and Fe *K*-edge **(e)** XANES spectra of TM (Cu, Ni, Fe)-SA/PN-g-C_3N_4. **b, d, f,** Fourier-transformed Cu *R*-space **(b)**, Ni *R*-space, and Fe *R*-space **(f)** EXAFS spectra of TM (Cu, Ni, Fe)-SA/PN-g-C_3N_4.

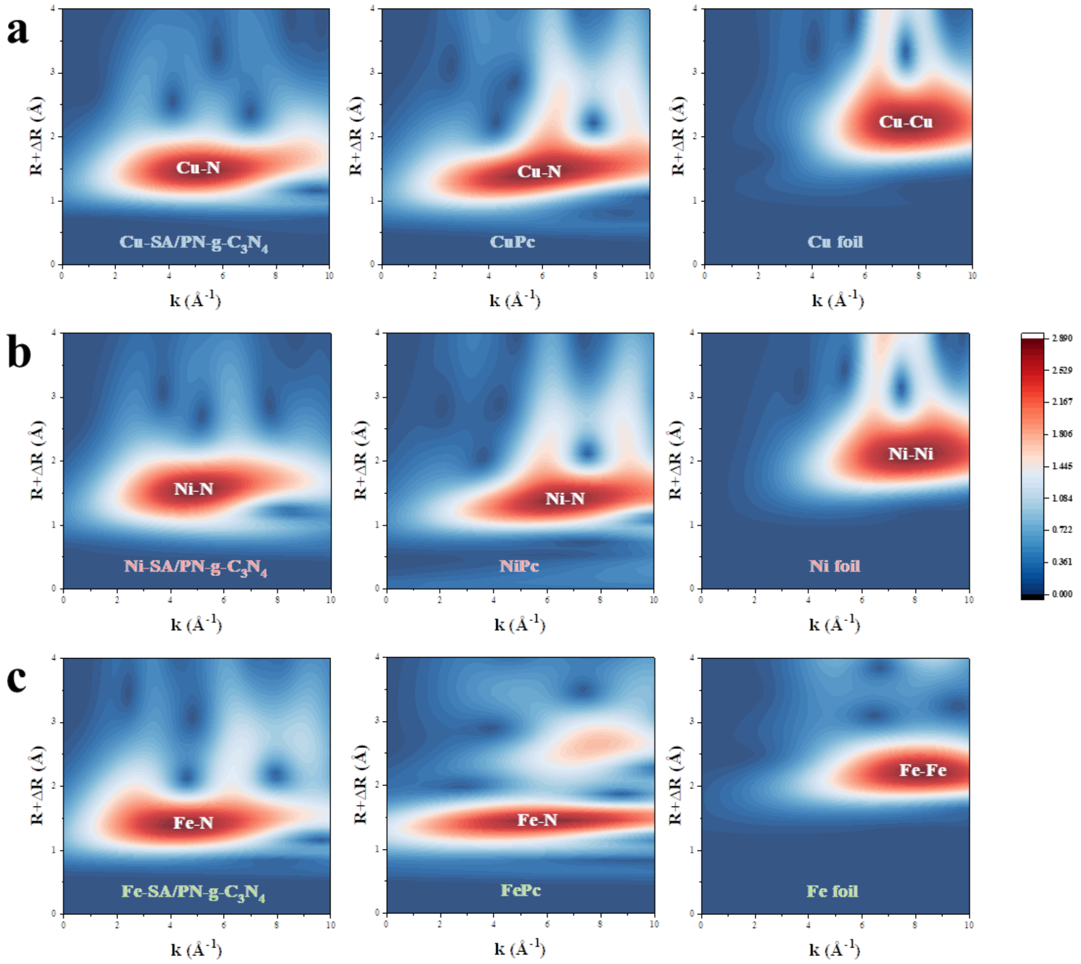

Supplementary Fig. 8 | Wavelet Transform analysis. a-c, WT-EXAFS plots of Cu **(a)**, Ni **(b)**, and Fe **(c)** series group. Each group consists of three samples: TM-SA/PN-g-C_3N_4, TMPc, and TM foil (TM=Cu, Ni, Fe).

For Wavelet Transform analysis, the χ(k) exported from Athena was imported into the Hama Fortran code. The parameters were listed as follows: R range, 1-4 Å, k range, 0-10.0 $Å^{-1}$ for sample; k weight, 3; and Morlet function with κ=5, σ=1 was used as the mother wavelet to provide the overall distribution.

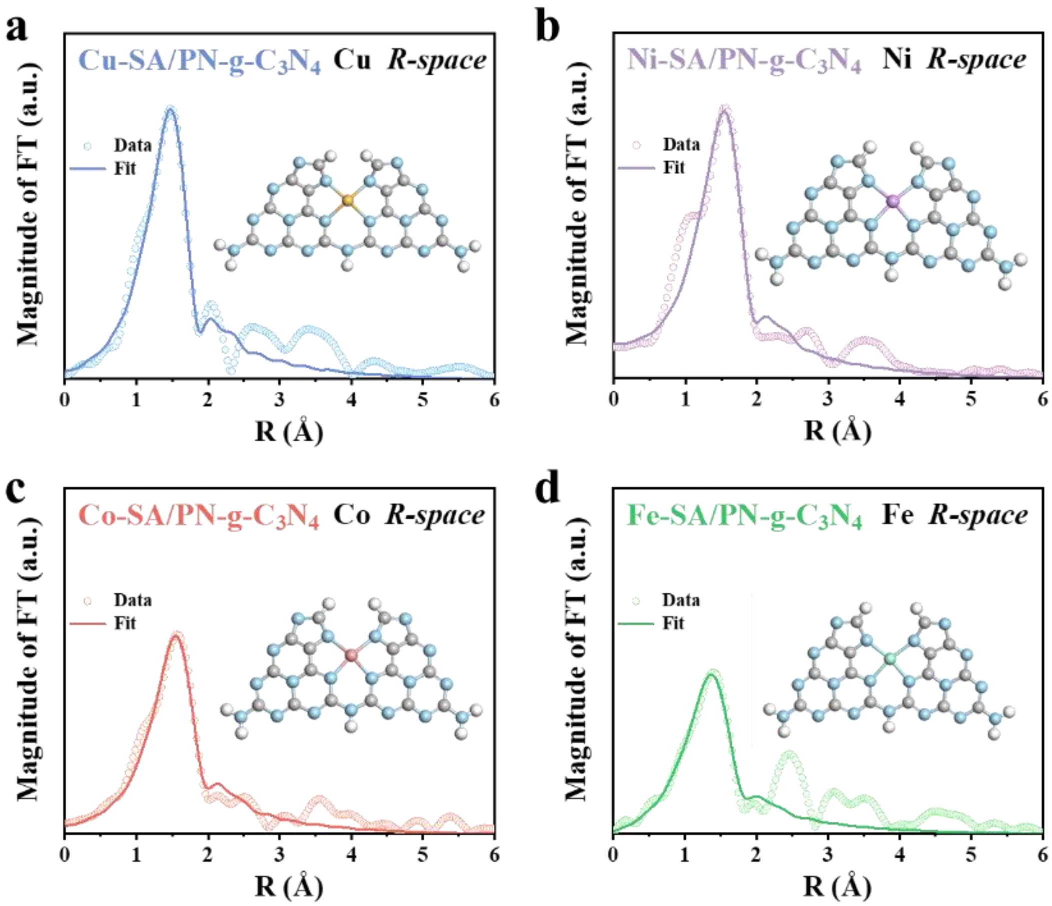

Supplementary Fig. 9 | EXAFS fitting. a-d, Corresponding EXAFS fitting curves of Cu-SA/PN-g-C_3N_4 **(a)**, Ni-SA/PN-g-C_3N_4 **(b)**, Co-SA/PN-g-C_3N_4 **(c)**, and Fe-SA/PN-g-C_3N_4 **(d)** at *R*-space.

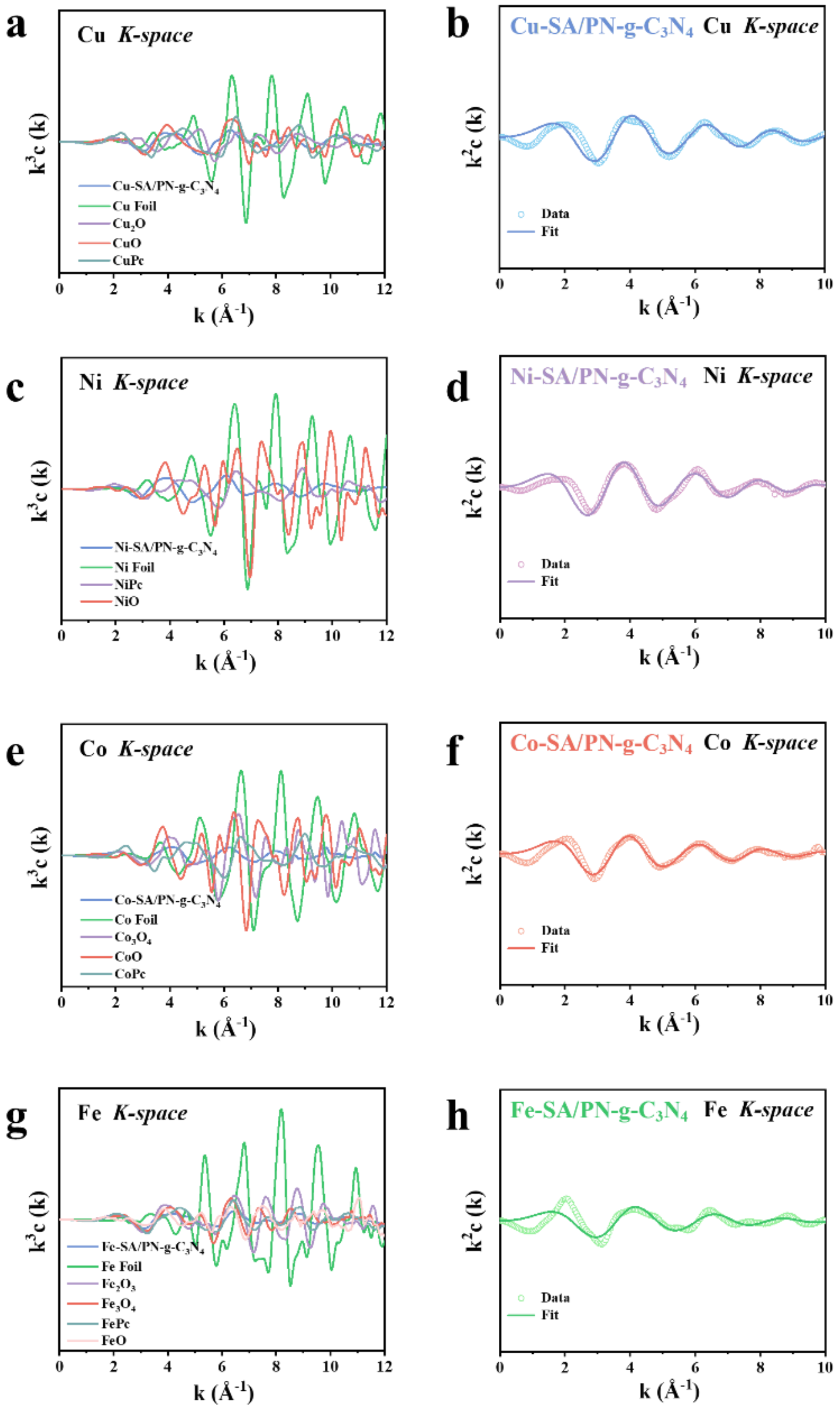

Supplementary Fig. 10 | EXAFS K-space and corresponding fitting. a-h, EXAFS *K*-space and corresponding fitting curves of Cu-SA/PN-g-C_3N_4 **(a, b)**, Ni-SA/PN-g-C_3N_4 **(c, d)**, Co-SA/PN-g-C_3N_4 **(e, f)**, and Fe-SA/PN-g-C_3N_4 **(g, h)**.

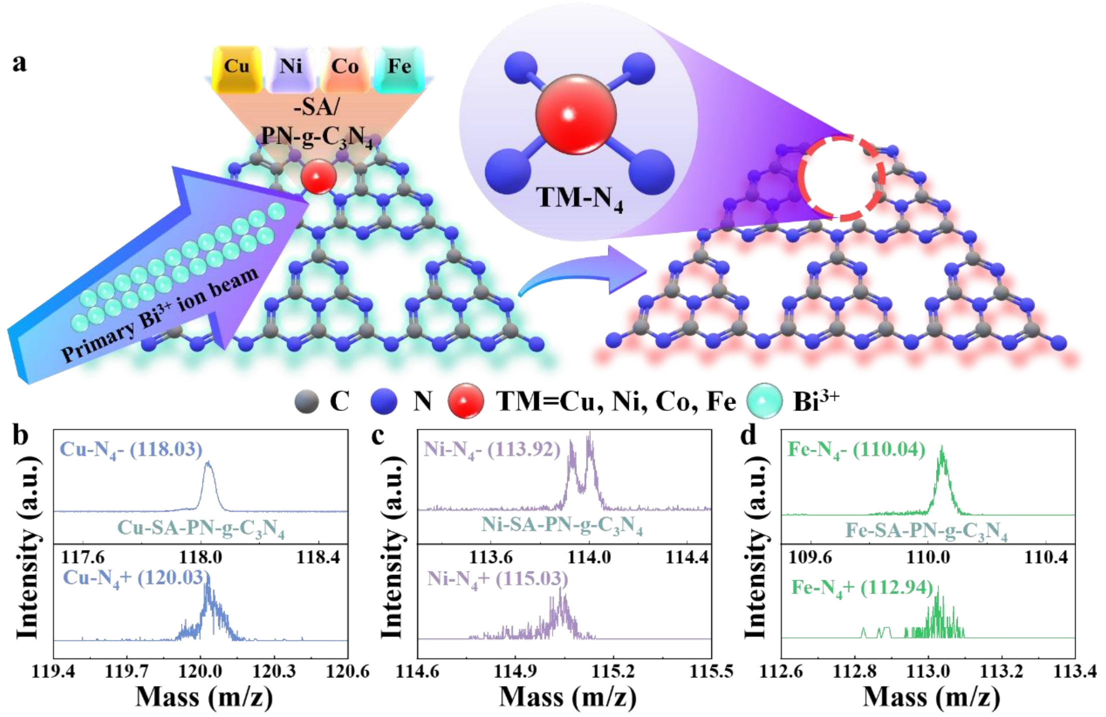

Supplementary Fig. 11 | Identification of TM-N_4 structures using TOF-SIMS. a, The illustration of the working mechanism of TOF-SIMS. **b-d**, TOF-SIMS high-resolution negative-ion and positive-ion spectra for Cu-N_4 **(b)**, Ni-N_4 **(c)**, Fe-N_4 **(d)** structural units.

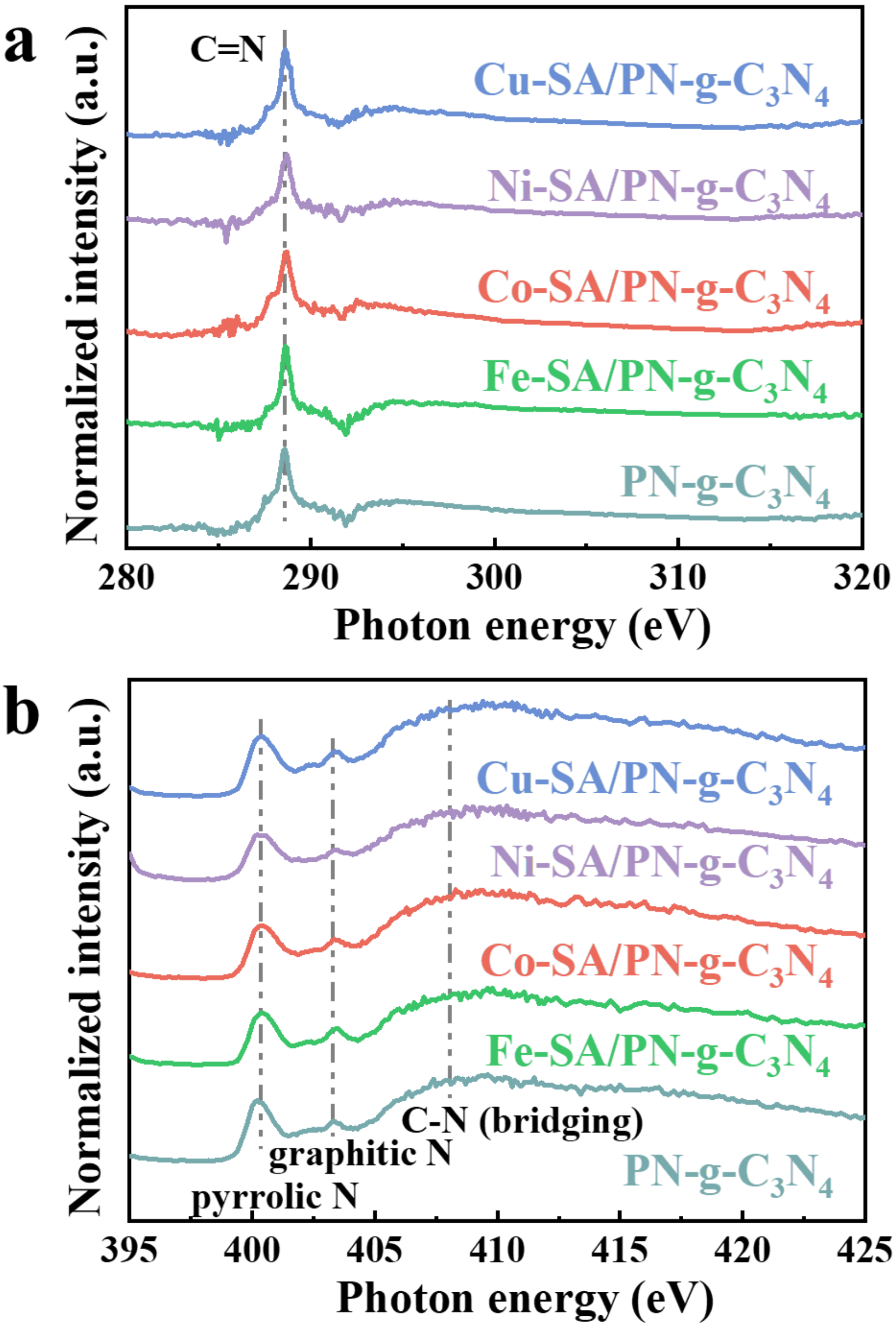

Supplementary Fig. 12 | XAS analysis of materials. a, C *K*-edge and **b,** N *K*-edge XANES spectra of the TM (Cu, Ni, Co, Fe)-SA/PN-g-C_3N_4 catalysts.

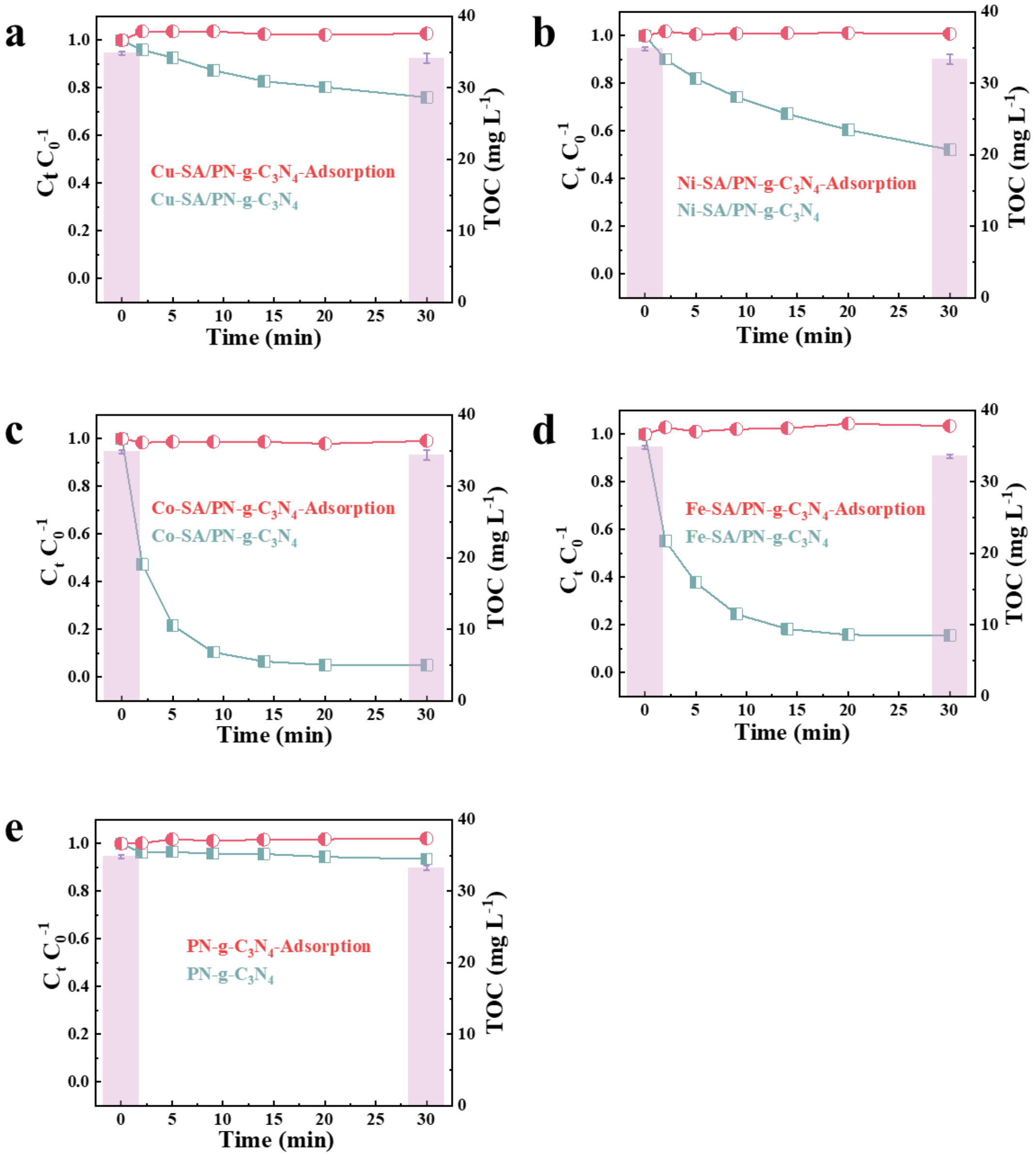

Supplementary Fig. 13 | Adsorption control of catalyst. a-e, Adsorption kinetics and TOC measurements of Cu **(a)**, Ni **(b)**, Fe **(c)**, Co **(d)**-SA/PN-g-C_3N_4, and PN-g-C_3N_4 **(e)**. Reaction conditions: [Cat.] = 1.0 g L^{-1}, [PMS] = 1.0 mM, [PhOH] = 0.5 mM, initial pH = 7.0, T = 25 ± 2 °C. Error bars represent the standard deviation, obtained by repeating the experiment three times.

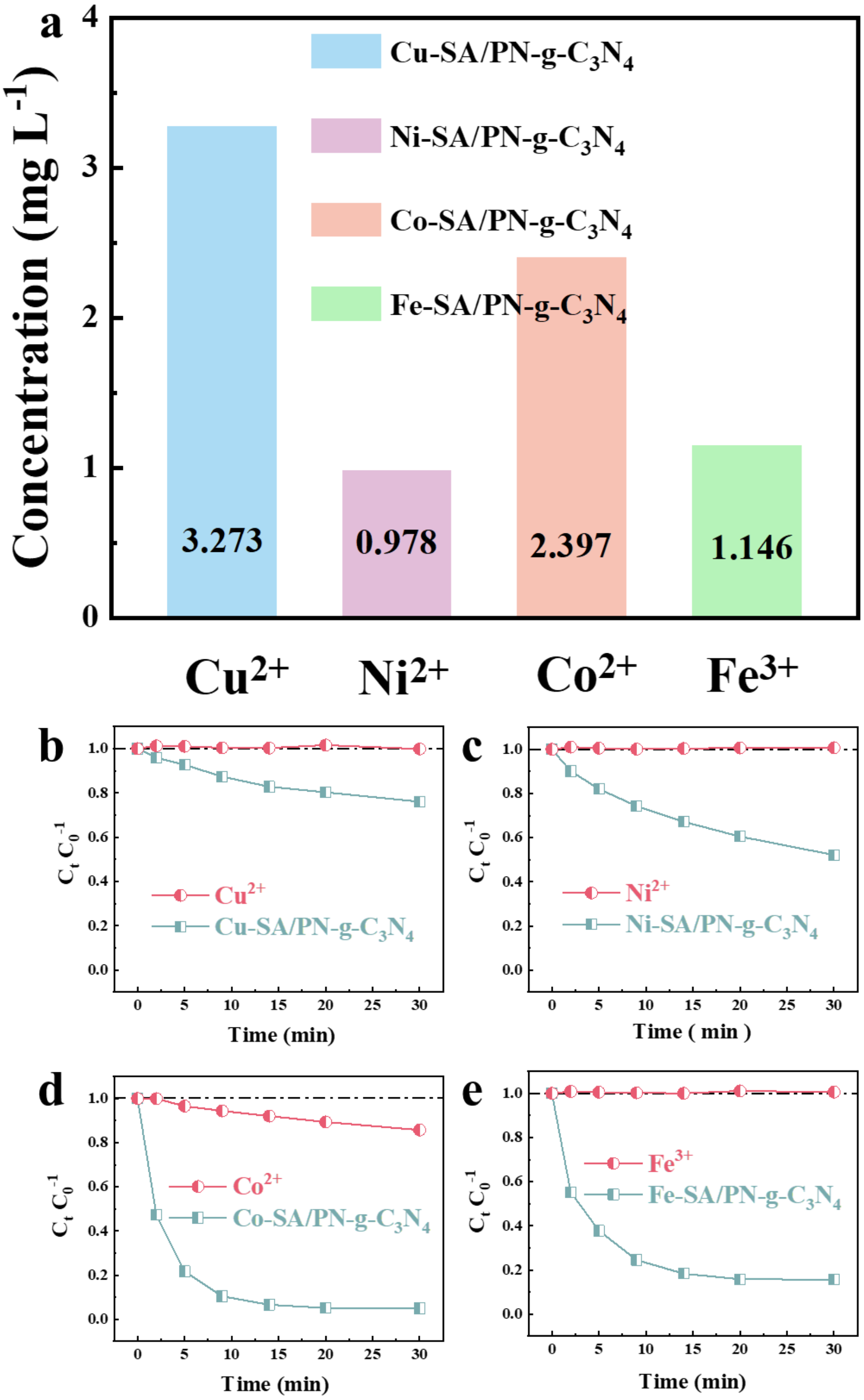

Supplementary Fig. 14 | The leaching control of catalyst. a, Leaching of TM (Cu, Ni, Co, Fe) metal ions. **b-e** Comparison of homogeneous and heterogeneous kinetics of Cu **(b)**, Ni **(c)**, Co **(d)**, and Fe **(e)**-SA/PN-g-C_3N_4. Reaction conditions: [PMS] = 1.0 mM, [PhOH] = 0.5 mM, initial pH = 7.0, T = 25 ± 2 °C.

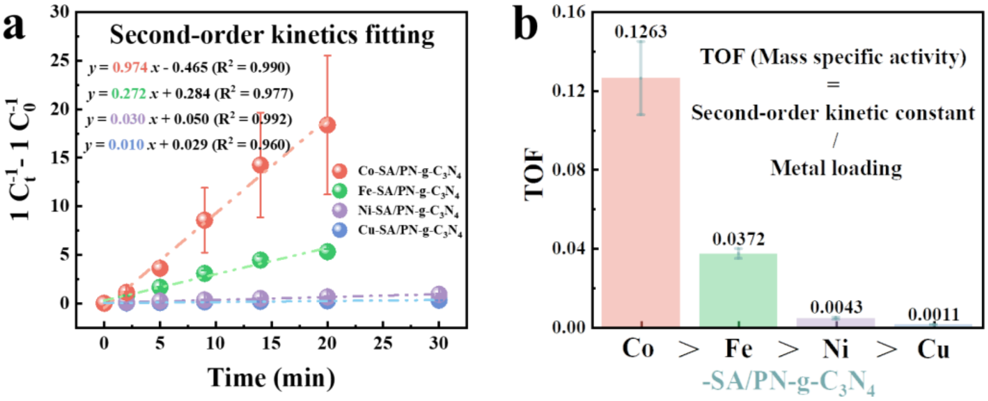

Supplementary Fig. 15 | Kinetic analysis and TOF calculation for TM-SACs in PMS activation. **a,** The second-order kinetic fitting results. **b**, Calculation of TOF (mass specific activity). Reaction conditions: [Cat.] = 1.0 g L^{-1}, [PMS] = 1.0 mM, [PhOH] = 0.5 mM, initial pH = 7.0, T = 25 ± 2 °C. Error bars represent the standard deviation, obtained by repeating the experiment three times.

To evaluate the mass-specific activity of the four TM-SACs, the turnover frequency (TOF) values were calculated using the second-order rate constant and the loading amounts of single atoms. The results show that the TOF sequence was consistent with the apparent catalytic activity sequence of Co>Fe>Ni>Cu.

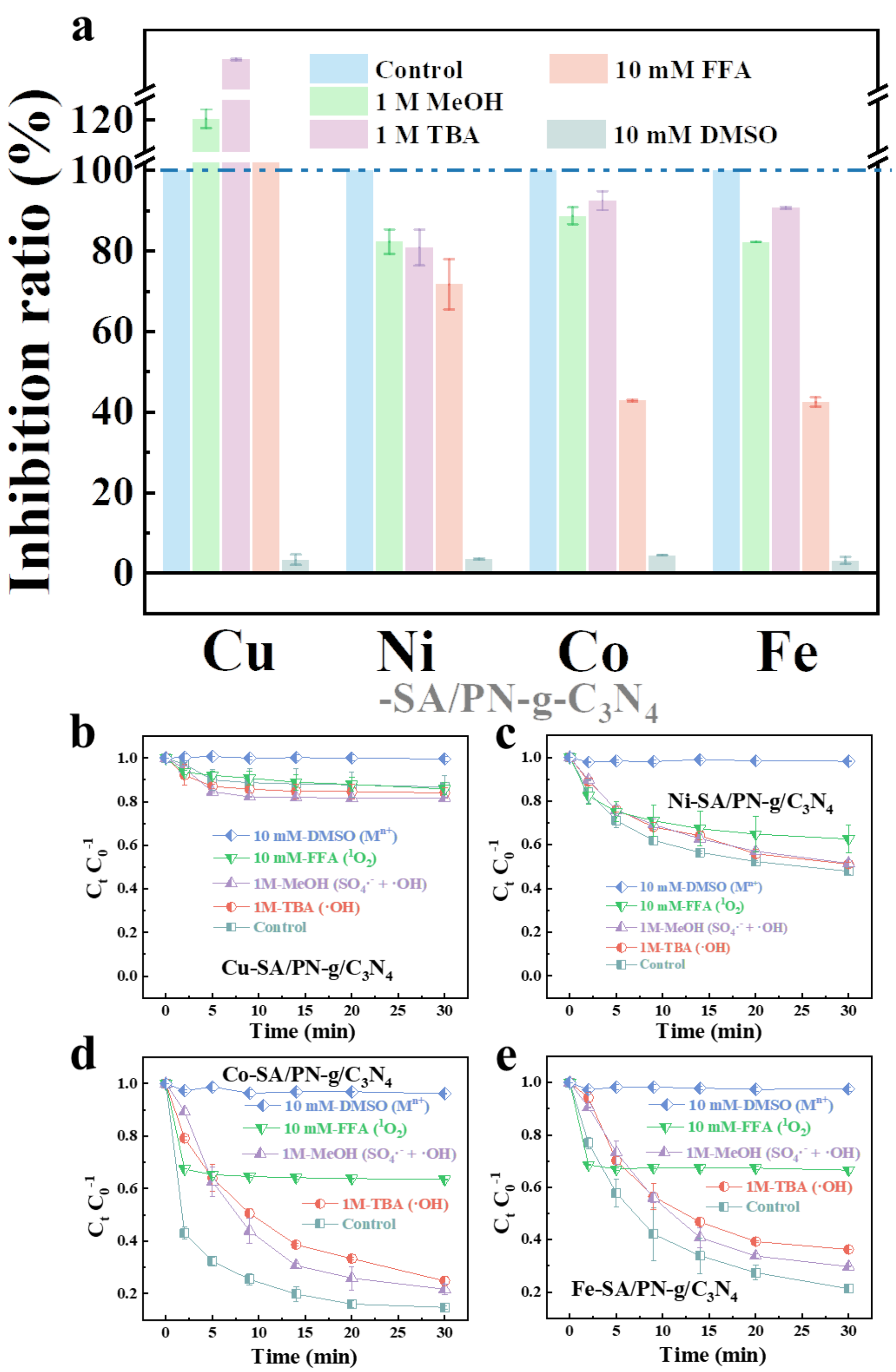

Supplementary Fig. 16 | Inhibitor test. a-e, Quenching experiments of Cu **(b)**, Ni **(c)**, Co **(d)**, and Fe **(e)**-SA/PN-g-C_3N_4. Reaction conditions: [Cat.] = 1.0 g L^{-1}, [PMS] = 1.0 mM, [PhOH] = 0.5 mM, [MeOH] = [TBA] = 1.0 M, [DMSO] = [FFA] =10 mM, initial pH = 7.0, T = 25 ± 2 °C. Error bars represent the standard deviation, obtained by repeating the experiment two times.

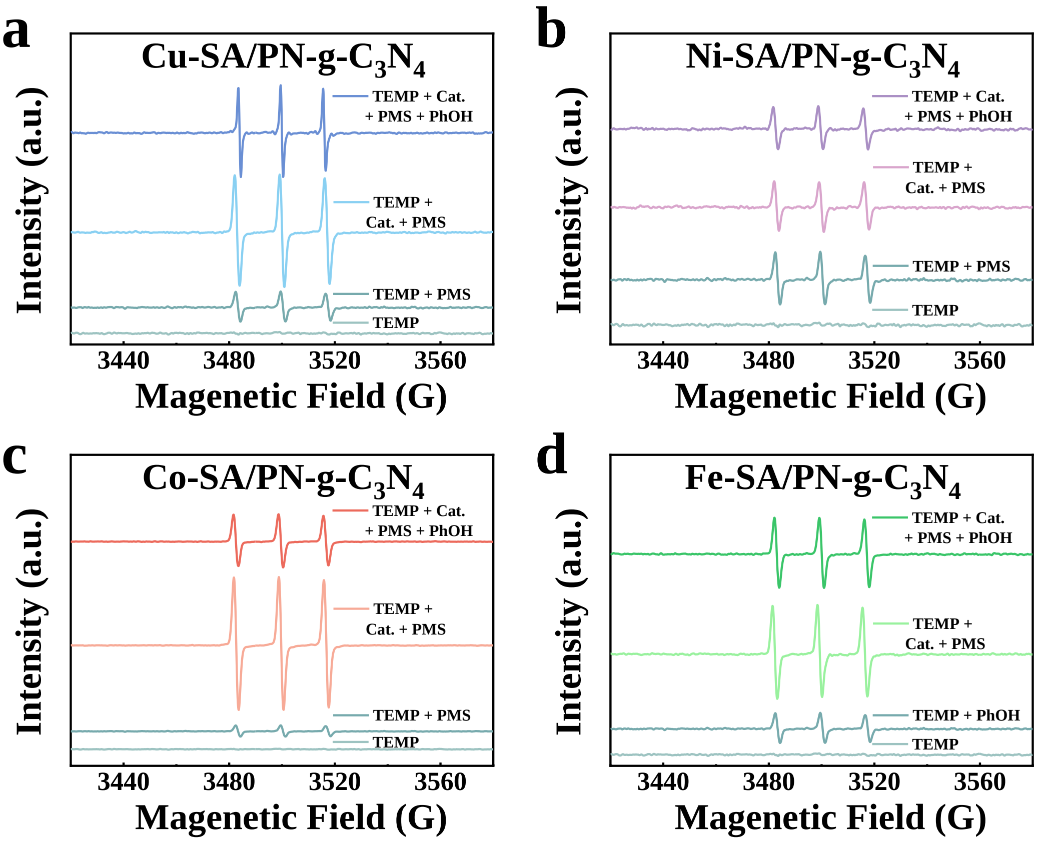

Supplementary Fig. 17 | EPR spectrum of $^{1}O_2$. a-d, EPR spectra of TM-SA/PN-g-C_3N_4/PMS/TEMP system. Reaction conditions: [Cat.] = 1.0 g L^{-1}, [PMS] = 1.0 mM, [PhOH] = 0.5 mM, [TEMP] = 10 mM, initial pH = 7.0, T = 25 ± 2 °C.

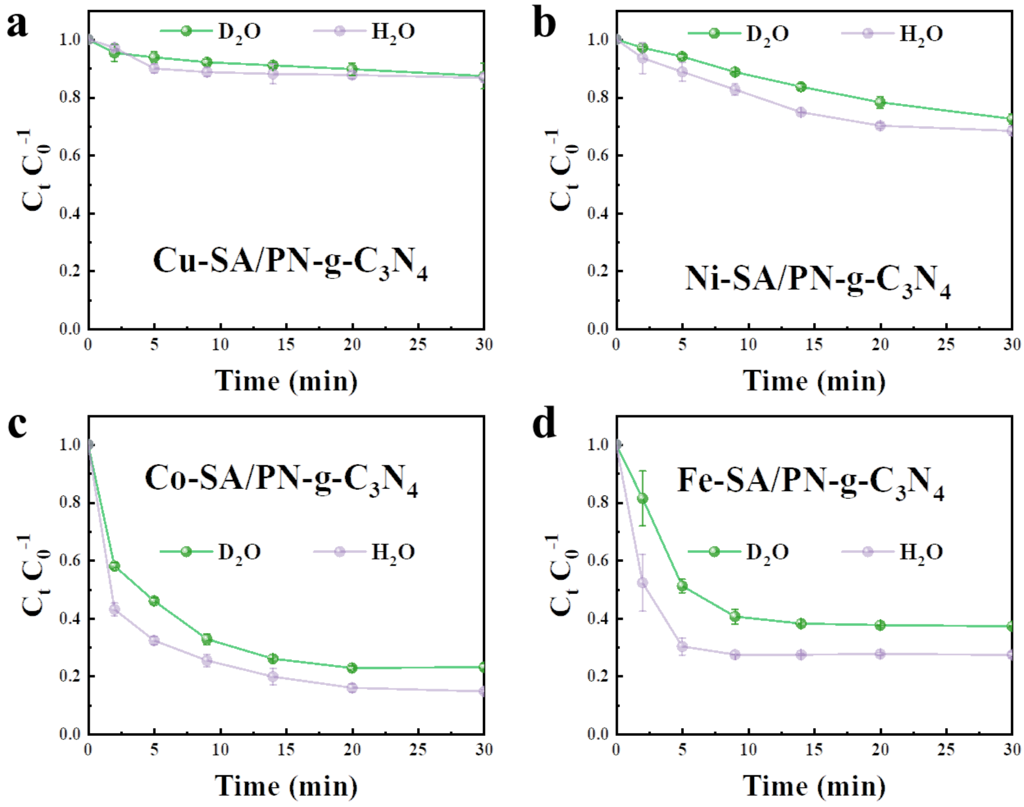

Supplementary Fig. 18 | Solvent exchange experiment verification of $^1O_2$. a-d, Degradation performances of PhOH in H_2O and D_2O solvents by Cu **(a)**, Ni **(b)**, Co **(c)**, and Fe **(d)**-SA/PN-g-C_3N_4. Reaction conditions: [Cat.] = 1.0 g L^{-1}, [PMS] = 1.0 mM, [PhOH] = 0.5 mM, initial pH = 7.0, T = 25 ± 2 °C. Error bars represent the standard deviation, obtained by repeating the experiment two times.

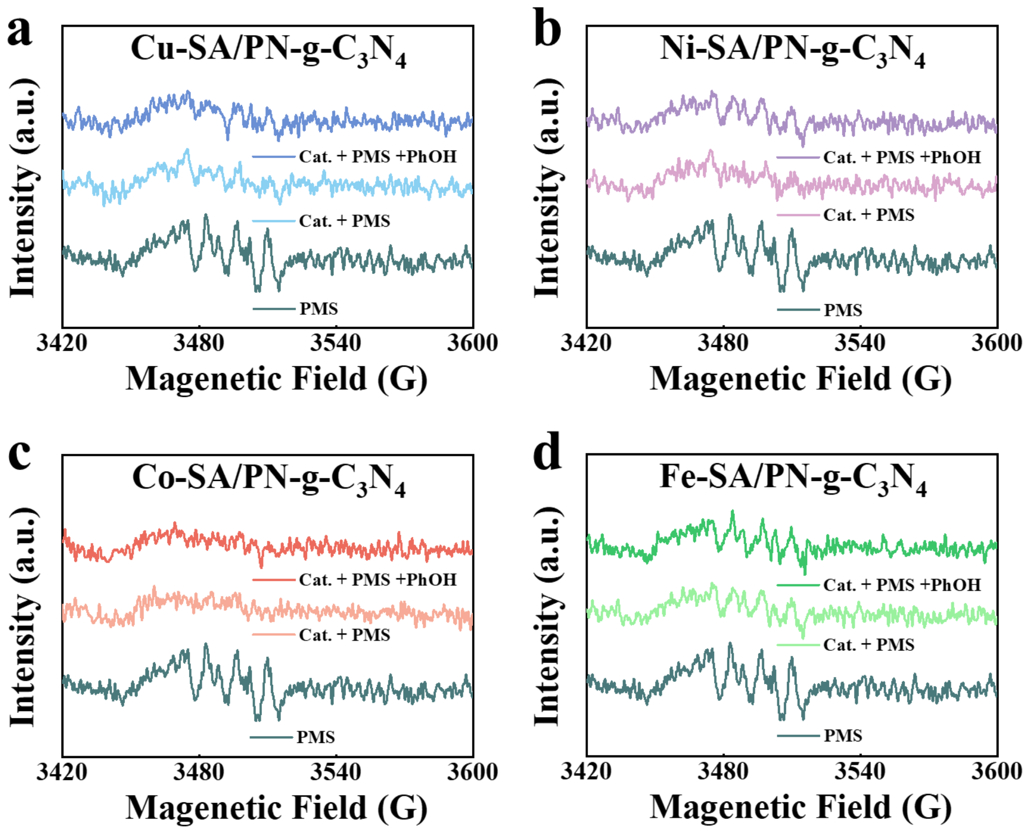

Supplementary Fig. 19 | EPR spectra of $O_2^{\bullet -}$. a-d, EPR spectra of $O_2^{\bullet -}$ in TM-SA/PN-g-C_3N_4/PMS/DMPO(MeOH) system. Reaction conditions: [Cat.] = 1.0 g/L, [PMS] = 1.0 mM, [PhOH] = 0.5 mM, [DMPO] = 100 mM, initial pH = 7.0, T = 25 ± 2 °C.

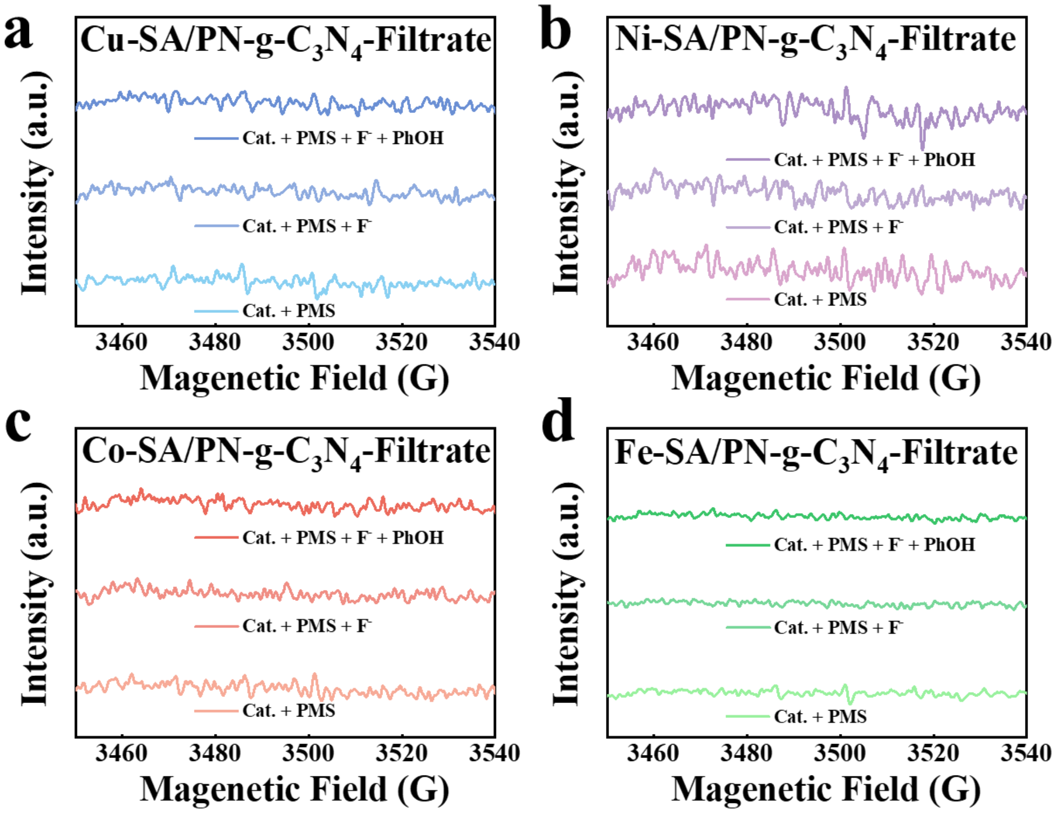

Supplementary Fig. 20 | EPR spectra of Surface free radicals. **a-d**, EPR spectra of TM-SA/PN-g-C_3N_4/PMS/DMPO/F^- system. Reaction conditions: [Cat.] = 1.0 g L^{-1}, [PMS] = 1.0 mM, [PhOH] = 0.5 mM, [DMPO] = 100 mM, [NaF] = 10 mM, initial pH = 7.0, T = 25 ± 2 °C.

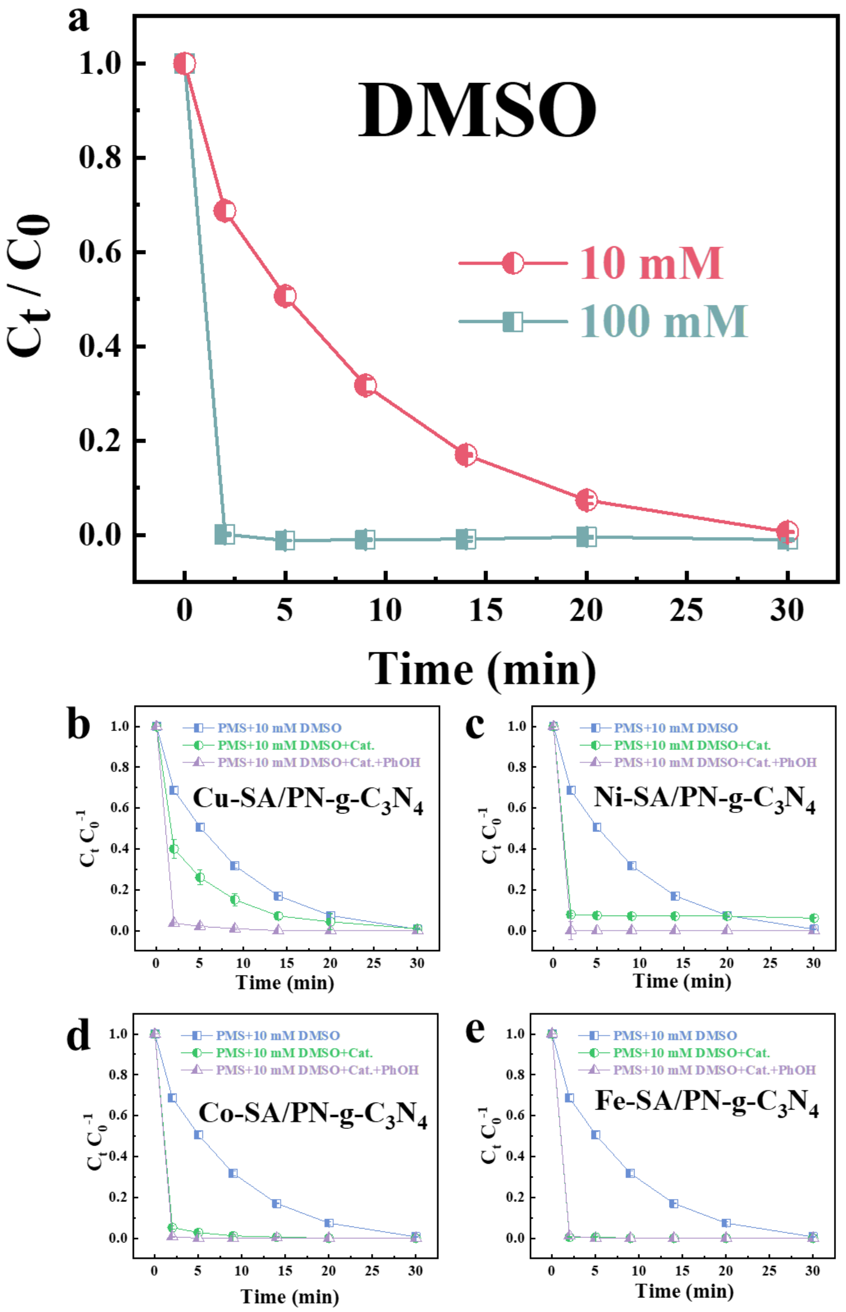

Supplementary Fig. 21 | Decomposition of PMS in presence of DMSO. a, PMS decomposition at different DMSO concentrations. **b-e**, PMS decomposition in the presence of catalyst, catalyst, and PhOH at 10 mM DMSO concentration of Cu **(b)**, Ni **(c)**, Co **(d)**, and Fe **(e)**-SA/PN-g-C_3N_4. Reaction conditions: [Cat.] = 1.0 g L^{-1}, [PMS] = 1.0 mM, [PhOH] = 0.5 mM, initial pH = 7.0, T = 25 ± 2 °C. Error bars represent the standard deviation, obtained by repeating the experiment two times.

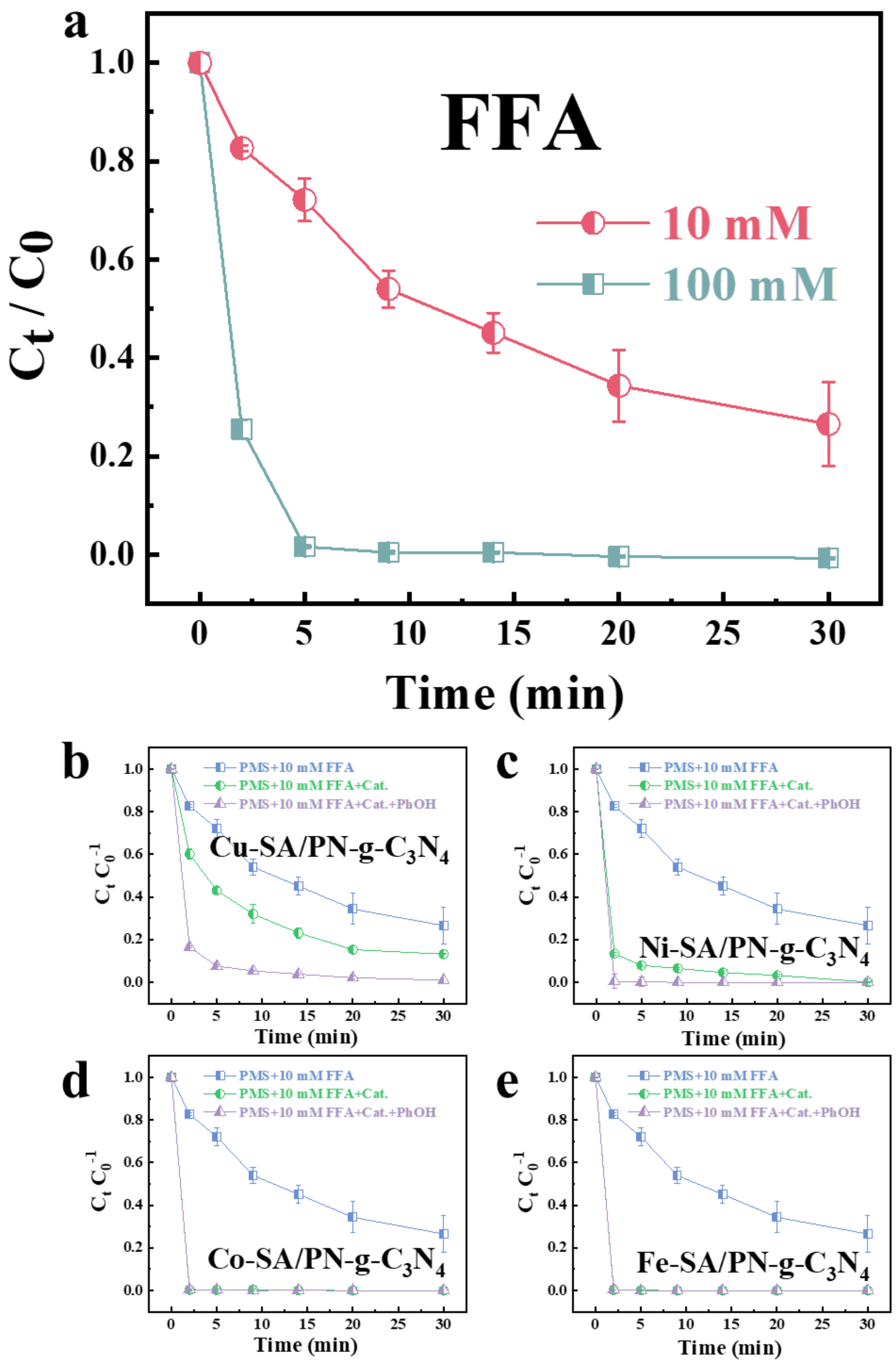

Supplementary Fig. 22 | Decomposition of PMS in presence of FFA. a, PMS decomposition at different FFA concentrations. **b-e**, PMS decomposition in the presence of catalyst, catalyst and PhOH at 10 mM FFA concentration of Cu **(b)**, Ni **(c)**, Co **(d)**, and Fe **(e)**-SA/PN-g-C_3N_4. Reaction conditions: [Cat.] = 1.0 g L^{-1}, [PMS] = 1.0 mM, [PhOH] = 0.5 mM, initial pH = 7.0, T = 25 ± 2 °C. Error bars represent the standard deviation, obtained by repeating the experiment two times.

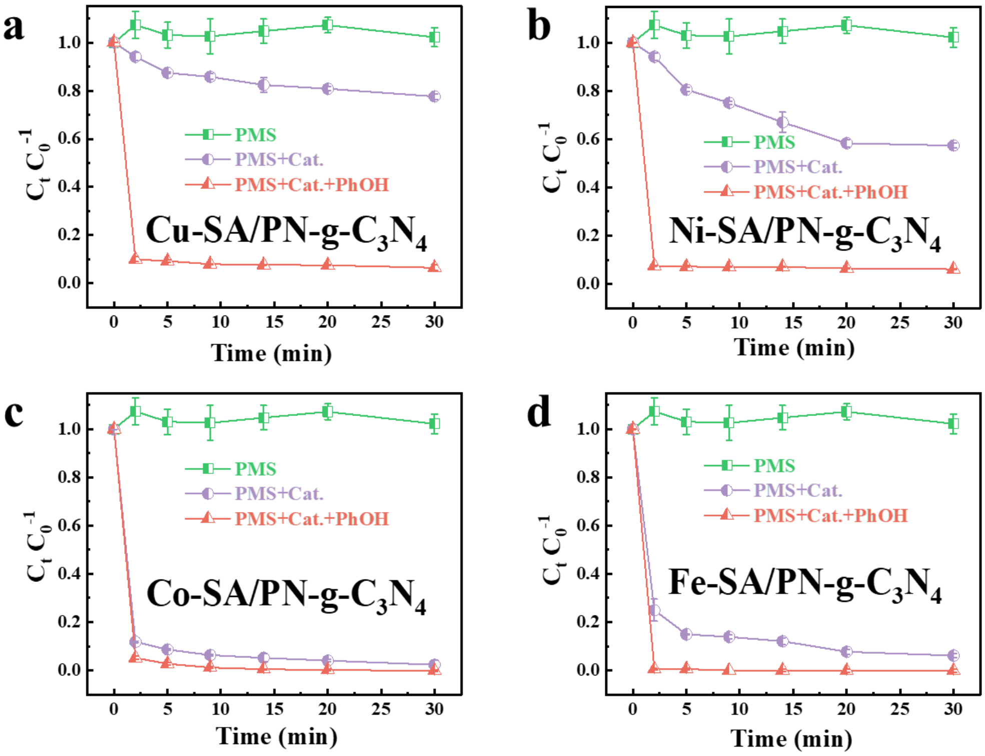

Supplementary Fig. 23 | Decomposition of PMS under reaction conditions. a-d, PMS decomposition in the presence of catalyst, catalyst, and PhOH of Cu **(a)**, Ni **(b)**, Co **(c)**, and Fe **(d)**-SA/PN-g-C_3N_4. Reaction conditions: [Cat.] = 1.0 g L^{-1}, [PMS] = 1.0 mM, [2, 6-M-PhOH] = 0.5 mM, initial pH = 7.0, T = 25 ± 2 °C. Error bars represent the standard deviation, obtained by repeating the experiment two times.

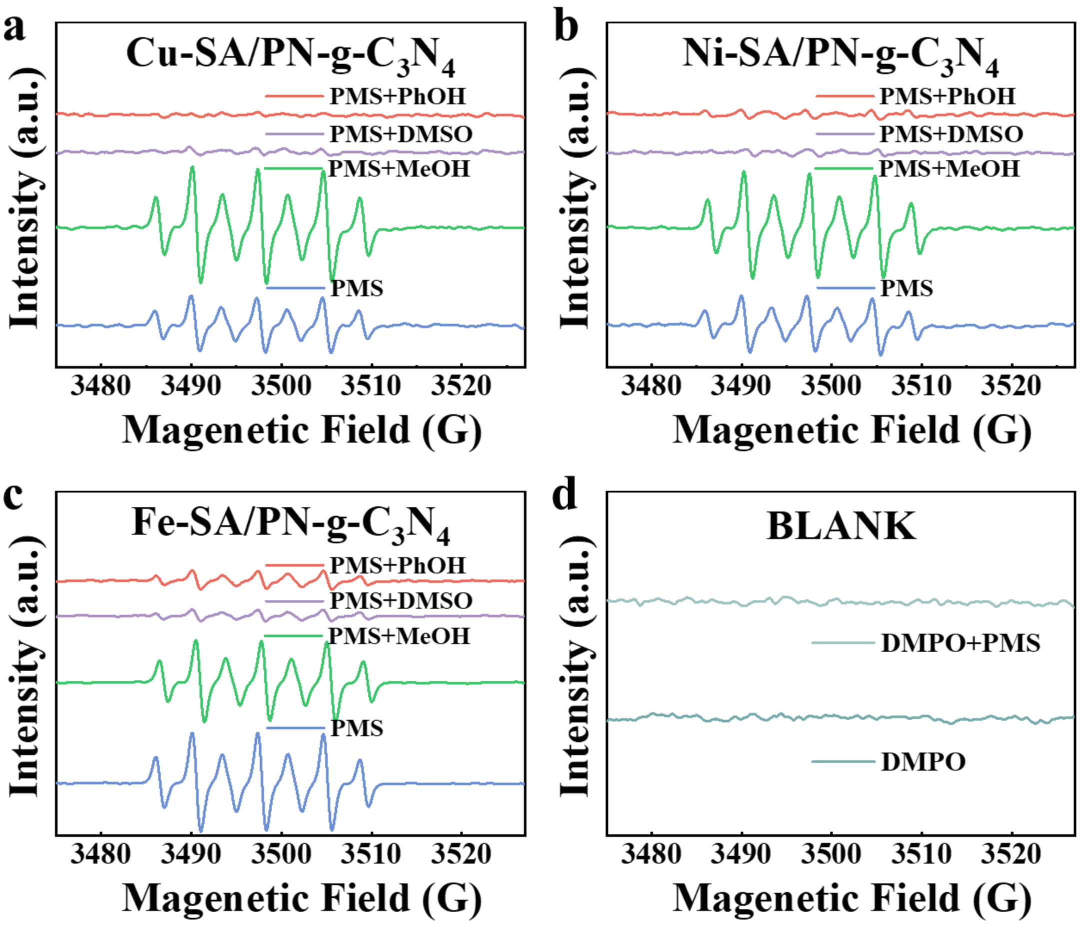

Supplementary Fig. 24 | EPR spectra of DMPO as a trapping agent. a-d, EPR spectra of TM (Cu **(a)**, Ni **(b)**, Fe **(c)**)-SA/PN-g-C_3N_4 & BLANK **(d)**/PMS/DMPO system. Reaction conditions: [Cat.] = 1.0 g L^{-1}, [PMS] = 1.0 mM, [PhOH] = 0.5 mM, [DMPO] = 100 mM, [MeOH] = 100 mM, initial pH = 7.0, T = 25 ± 2 °C.

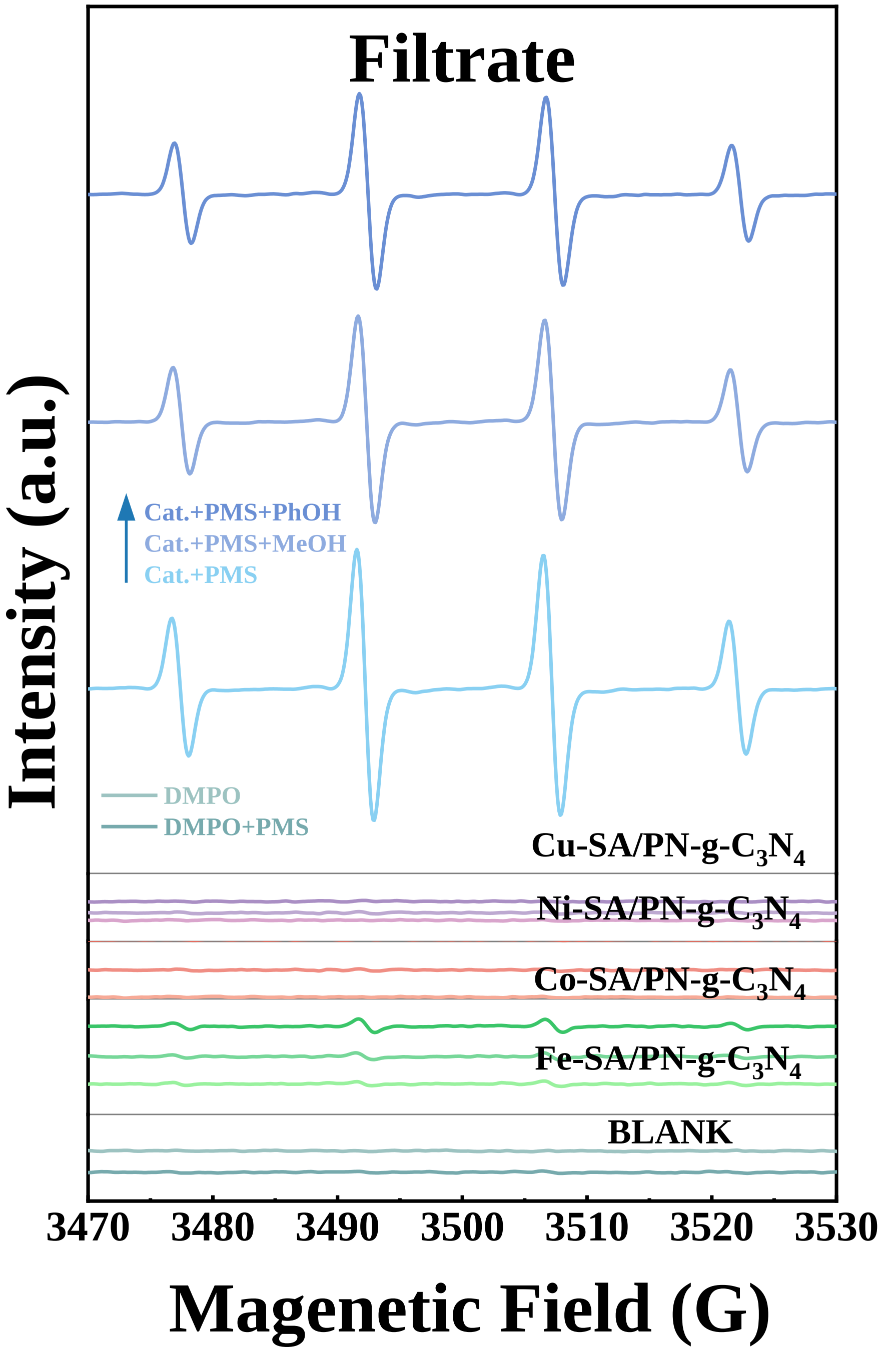

Supplementary Fig. 25 | EPR spectra of filtrate in TM-SA/PN-g-C_3N_4/PMS/DMPO system.
Reaction conditions: [Cat.] = 1.0 g L^{-1}, [PMS] = 1.0 mM, [PhOH] = 0.5 mM, [DMPO] = [MeOH] = 100 mM, initial pH = 7.0, T = 25 ± 2 °C.

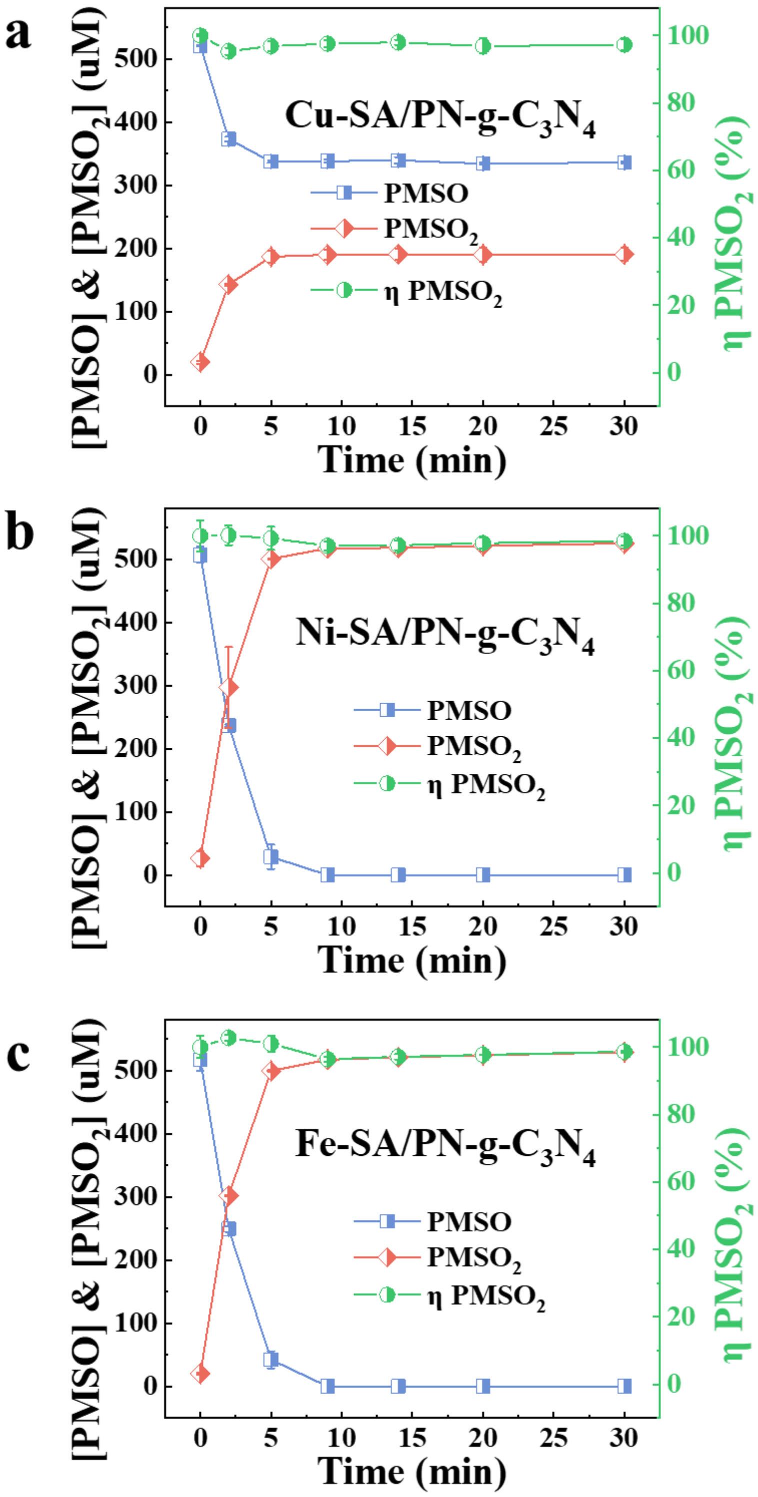

Supplementary Fig. 26 | PMSO probe experiment. a-c, Probe experiments of Cu **(a)**, Ni **(b)**, and Fe **(c)**-SA/PN-g-C_3N_4 system. Reaction conditions: [Cat.] = 1.0 g L^{-1}, [PMS] = 1.0 mM, [PMSO] = 0.5 mM, initial pH = 7.0, T = 25 ± 2 °C. Error bars represent the standard deviation, obtained by repeating the experiment two times.

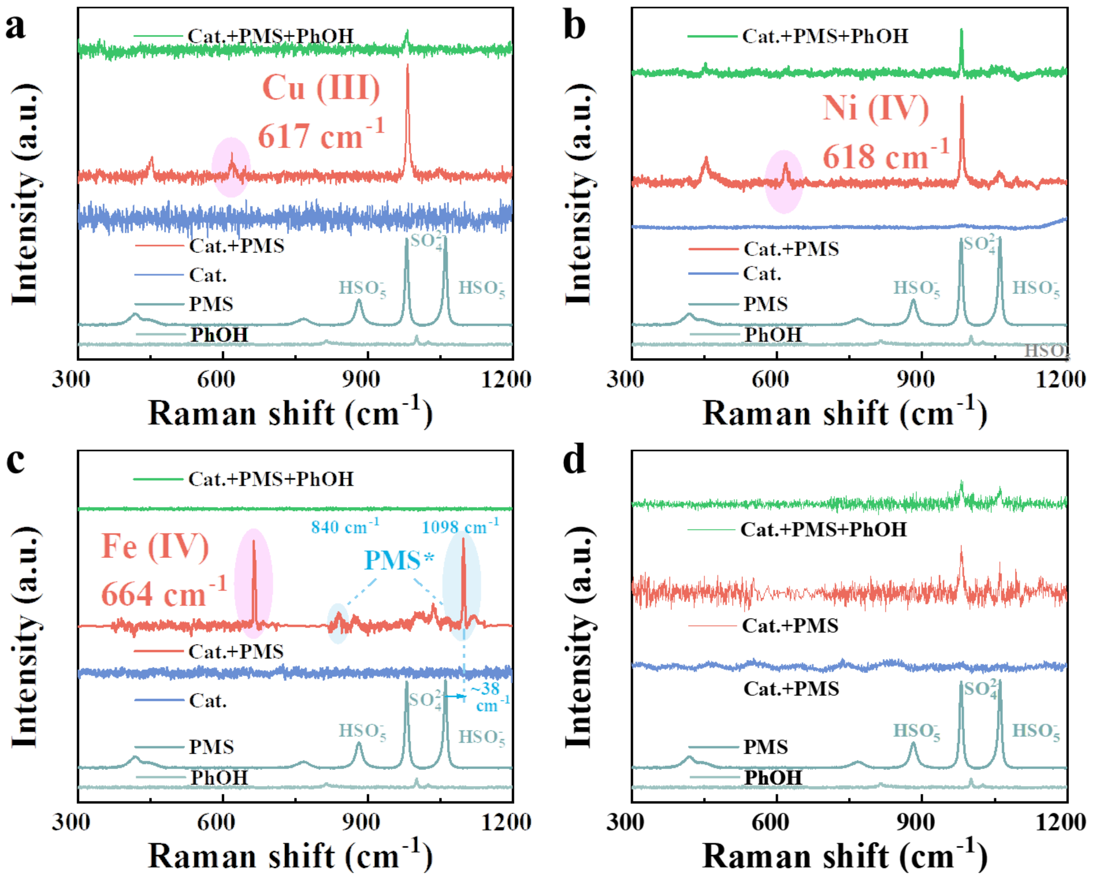

Supplementary Fig. 27 | In-situ Raman spectra. a-d, In-situ Raman spectra of TM (Cu **(a)**, Ni **(b)**, Fe **(c)**)-SA/PN-g-C_3N_4 & PN-g-C_3N_4 **(d)** /PMS/PhOH. The Cu(III), Ni(IV), and Fe(IV) species were marked with pink shades.

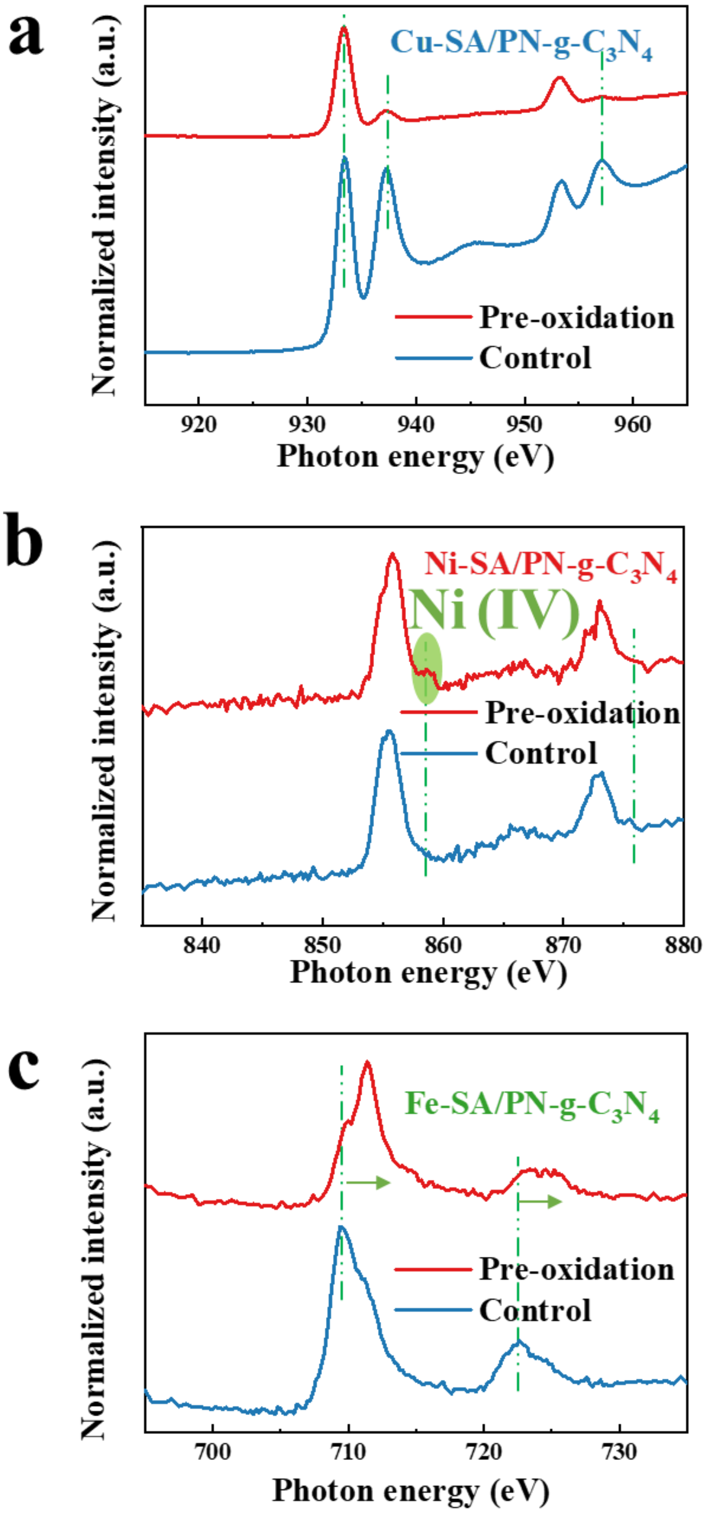

Supplementary Fig. 28 | XAS tracking during pre-oxidation. a-c, Pre-oxidation and original soft-XAS spectra of Cu **(a)**, Ni **(b)**, and Fe **(c)**-SA/PN-g-C_3N_4. The Ni(IV) species was marked with green shade.

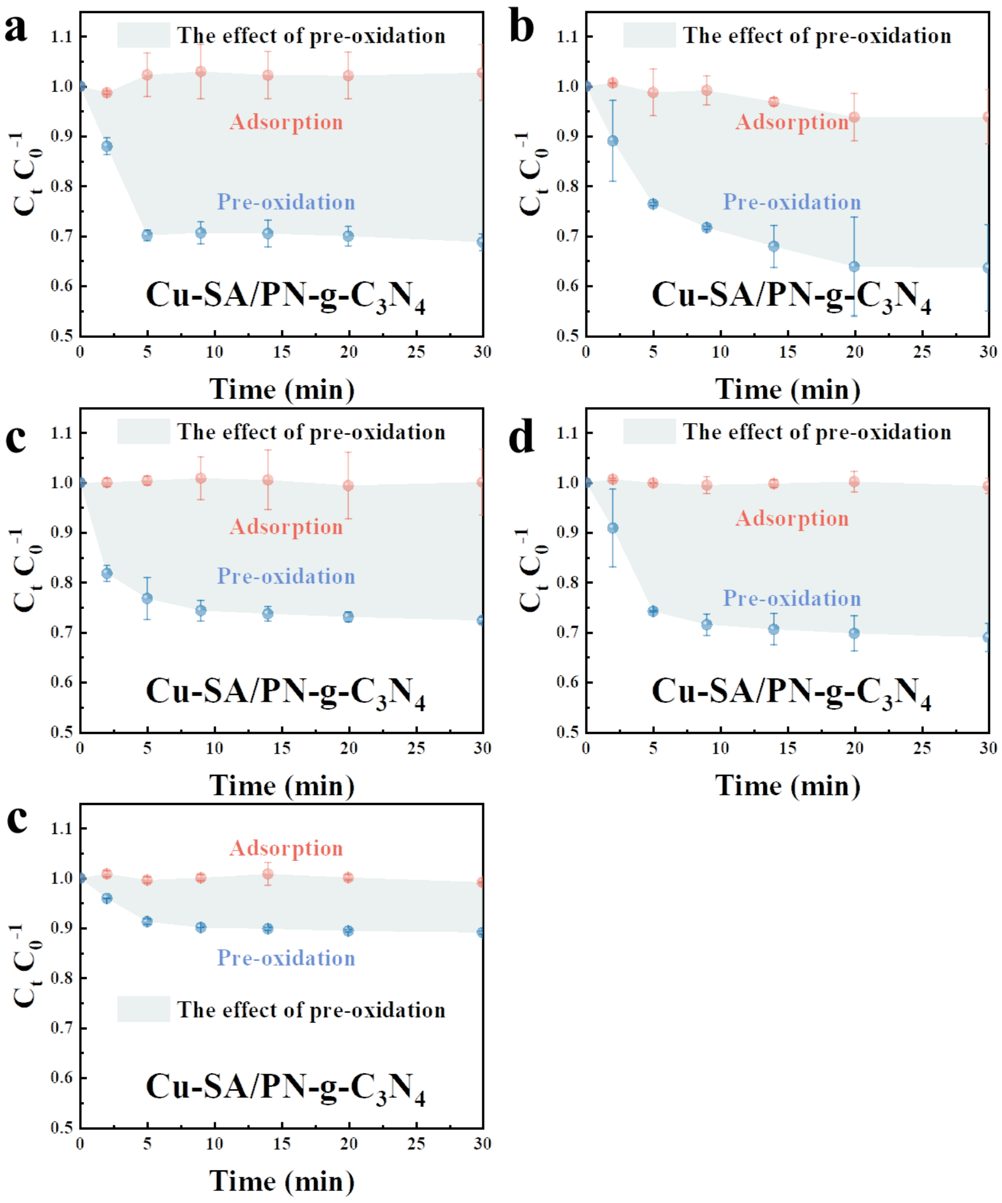

Supplementary Fig. 29 | Pre-oxidation experiment. a-e, The effect of pre-oxidation and adsorption on PhOH removal by Cu **(a)**, Ni **(b)**, Co **(c)**, Fe **(d)**-SA/PN-g-C_3N_4, and PN-g-C_3N_4 **(e)**. Reaction conditions: [Cat.] = 5.0 g L^{-1}, [PhOH] = 0.5 mM, [Pre-oxidation PMS] = 80 mM, initial pH = 7.0, T = 25 ± 2 °C. Error bars represent the standard deviation, obtained by repeating the experiment two times.

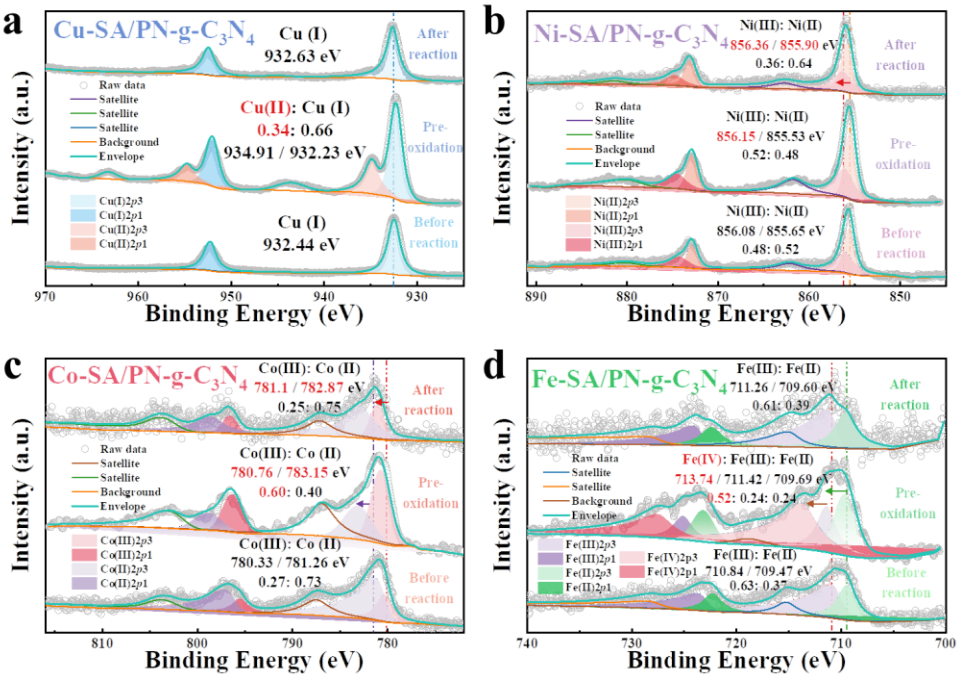

Supplementary Fig. 30 | XPS tracking during the reaction. a-d, The XPS Cu 2*p* **(a)**, Ni 2*p* **(b)**, Co 2*p* **(c)**, and Fe **(d)** 2*p* spectra of the TM-SA/PN-g-C_3N_4 before reaction, pre-oxidation and after reaction. Reaction conditions: [Cat.] = 1.0 g L^{-1}, [PMS] = 1.0 mM, [PhOH] = 0.5 mM, initial pH = 7.0, T = 25 ± 2 °C.

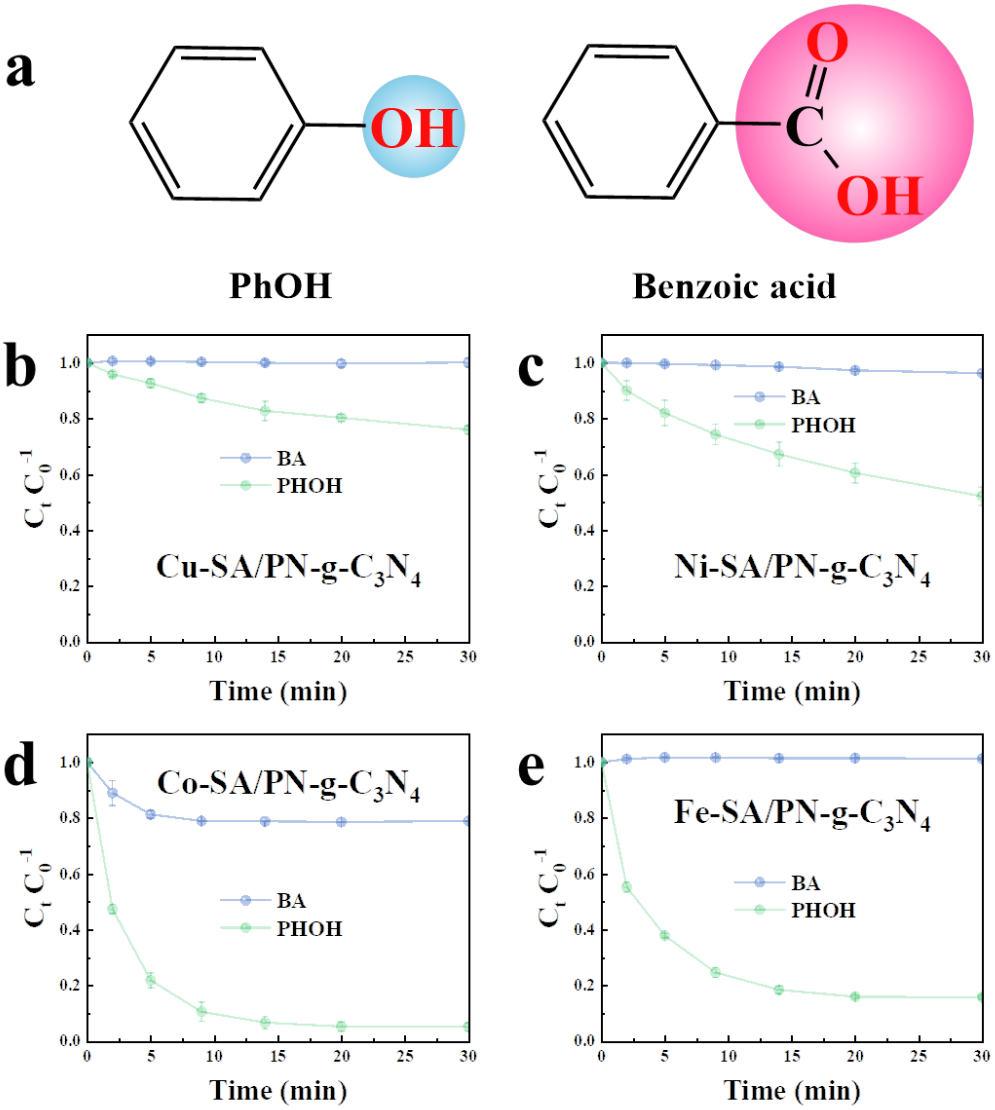

Supplementary Fig. 31 | Selectivity of the reaction to pollutant. a, Structural differences between PhOH and BA. **b-e**, Degradation of PhOH and BA through PMS activation by Cu **(b)**, Ni **(c)**, Co **(d)**, Fe **(e)**-SA/PN-g-C_3N_4. Reaction conditions: [Cat.] = 1.0 g L^{-1}, [PMS] = 1.0 mM, [PhOH/BA] = 0.5 mM, initial pH = 7.0, T = 25 ± 2 °C. Error bars represent the standard deviation, obtained by repeating the experiment two times.

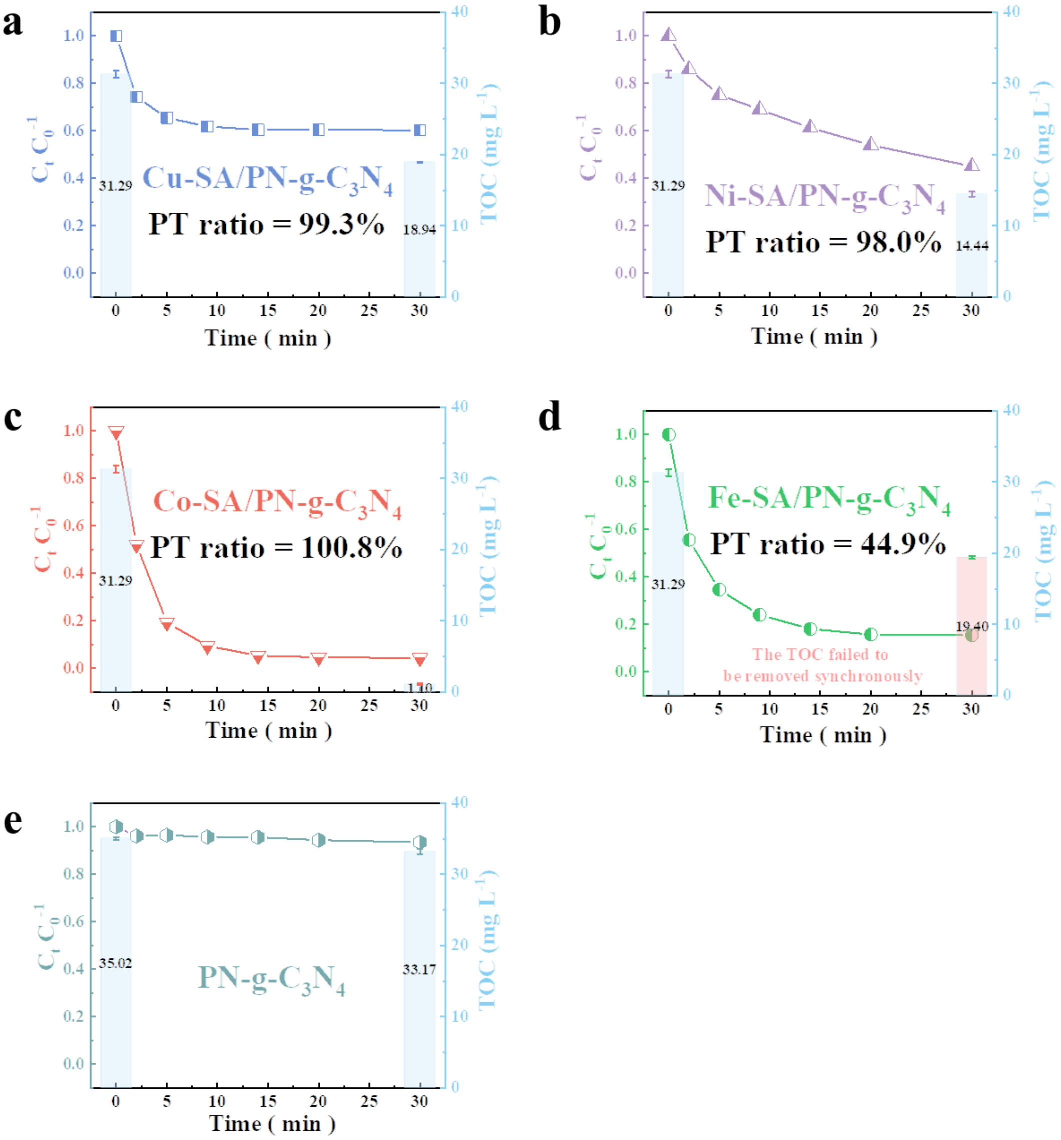

Supplementary Fig. 32 | PhOH degradation kinetics and TOC changes in the kinetic process. a-e, PhOH degradation, and TOC removal through PMS activation by the Cu **(a)**, Ni **(b)**, Co **(c)**, Fe **(d)**-SA/PN-g-C_3N_4, and PN-g-C_3N_4 **(e)** catalysts. Reaction conditions: [Cat.] = 1.0 g L^{-1}, [PMS] = 1.0 mM, [PhOH] = 0.5 mM, initial pH = 7.0, T = 25 ± 2 °C. The bar graphs correspond to the TOC values. Error bars represent the standard deviation, obtained by repeating the experiment two times.

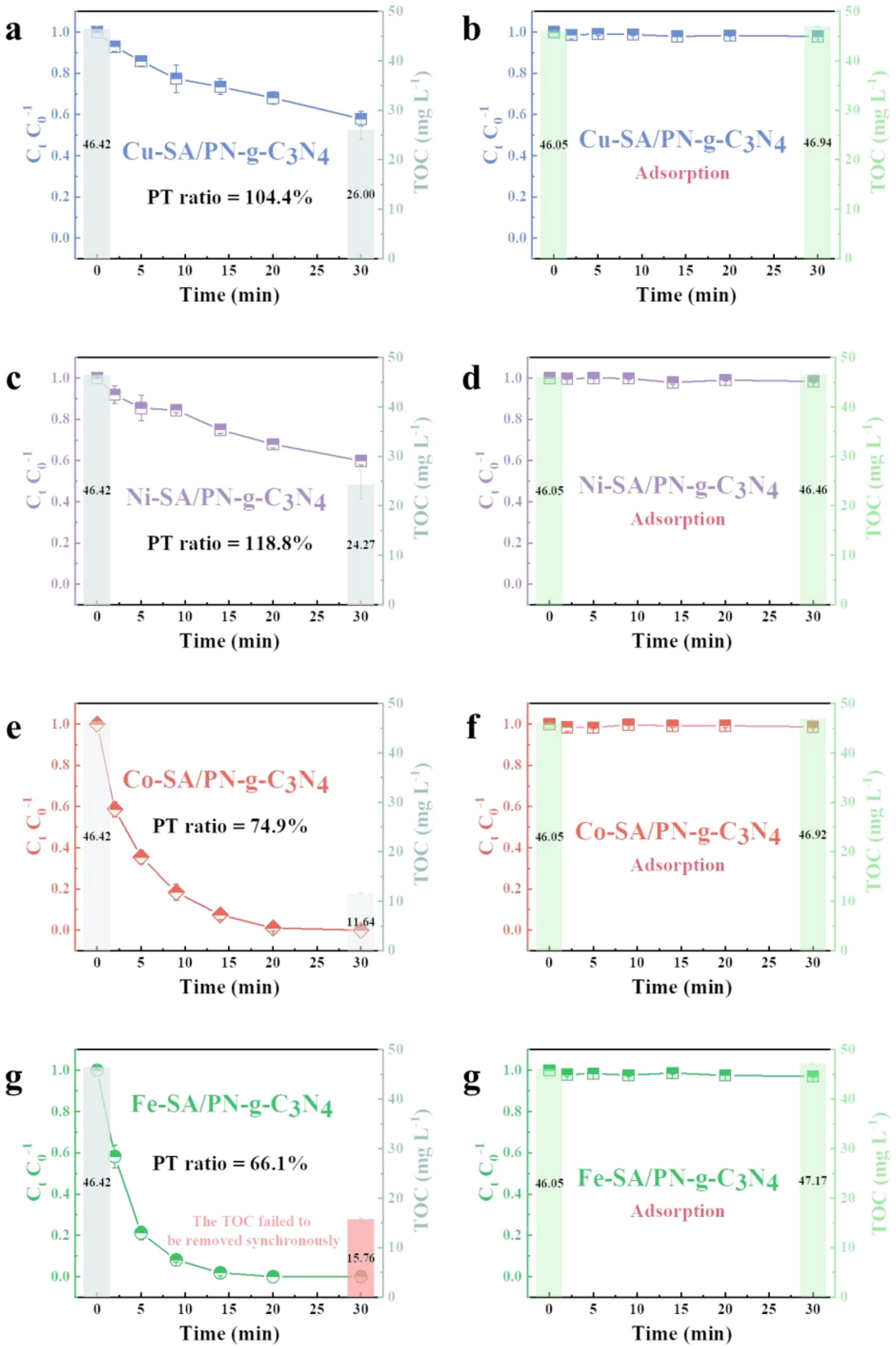

Supplementary Fig. 33 | 2, 6-M-PhOH degradation kinetics and TOC changes in the kinetic process. a-e, 2, 6-M-PhOH degradation and TOC removal through PMS activation by the Cu **(a)**, Ni **(c)**, Co **(e)**, Fe **(g)**-SA/PN-g-C_3N_4 catalysts, and the corresponding adsorption removal of 2, 6-M-PhOH and TOC (**b, d, f, h**). Reaction conditions: [Cat.] = 1.0 g L^{-1}, [PMS] = 1.0 mM, [2, 6-M-PhOH] = 0.5 mM, initial pH = 7.0, T = 25 ± 2 °C. The bar graphs correspond to the TOC values. Error bars represent the standard deviation, obtained by repeating the experiment two times.

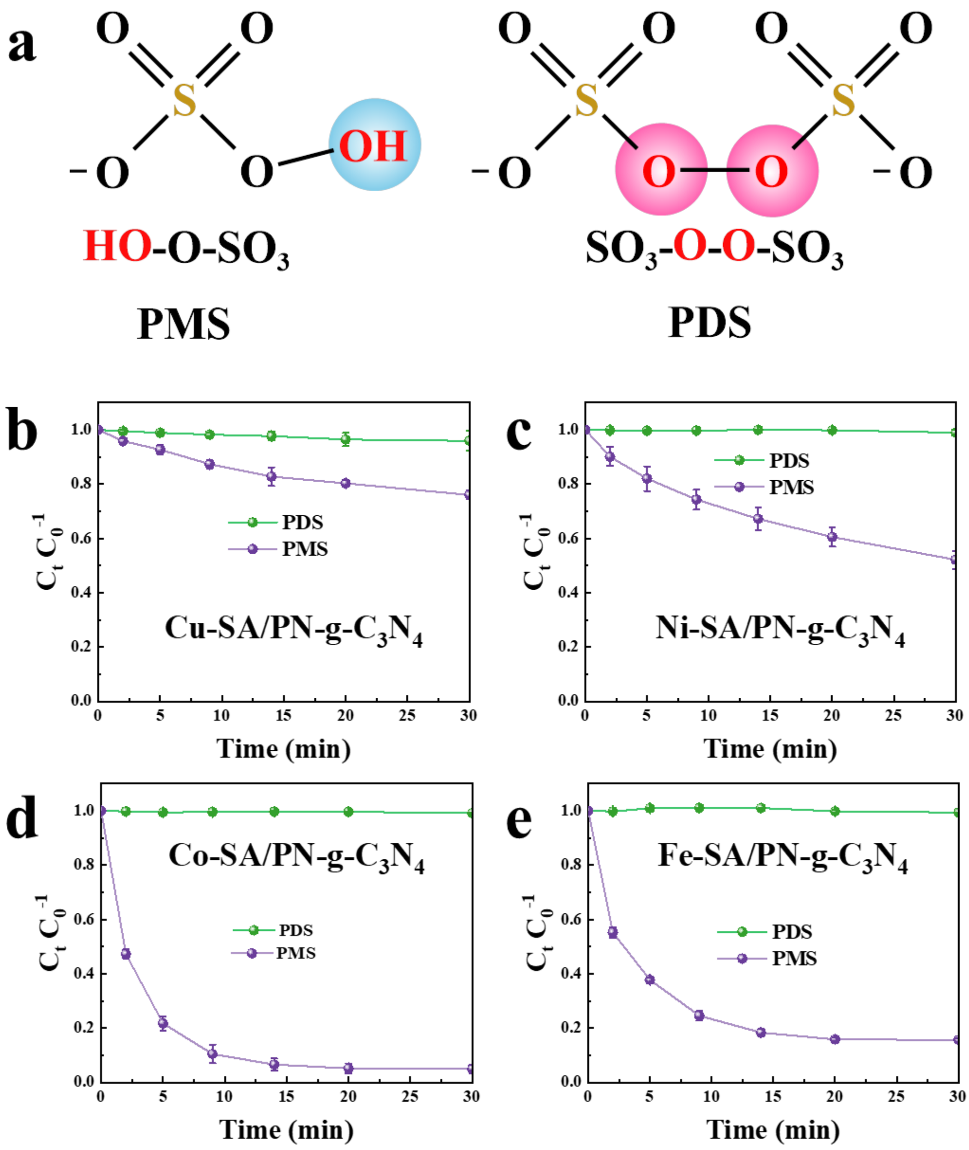

Supplementary Fig. 34 | Selectivity of the reaction to oxidant. a, Structural differences between PDS and PMS. **b-e**, Degradation of PhOH through PDS and PMS activation by Cu **(b)**, Ni **(c)**, Co **(d)**, and Fe **(e)**-SA/PN-g-C_3N_4. Reaction conditions: [Cat.] = 1.0 g L^{-1}, [PMS/PDS] = 1.0 mM, [PhOH] = 0.5 mM, initial pH = 7.0, T = 25 ± 2 °C. Error bars represent the standard deviation, obtained by repeating the experiment two times.

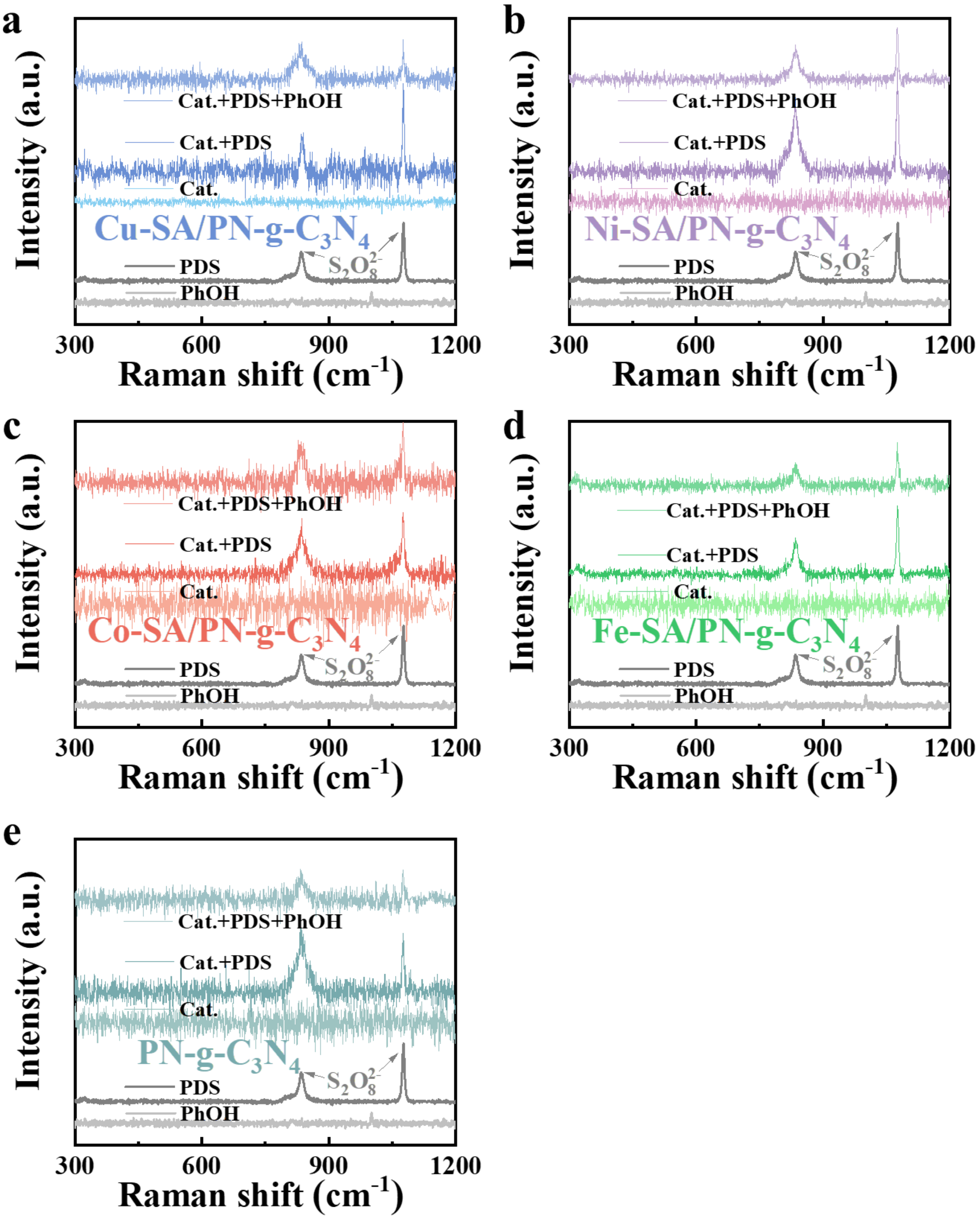

Supplementary Fig. 35 | In-situ Raman spectra of PDS system. a-e, In-situ Raman spectra of PDS or PhOH adsorption on TM (Cu **(a)**, Ni **(b)**, Co **(c)**, Fe **(d)**)-SA/PN-g-C_3N_4 & PN-g-C_3N_4 **(e)**.

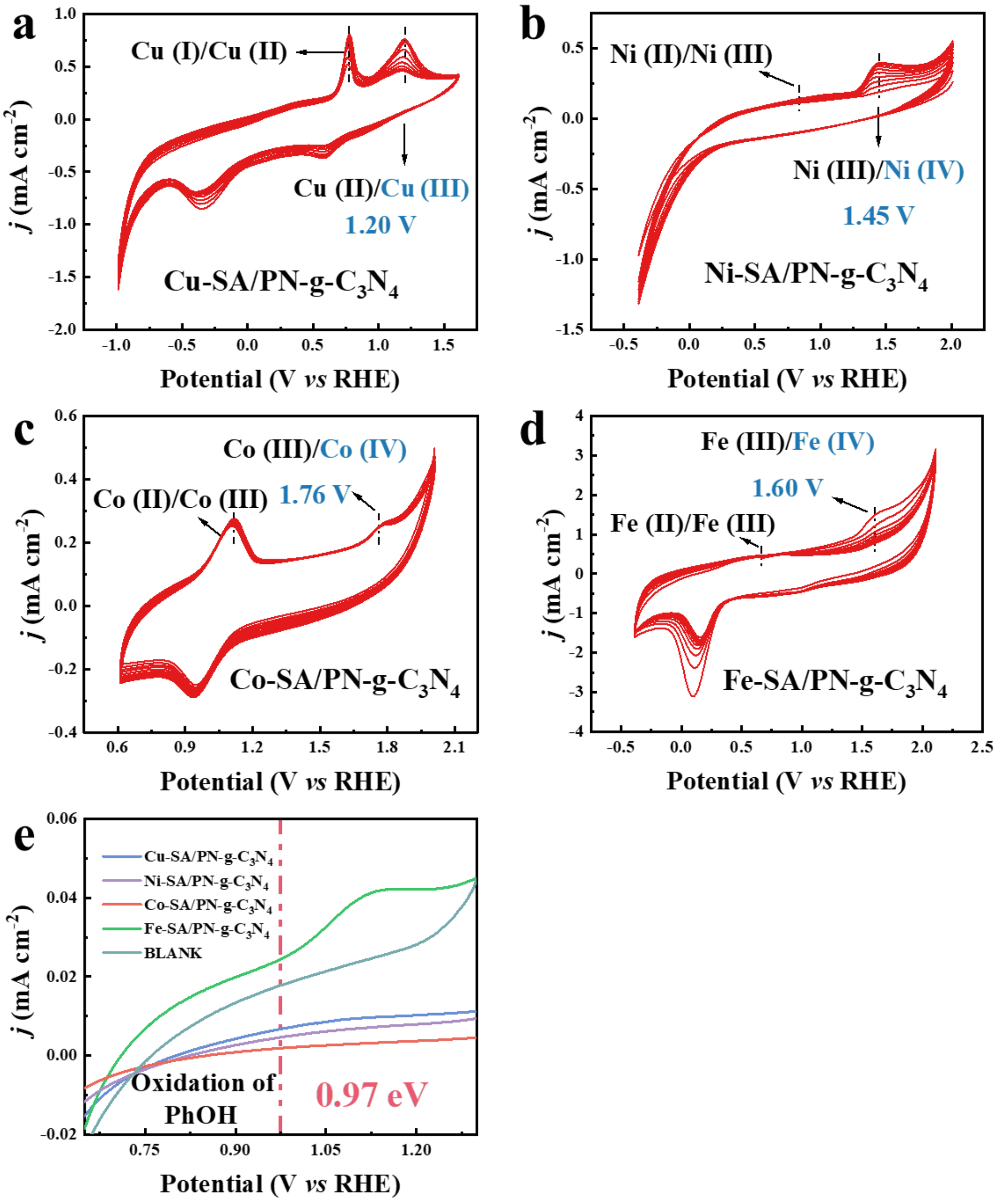

Supplementary Fig. 36 | Electrochemical characterization of catalysts. a-d, CV spectra for PhOH oxidation by Cu **(a)**, Ni **(b)**, Co **(c)**, and Fe **(d)**-SA/PN-g-C_3N_4 system. **e,** LSV curve for PhOH oxidation by the different catalysts.

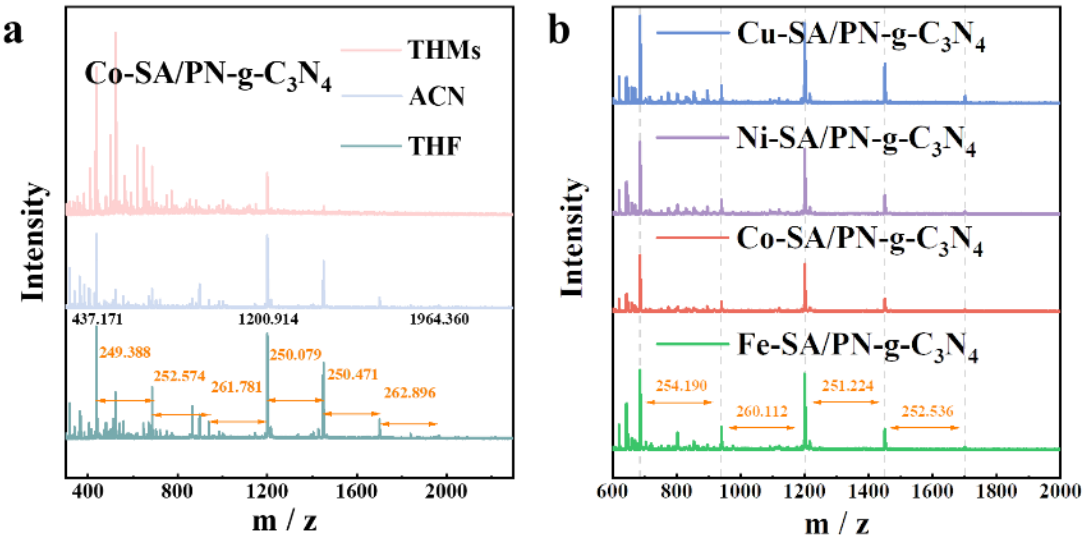

Supplementary Fig. 37 | Polymer elution on catalyst surface. a, MALDI-TOF-MS of the polymerization products on Co-SA/PN-g-C_3N_4 washed by THMs, ACN, THF. **b**, MALDI-TOF mass spectra of the polymerization products on different catalysts washed by THF. Reaction conditions: [Cat.] = 1.0 g L^{-1}, [PMS] = 8.0 mM, [PhOH] = 2.0 mM, initial pH = 7.0, T = 25 ± 2 °C.

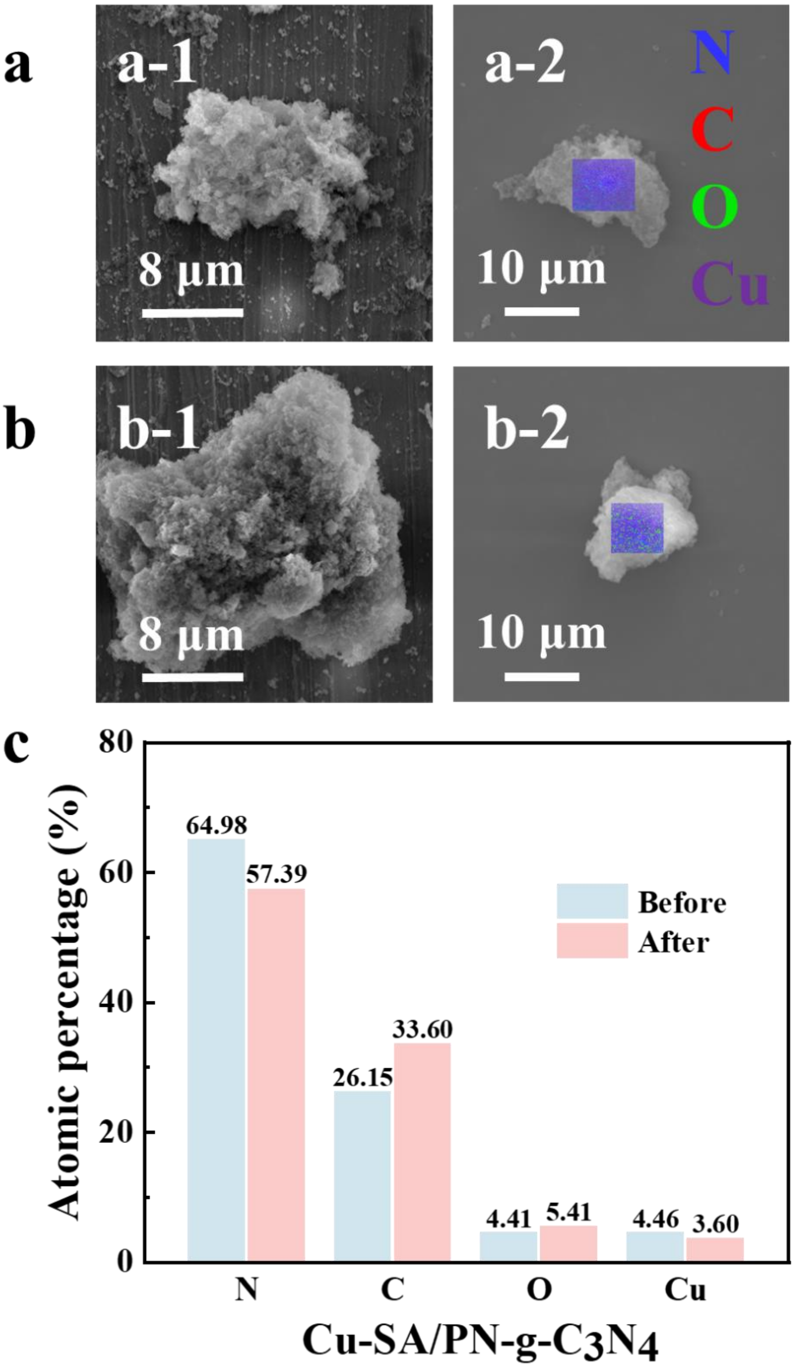

Supplementary Fig. 38 | The changes of SEM morphology and elemental composition of Cu-SA/PN-g-C_3N_4 surface before and after reaction. a, b, SEM images and the Corresponding EDS mappings of Cu-SA/PN-g-C_3N_4 before and after the reaction. **c,** Comparison of EDS content before and after the reaction. Reaction conditions: [Cat.] = 1.0 g L^{-1}, [PMS] = 1.0 mM, [2, 6-M-PhOH] = 0.5 mM, initial pH = 7.0, T = 25 ± 2 °C. Blue, red, green and purple represent N, C, O and Cu, respectively.

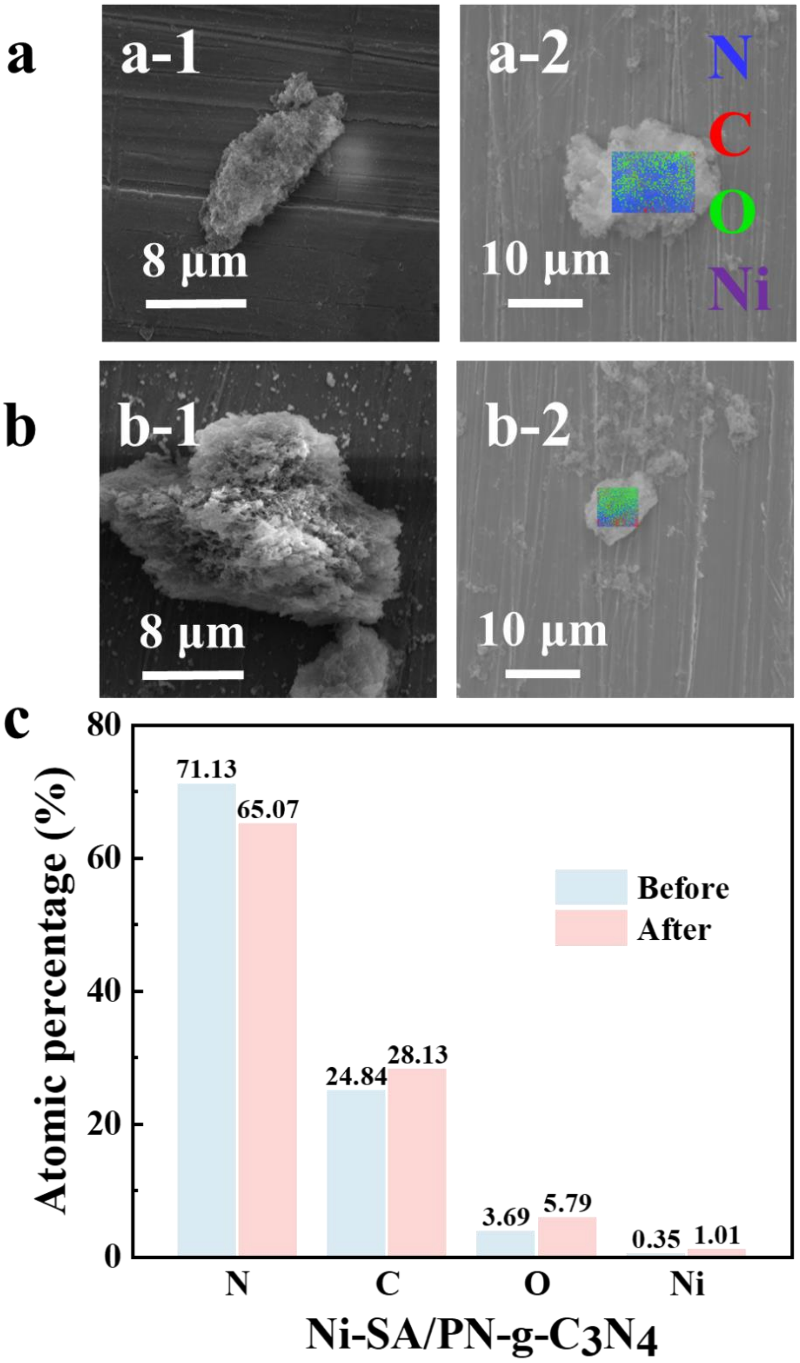

Supplementary Fig. 39 | The changes of SEM morphology and elemental composition of Ni-SA/PN-g-C_3N_4 surface before and after reaction. a, b, SEM images and the Corresponding EDS mappings of Ni-SA/PN-g-C_3N_4 before and after the reaction. **c,** Comparison of EDS content before and after the reaction. Reaction conditions: [Cat.] = 1.0 g L^{-1}, [PMS] = 1.0 mM, [2, 6-M-PhOH] = 0.5 mM, initial pH = 7.0, T = 25 ± 2 °C. Blue, red, green and purple represent N, C, O and Cu, respectively.

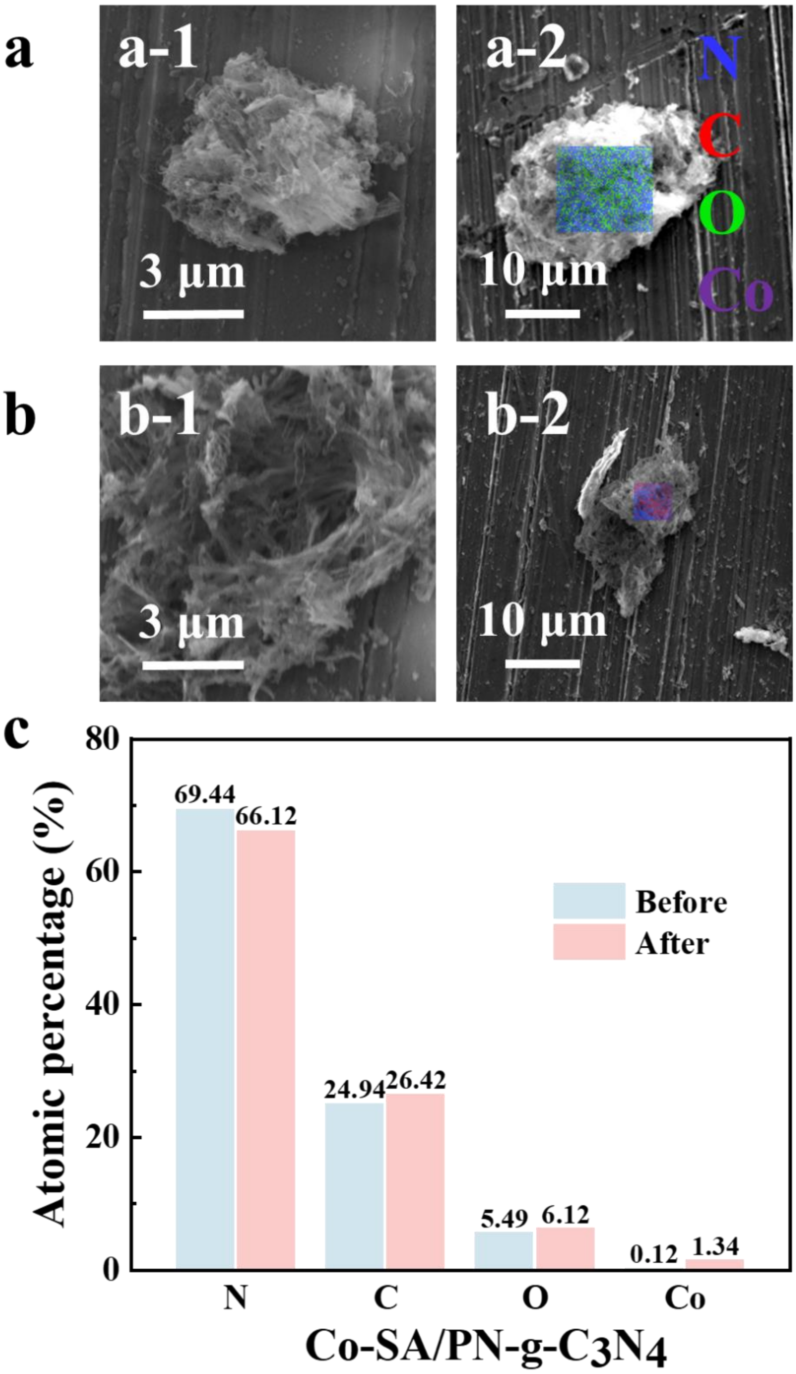

Supplementary Fig. 40 | The changes of SEM morphology and elemental composition of Co-SA/PN-g-C_3N_4 surface before and after reaction. a, b, SEM images and the Corresponding EDS mappings of Co-SA/PN-g-C_3N_4 before and after the reaction. **c,** Comparison of EDS content before and after the reaction. Reaction conditions: [Cat.] = 1.0 g L^{-1}, [PMS] = 1.0 mM, [2, 6-M-PhOH] = 0.5 mM, initial pH = 7.0, T = 25 ± 2 °C. Blue, red, green and purple represent N, C, O and Cu, respectively.

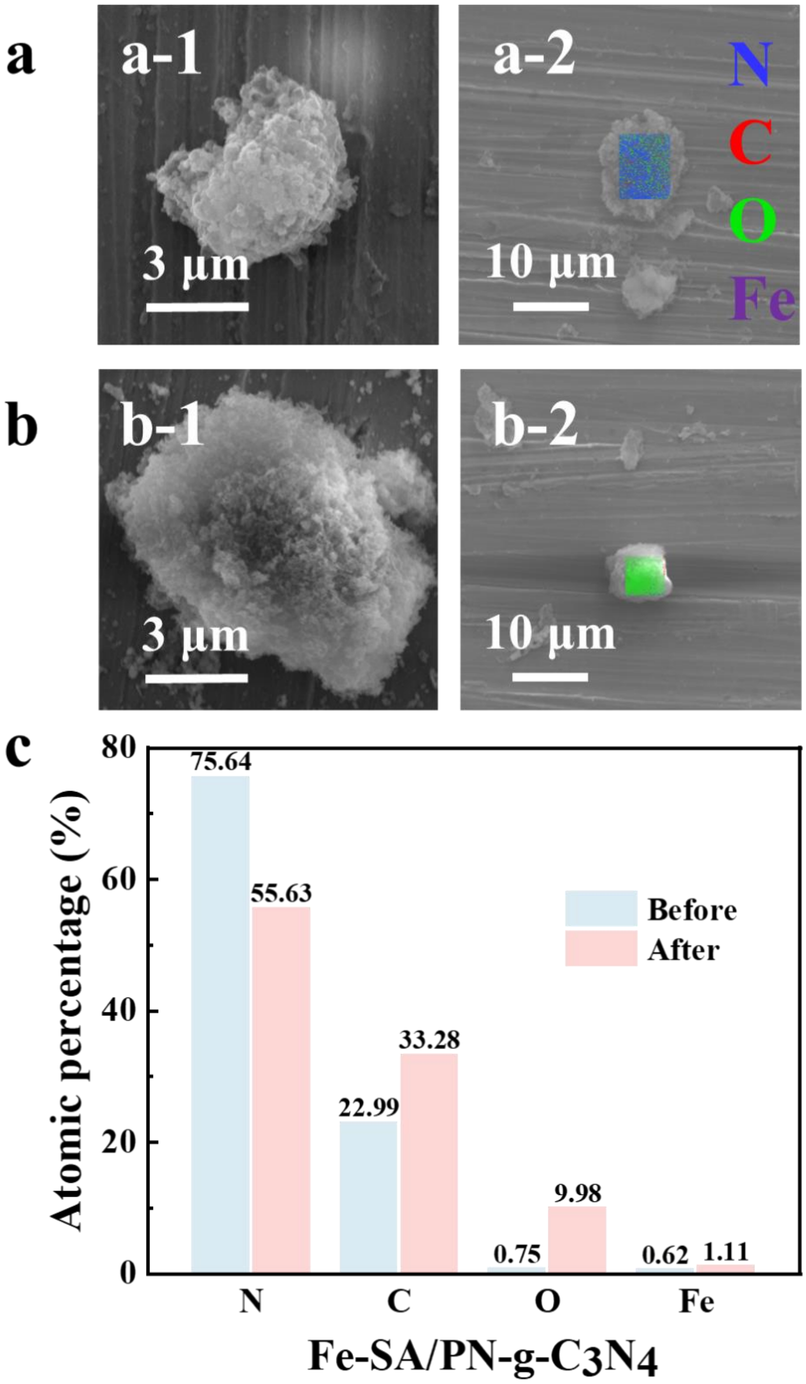

Supplementary Fig. 41 | The changes of SEM morphology and elemental composition of Fe-SA/PN-g-C_3N_4 surface before and after reaction. a, b, SEM images and the Corresponding EDS mappings of Fe-SA/PN-g-C_3N_4 before and after the reaction. **c,** Comparison of EDS content before and after the reaction. Reaction conditions: [Cat.] = 1.0 g L^{-1}, [PMS] = 1.0 mM, [2, 6-M-PhOH] = 0.5 mM, initial pH = 7.0, T = 25 ± 2 °C. Blue, red, green and purple represent N, C, O and Cu, respectively.

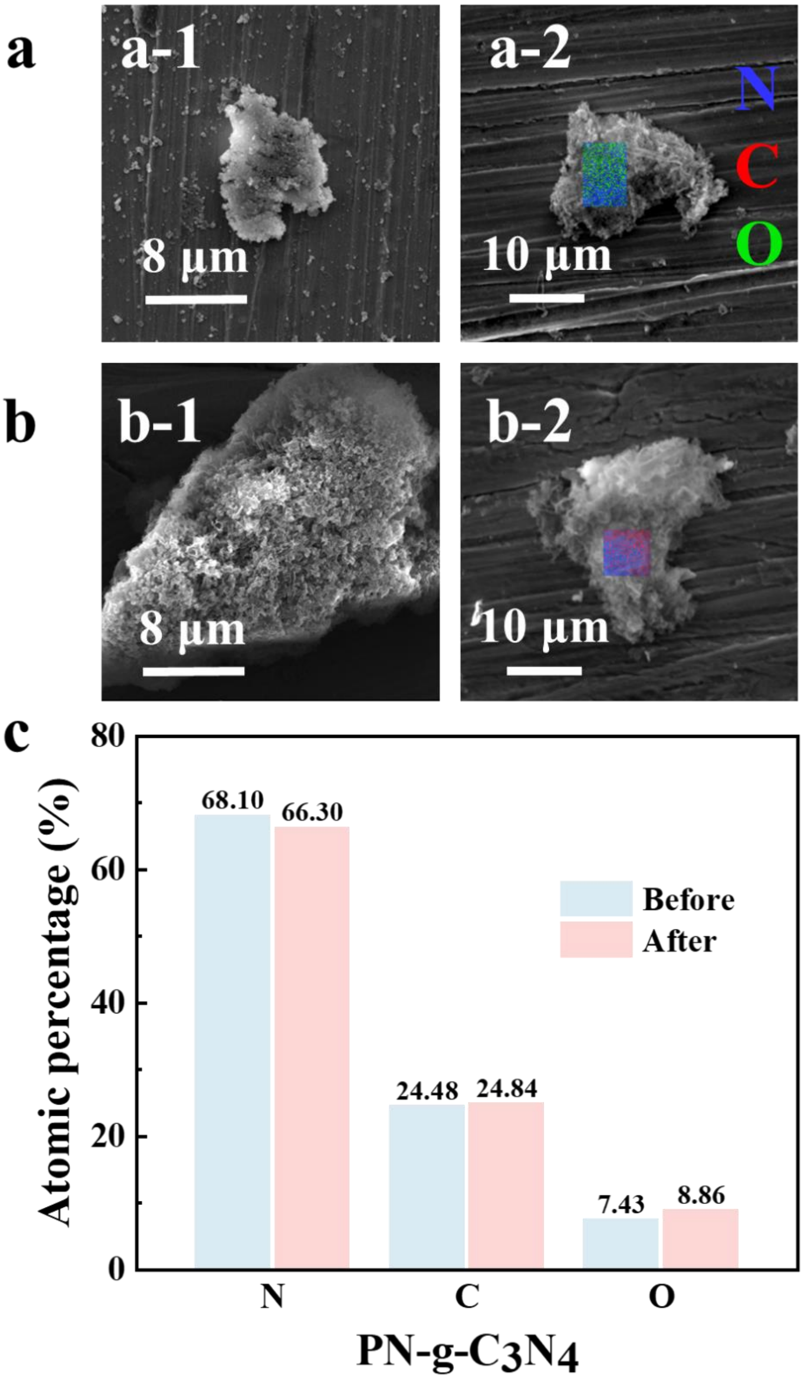

Supplementary Fig. 42 | The changes of SEM morphology and elemental composition of PN-g-C_3N_4 surface before and after reaction. a, b, SEM images and the Corresponding EDS mappings of PN-g-C_3N_4 before and after the reaction. **c,** Comparison of EDS content before and after the reaction. Reaction conditions: [Cat.] = 1.0 g L^{-1}, [PMS] = 1.0 mM, [2, 6-M-PhOH] = 0.5 mM, initial pH = 7.0, T = 25 ± 2 °C. Blue, red, green and purple represent N, C, O and Cu, respectively.

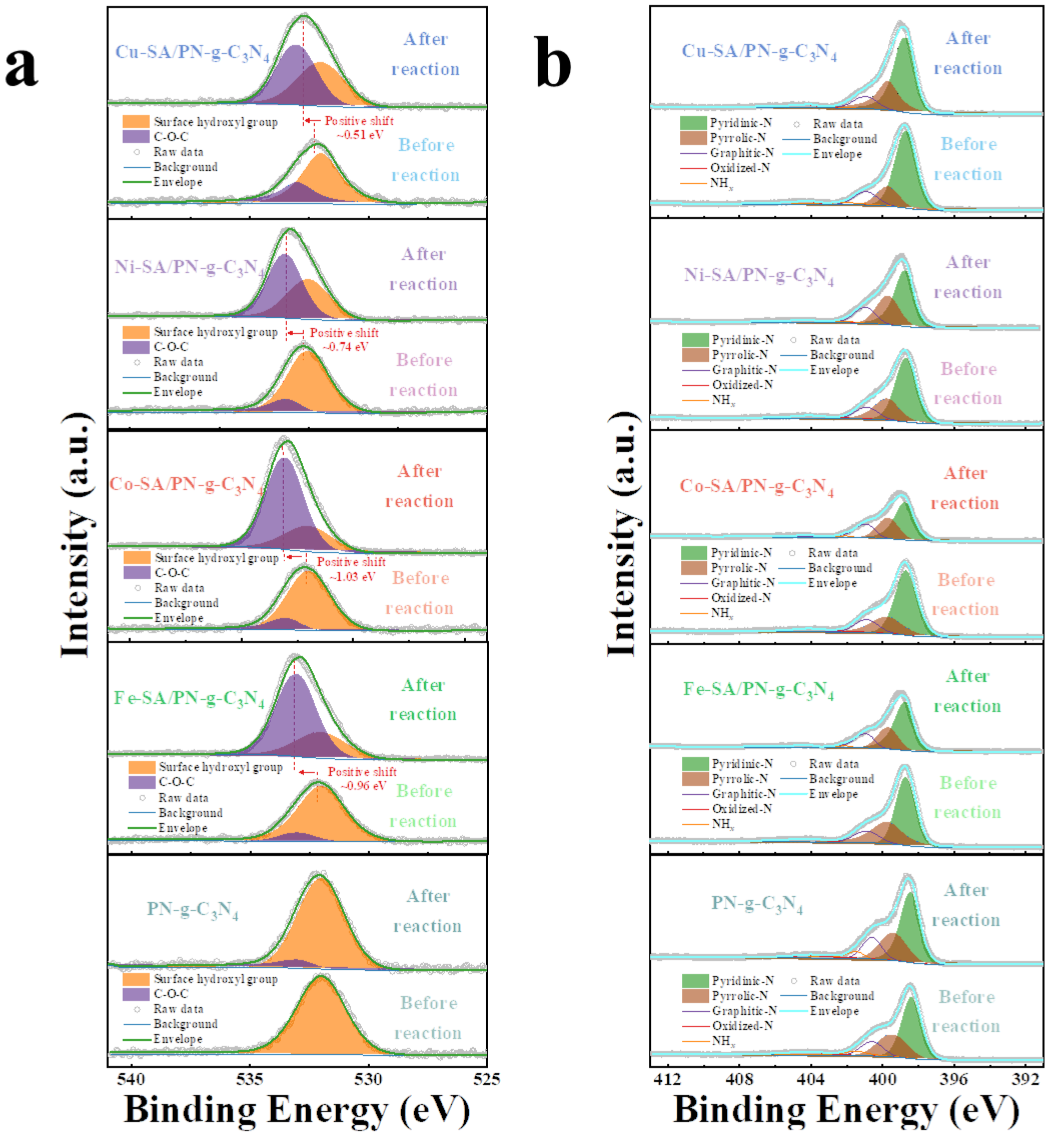

Supplementary Fig. 43 | XPS tracking of catalyst before and after reaction. a, b, O 1*s* **(a)** and N 1*s* **(b)** spectra of XPS before and after 2, 6-M-PhOH degradation reaction for the different TM-SA/PN-g-C_3N_4 catalysts. Reaction conditions: [Cat.] = 1.0 g L^{-1}, [PMS] = 1.0 mM, [2, 6-M-PhOH] = 0.5 mM, initial pH = 7.0, T = 25 ± 2 °C.

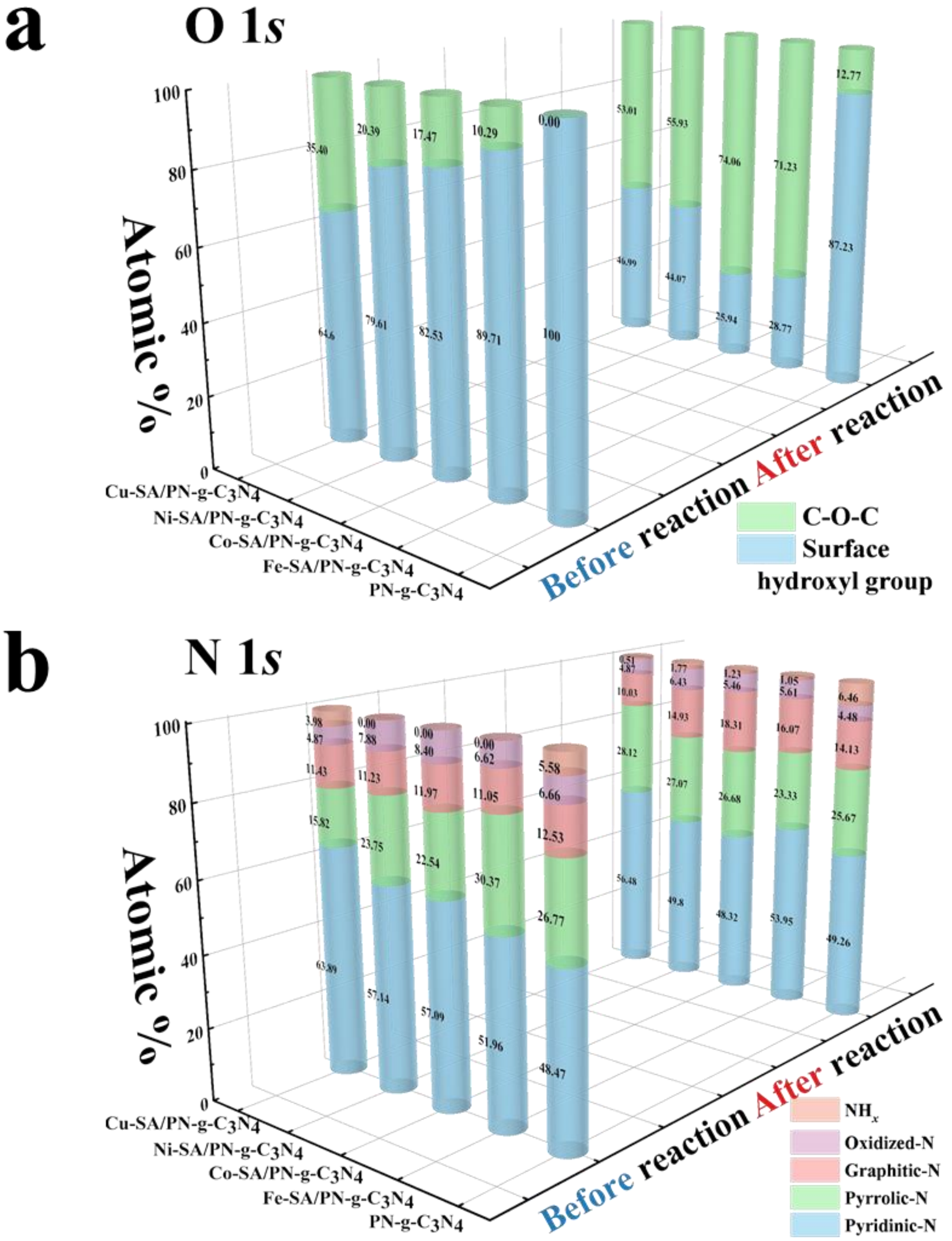

Supplementary Fig. 44 | The proportion of O and N components before and after the reaction was measured by XPS peak fitting. O 1*s* **(a)** and N 1*s* **(b)** component of XPS of the different TM-SA/PN-g-C_3N_4 catalysts before and after 2, 6-M-PhOH degradation reaction. Reaction conditions: [Cat.] = 1.0 g L^{-1}, [PMS] = 1.0 mM, [2, 6-M-PhOH] = 0.5 mM, initial pH = 7.0, T = 25 ± 2 °C.

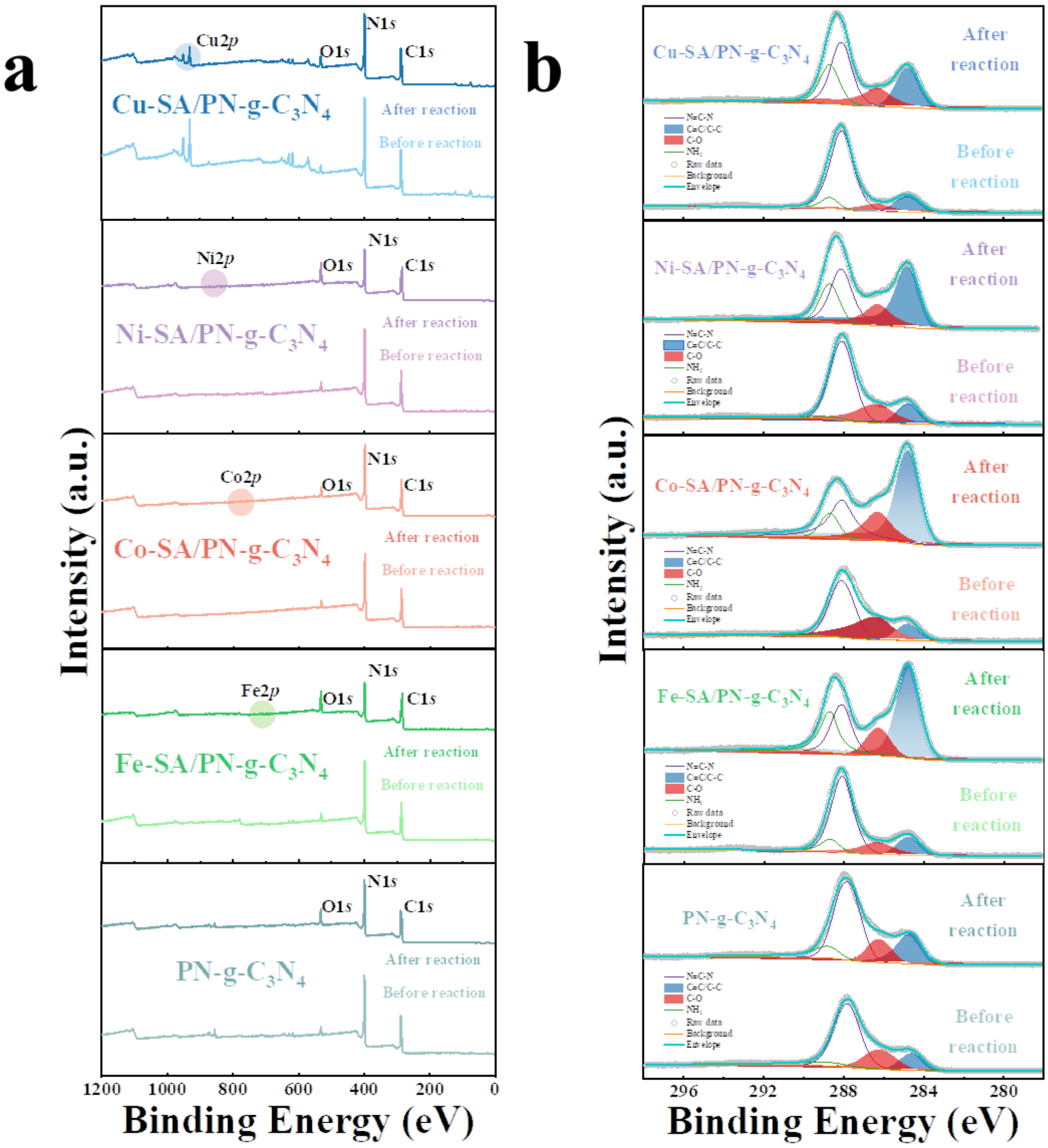

Supplementary Fig. 45 | XPS tracking of catalyst before and after reaction. XPS survey **(a)** and C 1*s* **(b)** spectra of TM-SA/PN-g-C_3N_4 and PN-g-C_3N_4 before and after the 2, 6-M-PhOH degradation reaction. Reaction conditions: [Cat.] = 1.0 g L^{-1}, [PMS] = 1.0 mM, [2, 6-M-PhOH] = 0.5 mM, initial pH = 7.0, T = 25 ± 2 °C.

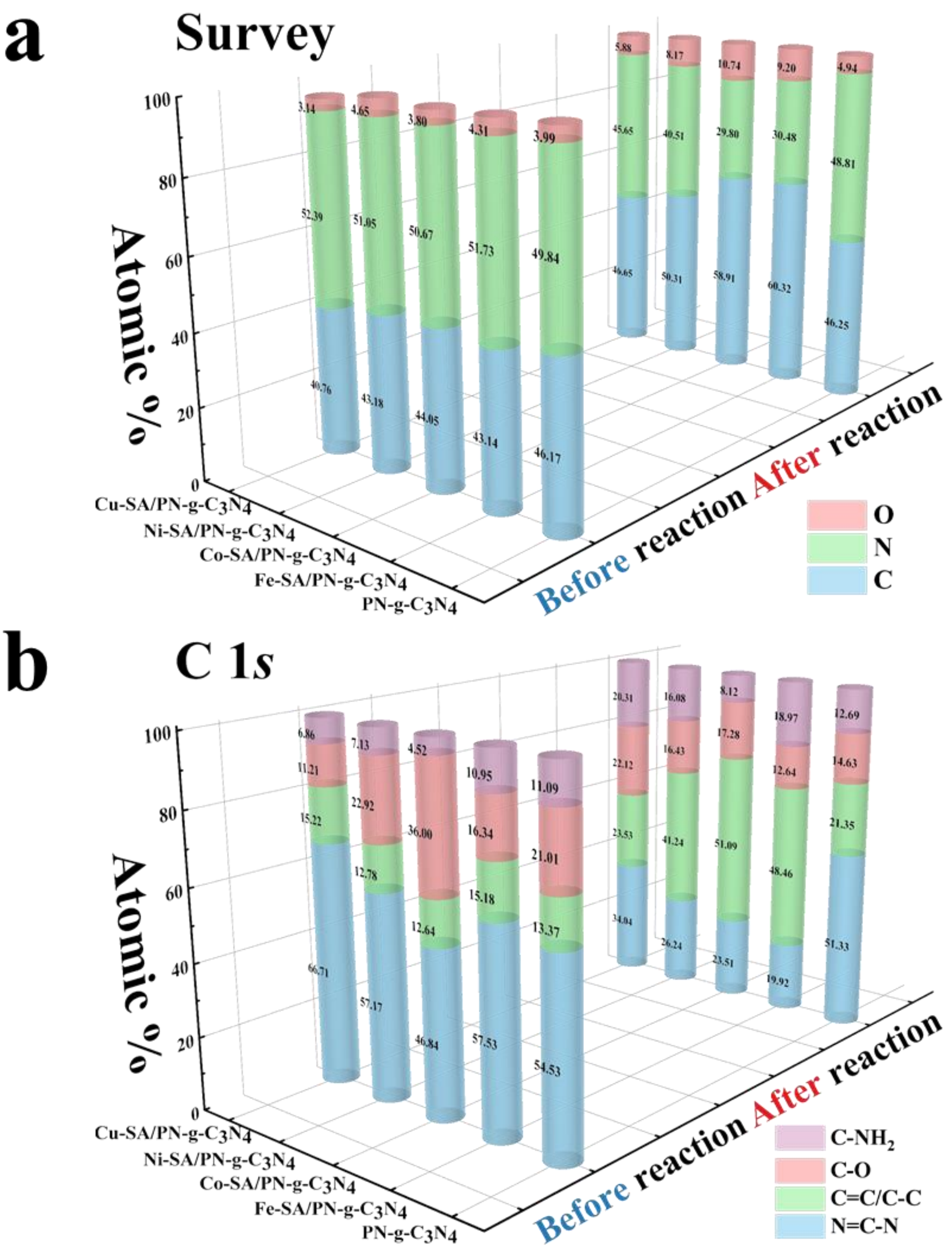

Supplementary Fig. 46 | The proportion of total element and C components before and after the reaction was measured by XPS peak fitting. XPS survey **(a)** and C 1*s* **(b)** components of the catalysts before and after the 2, 6-M-PhOH degradation reaction. Reaction conditions: [Cat.] = 1.0 g L^{-1}, [PMS] = 1.0 mM, [2, 6-M-PhOH] = 0.5 mM, initial pH = 7.0, T = 25 ± 2 °C.

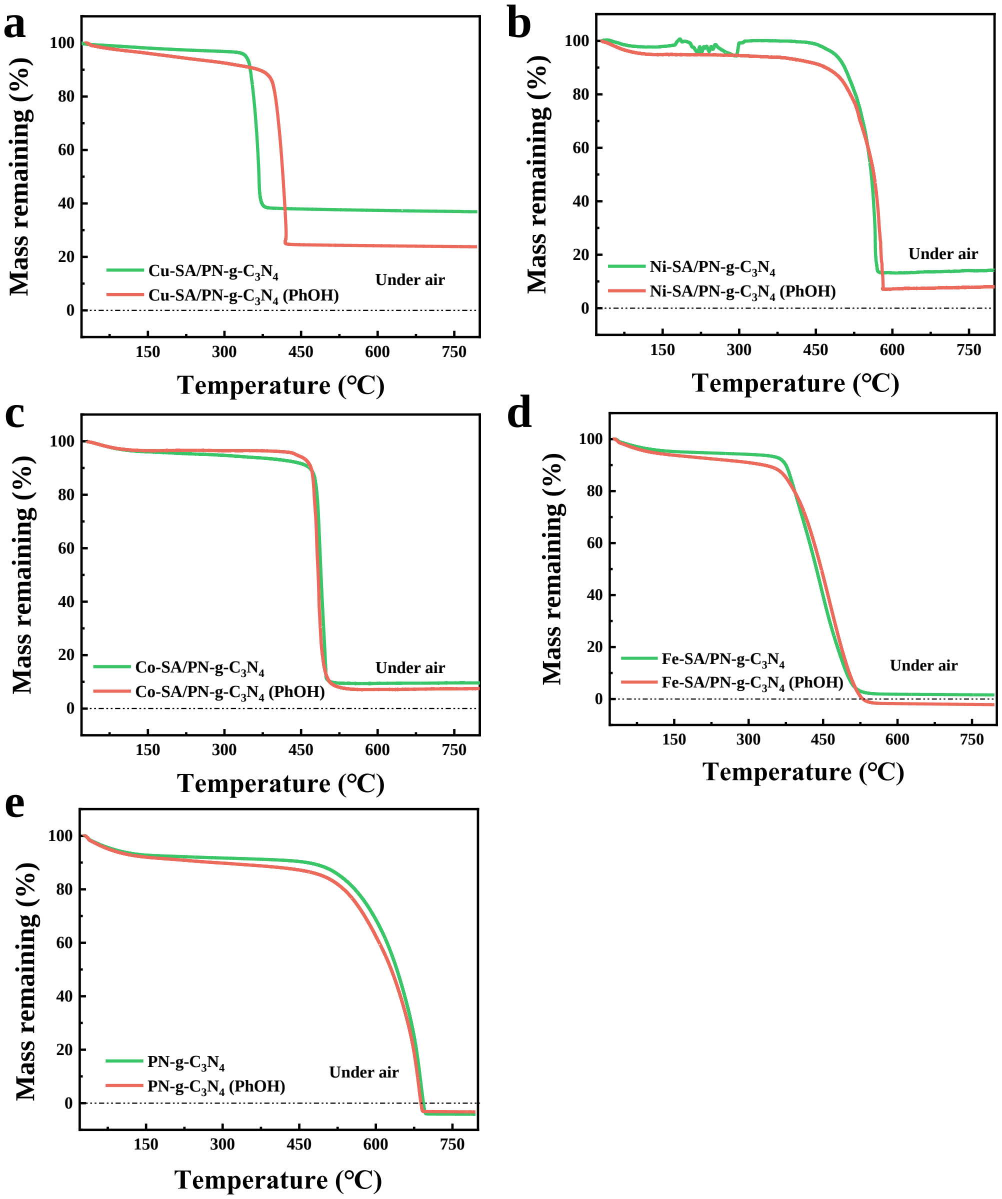

Supplementary Fig. 47 | TGA before and after reaction. a-e, Linear temperature program TGA diagrams of Cu **(a)**, Ni **(b)**, Co **(c),** Fe **(d)** -SA/PN-g-C_3N_4, and PN-g-C_3N_4 **(e)**. Reaction conditions: [Cat.] = 1.0 g L^{-1}, [PMS] = 1.0 mM, [PhOH] = 0.5 mM, initial pH = 7.0, T = 25 ± 2 °C.

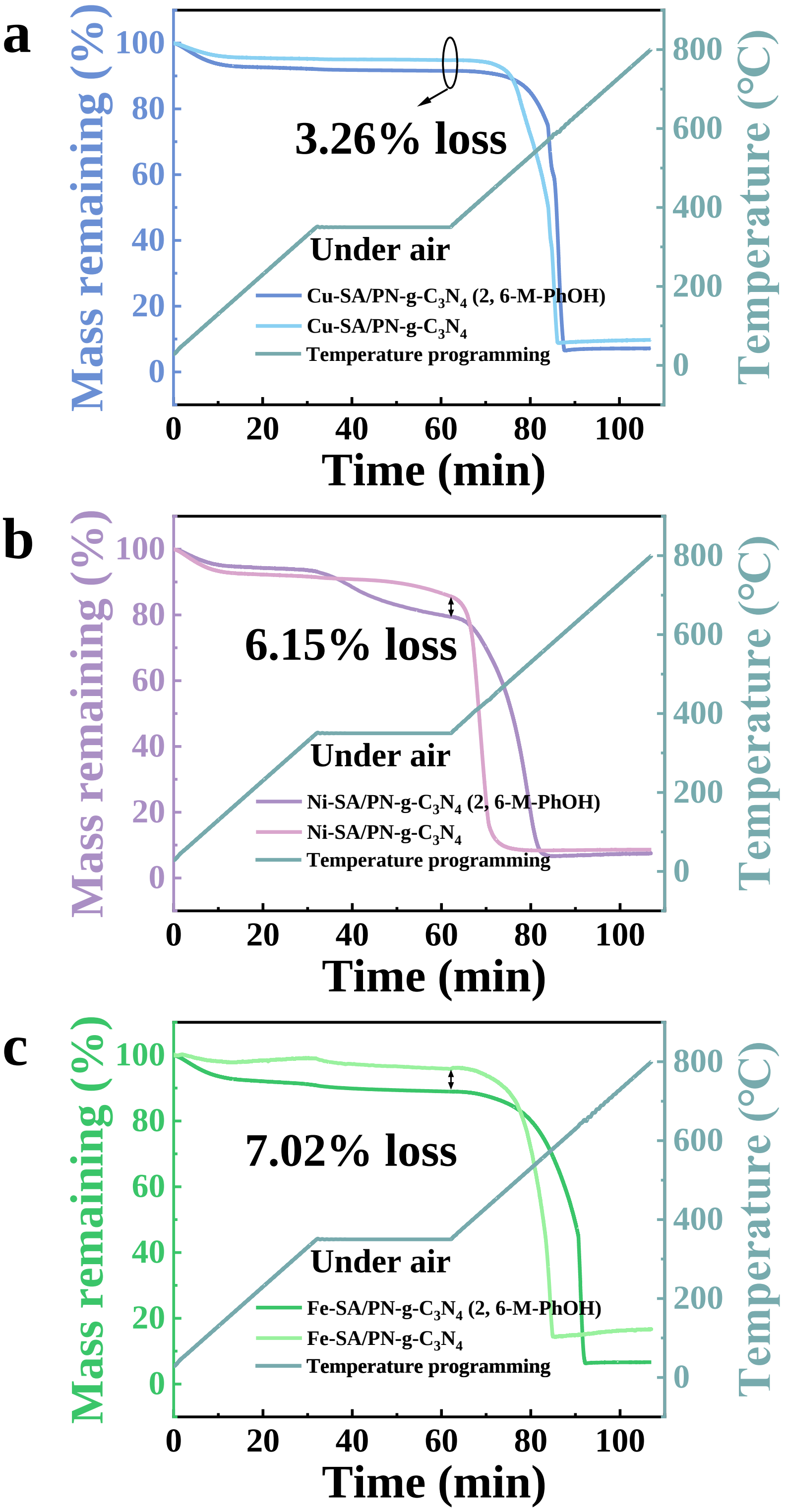

Supplementary Fig. 48 | Reaction before and after the programmed temperature TGA. a-c, Gradient heating TGA diagrams of Cu **(a)**, Ni **(b)**, and Fe **(c)**-SA/PN-g-C_3N_4. Reaction conditions: [Cat.] = 1.0 g L^{-1}, [PMS] = 1.0 mM, [2, 6-M-PhOH] = 0.5 mM, initial pH = 7.0, T = 25 ± 2 °C.

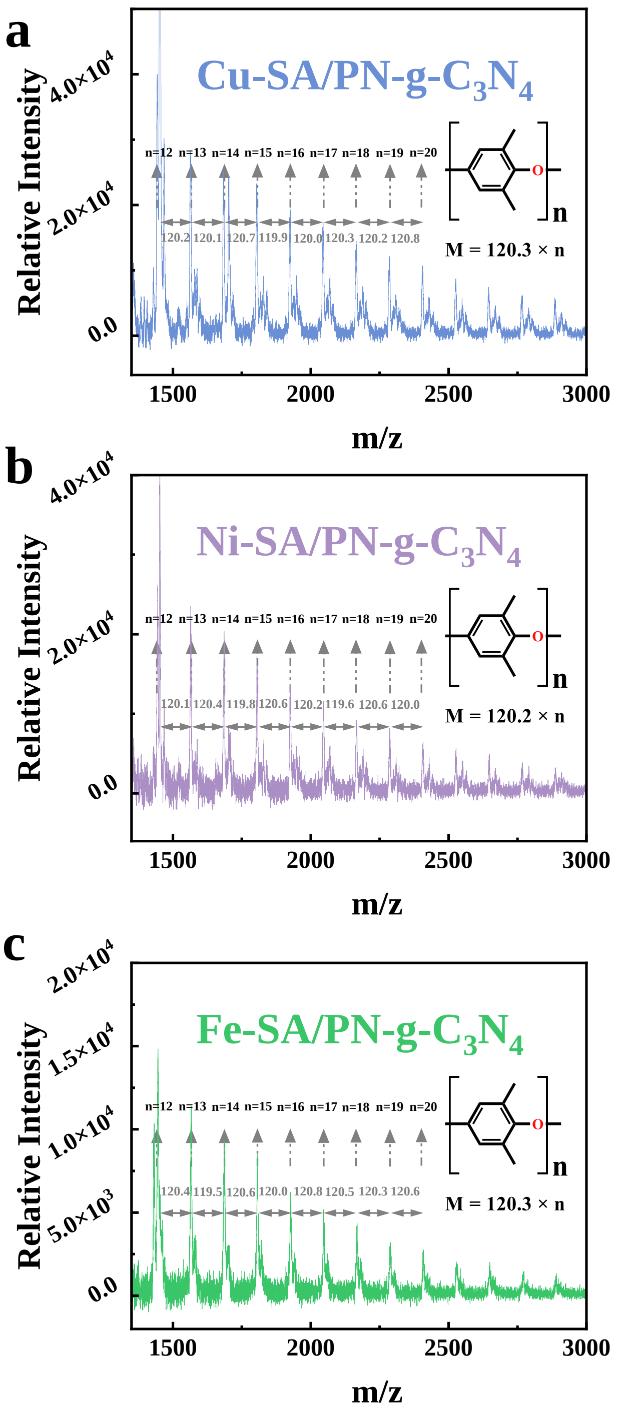

Supplementary Fig. 49 | Elution and identification of polymer on catalyst surface using THF. **a-c**, MALDI-TOF-MS of elution products on the surface of the Cu **(a)**, Ni **(b)**, and Fe **(c)**-SA/PN-g-C_3N_4 catalysts. Reaction conditions: [Cat.] = 1.0 g L^{-1}, [PMS] = 1.0 mM, [2, 6-M-PhOH] = 0.5 mM, initial pH = 7.0, T = 25 ± 2 °C.

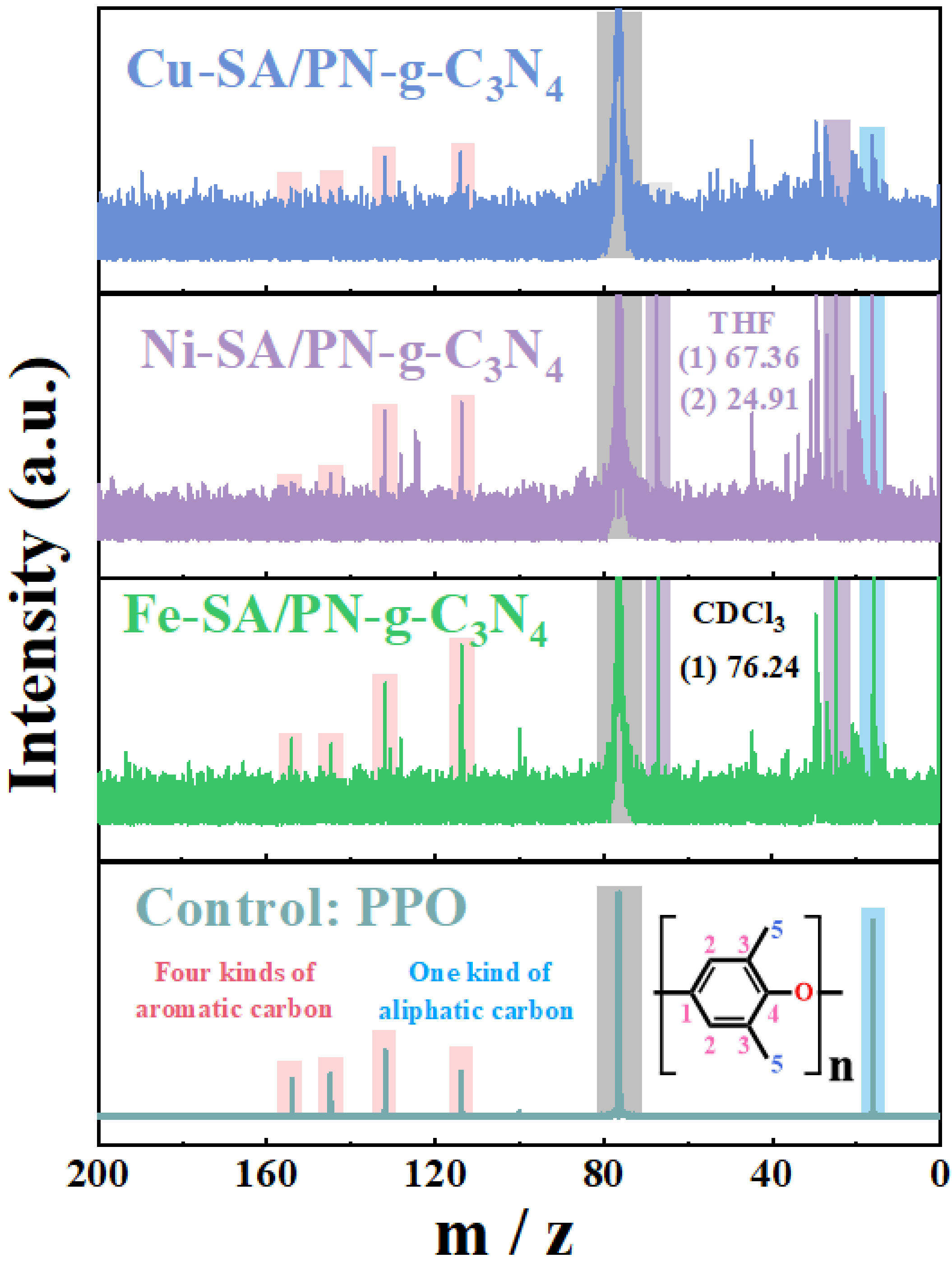

Supplementary Fig. 50 | NMR-based structural analyses of the elution products on the surface of different catalysts. Reaction conditions: [Cat.] = 1.0 g L^{-1}, [PMS] = 1.0 mM, [2, 6-M-PhOH] = 0.5 mM, initial pH = 7.0, T = 25 ± 2 °C. The pink, blue, grey, and purple shades represent four kinds of aromatic carbon, one kind of aliphatic carbon, $CDCl_3$, and, THF respectively.

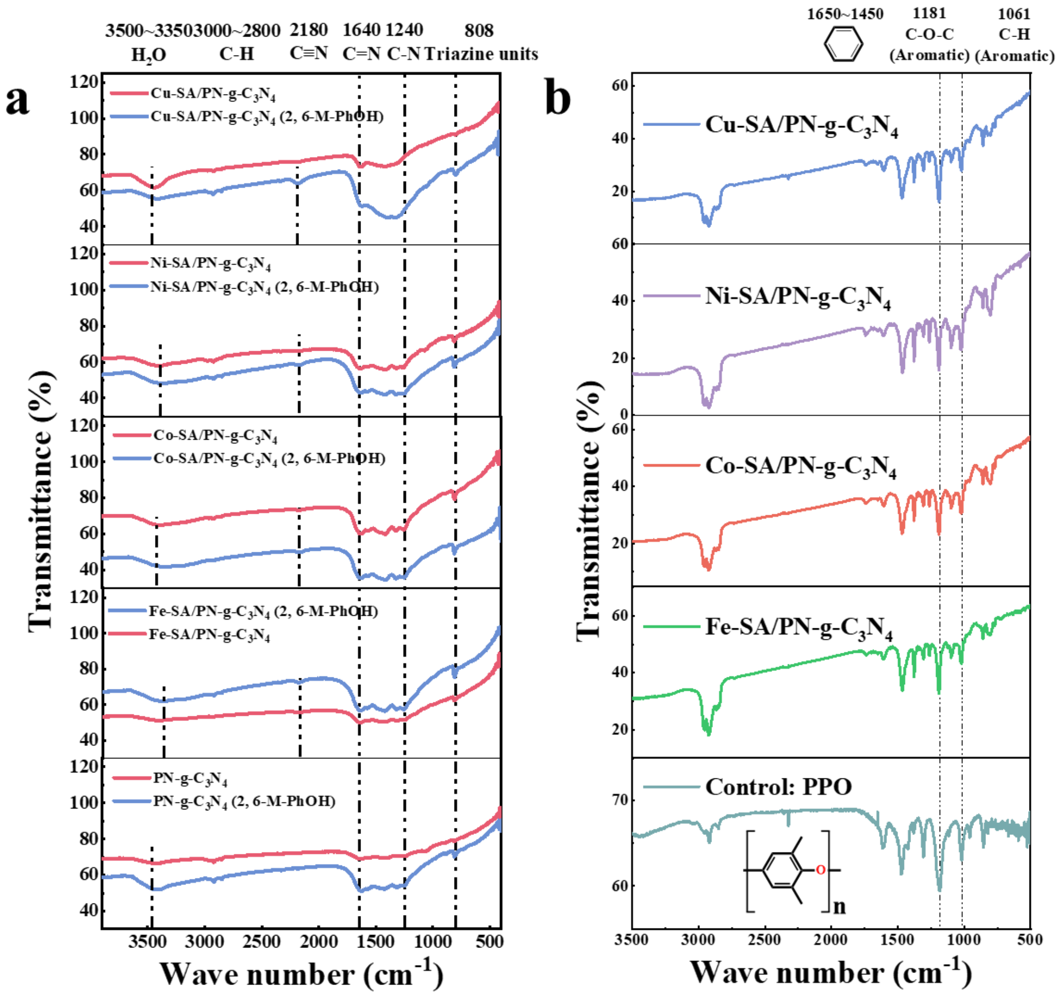

Supplementary Fig. 51 | FTIR spectral analysis. FTIR spectra analyses of the catalyst before and after the 2, 6-M-PhOH degradation reaction, and the elution products on the surface of different catalysts. Reaction conditions: [Cat.] = 1.0 g L^{-1}, [PMS] = 1.0 mM, [2, 6-M-PhOH] = 0.5 mM, initial pH = 7.0, T = 25 ± 2 °C.

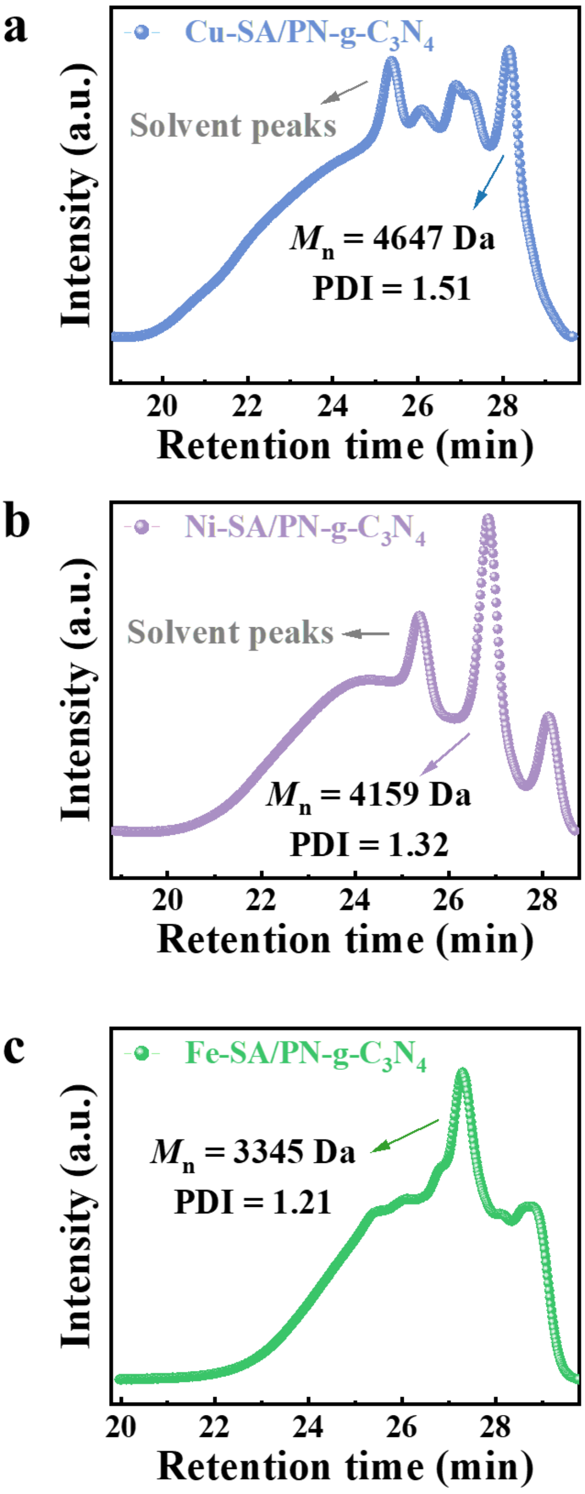

Supplementary Fig. 52 | GPC analysis of elution products. GPC analyses of the surface elution products on Cu **(a)**, Ni **(b)**, and Fe **(c)**-SA/PN-g-C_3N_4. Reaction conditions: [Cat.] = 1.0 g L^{-1}, [PMS] = 1.0 mM, [2, 6-M-PhOH] = 0.5 mM, initial pH = 7.0, T = 25 ± 2 °C.

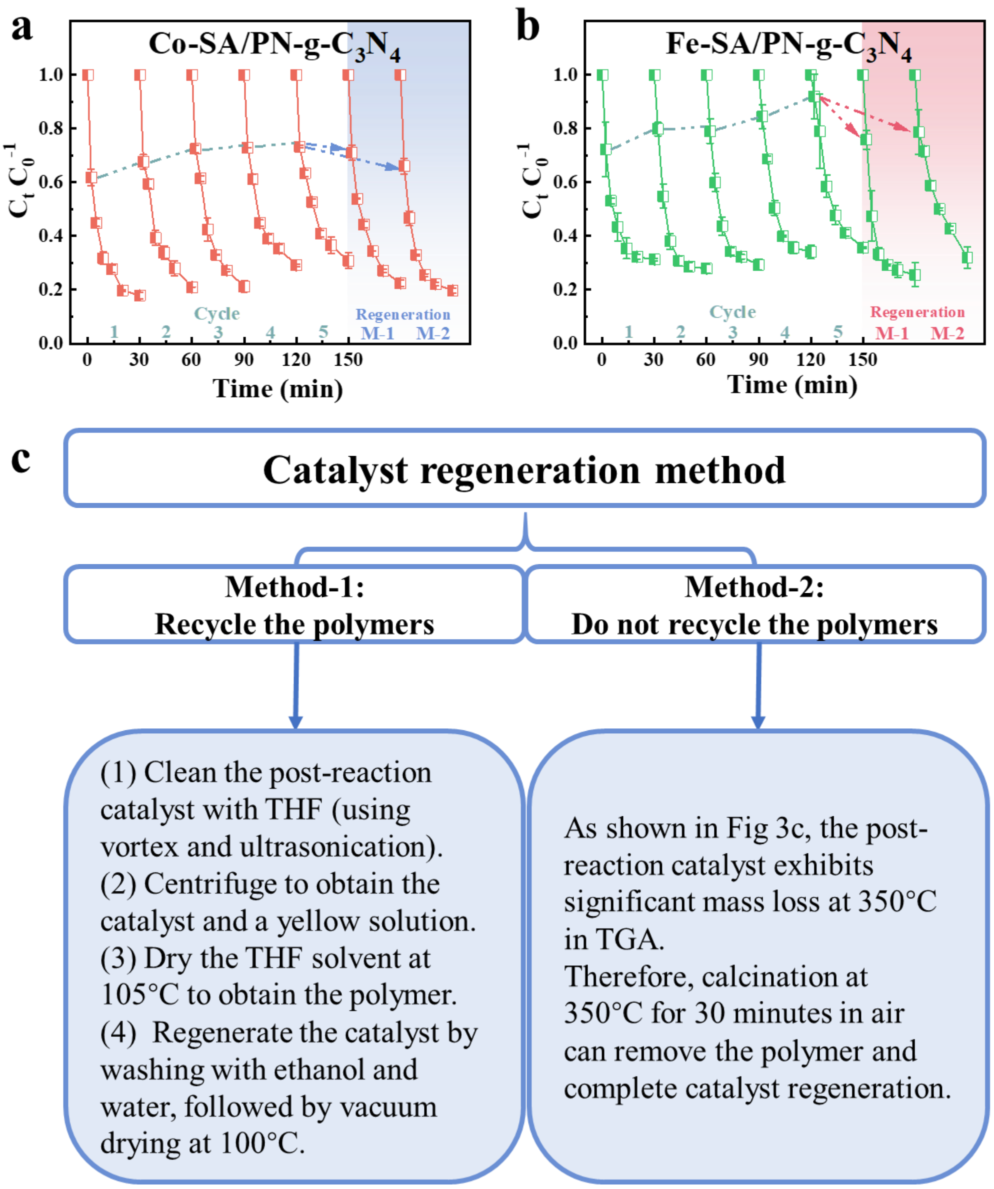

Supplementary Fig. 53 | Experimental procedures and conditions for the cycling reuse and regeneration of the catalysts. a-b, Cycling reuse and regeneration experiments of TM (Co and Fe)-SA/PN-g-C_3N_4. The shading parts represent the regeneration stages. Reaction conditions: [Cat.] = 1.0 g L^{-1}, [PMS] = 1.0 mM, [2, 6-M-PhOH] = 0.5 mM, initial pH = 7.0, T = 25 ± 2 °C. Error bars represent the standard deviation, obtained by repeating the experiment two times.

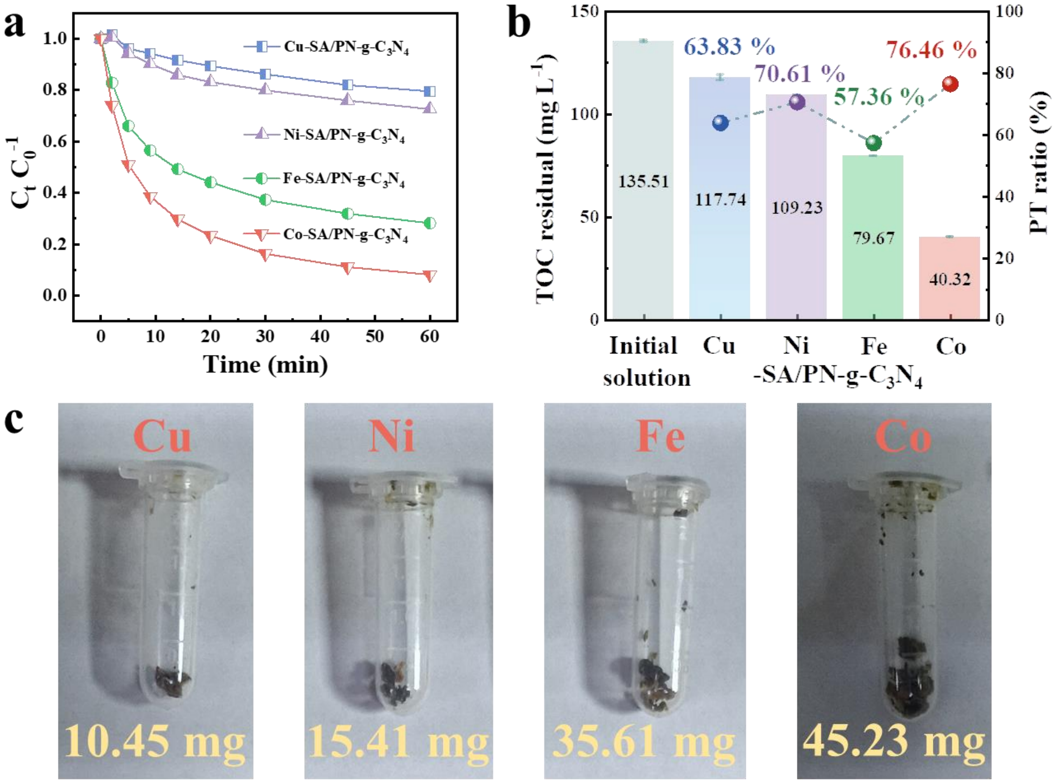

d

TM	Cu	Ni	Fe	Co
2, 6-M-PhOH removal efficiency (%)	20.54	27.47	71.84	91.87
Removed 2, 6-M-PhOH (mg)	5.02	17.55	6.71	22.44
PT ratio (%)	63.83	70.60	57.36	76.46
Surface polymers (mg)	3.20	4.74	10.07	17.16
×4 experiments	12.81	18.95	40.27	68.65
Recoverd polymers (mg)	10.45	15.41	35.61	45.23
Recovery ratio (%)	81.57	81.30	88.43	65.88

Supplementary Fig. 54 | Recovery of the polymers on the catalyst surface. a, Degradation kinetics of 2, 6-M-PhOH in the TM-SA/PN-g-C_3N_4/PMS systems. b, The corresponding TOC residuals and PT ratio values. c, The images of the collected polymers. d, Calculation of the recovery ratios. Reaction conditions: [Cat.] = 1.0 g L^{-1}, [PMS] = 4.0 mM, [2, 6-M-PhOH] = 2.0 mM, initial pH = 7.0, T = 25 ± 2 °C. Error bars represent the standard deviation, obtained by repeating the experiment two times.

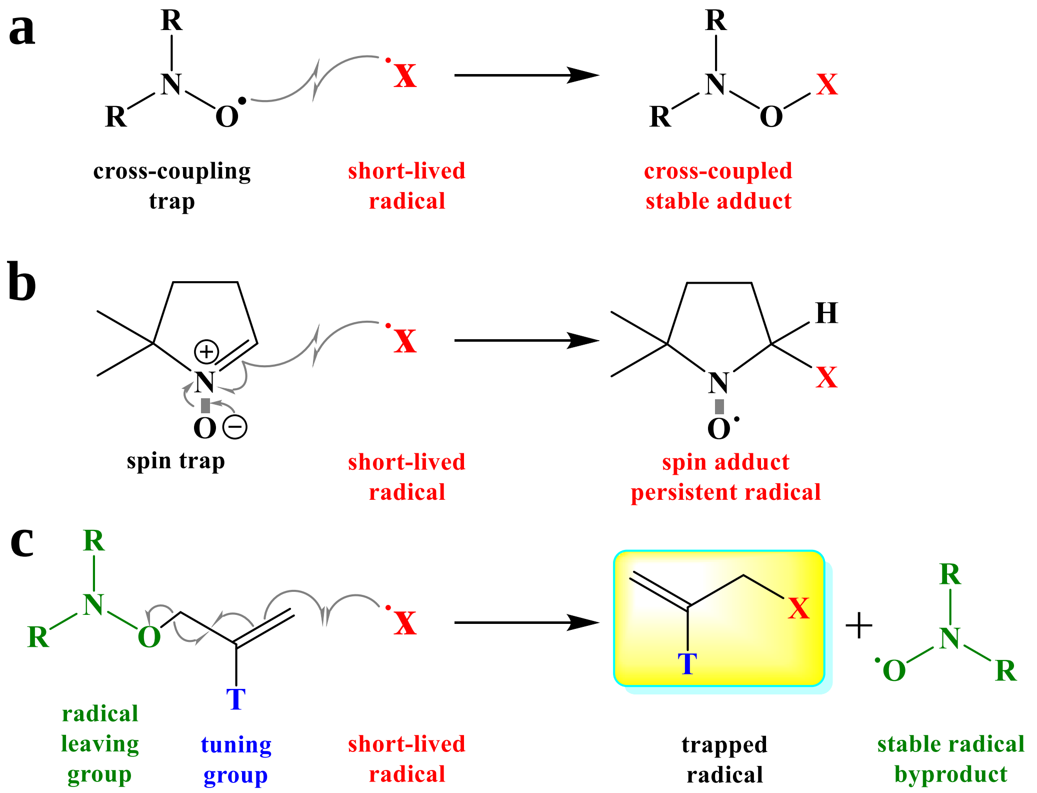

Supplementary Fig. 55 | Capture diagram of organic radical intermediates. The diagrams for cross-coupling trapping **(a)**, spin trapping **(b)**, and the novel radical trap design and the trapping mechanism **(c)**.

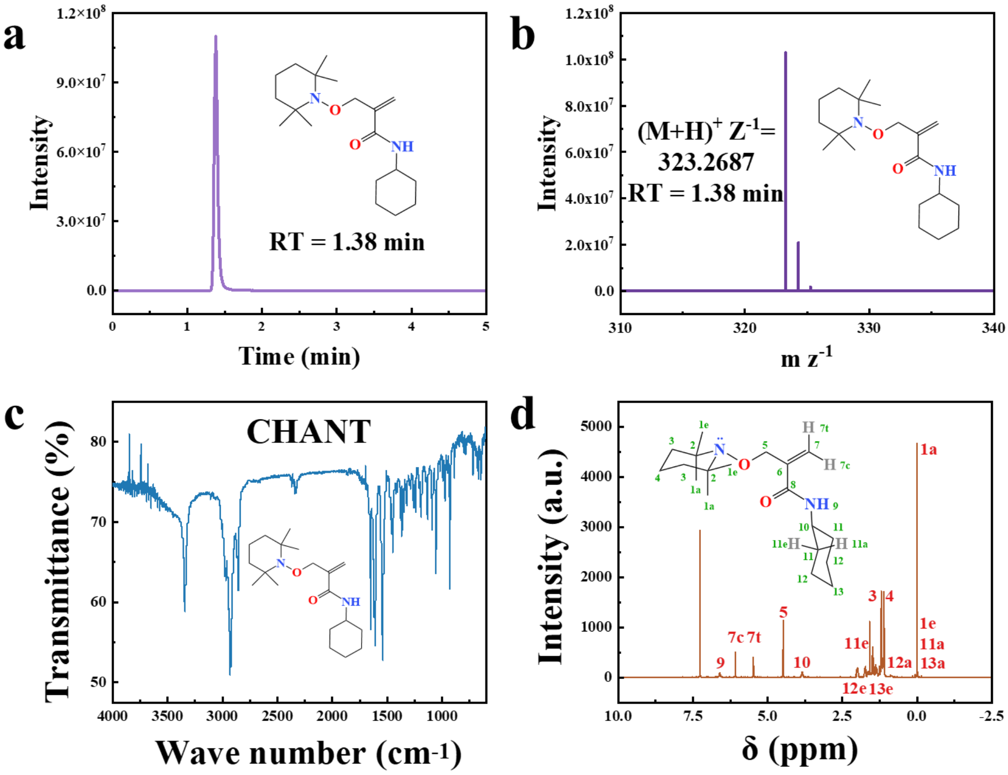

Supplementary Fig. 56 | Synthesis and characterization of CHANT. The chromatogram **(a)**, MS **(b)**, FTIR **(c)**, and NMR **(d)** characterizations of CHANT.

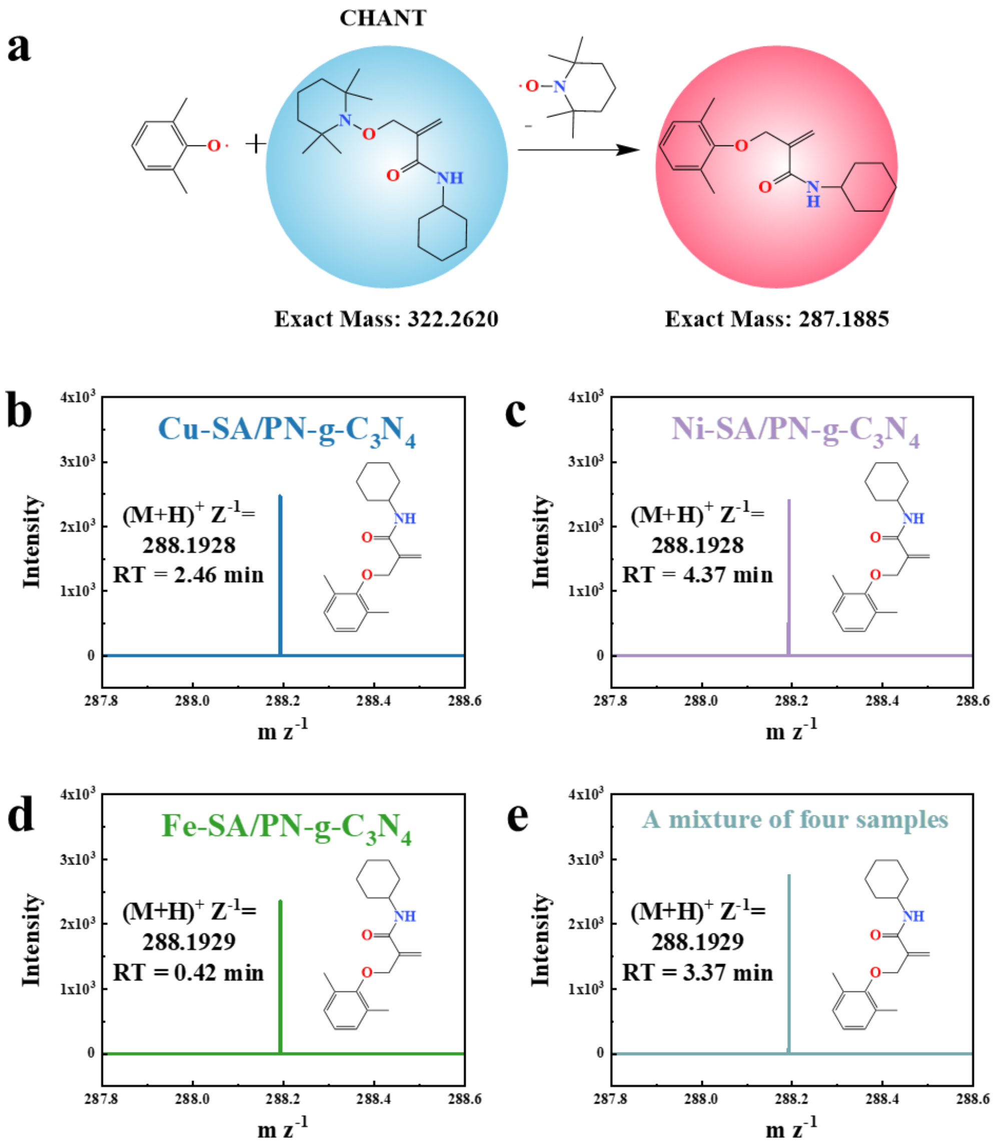

Supplementary Fig. 57 | The mechanism of CHANT trapping phenoxyl radicals and identification of liquid-phase products. Schematic diagram of the capture mechanism of phenoxy radicals by CHANT **(a)**, the trapping results of phenoxy radicals in the Cu **(b)**, Ni **(c)**, Fe **(d)** -SA/PN-g-C_3N_4, and a mixture of four samples **(e)** system. Reaction conditions: [Cat.] = 5.0 g L^{-1}, [PMS] = 10 mM, [2, 6-M-PhOH] = 5 mM, [CHANT] = 1 g L^{-1}, initial pH = 7.0, T = 25 ± 2 °C.

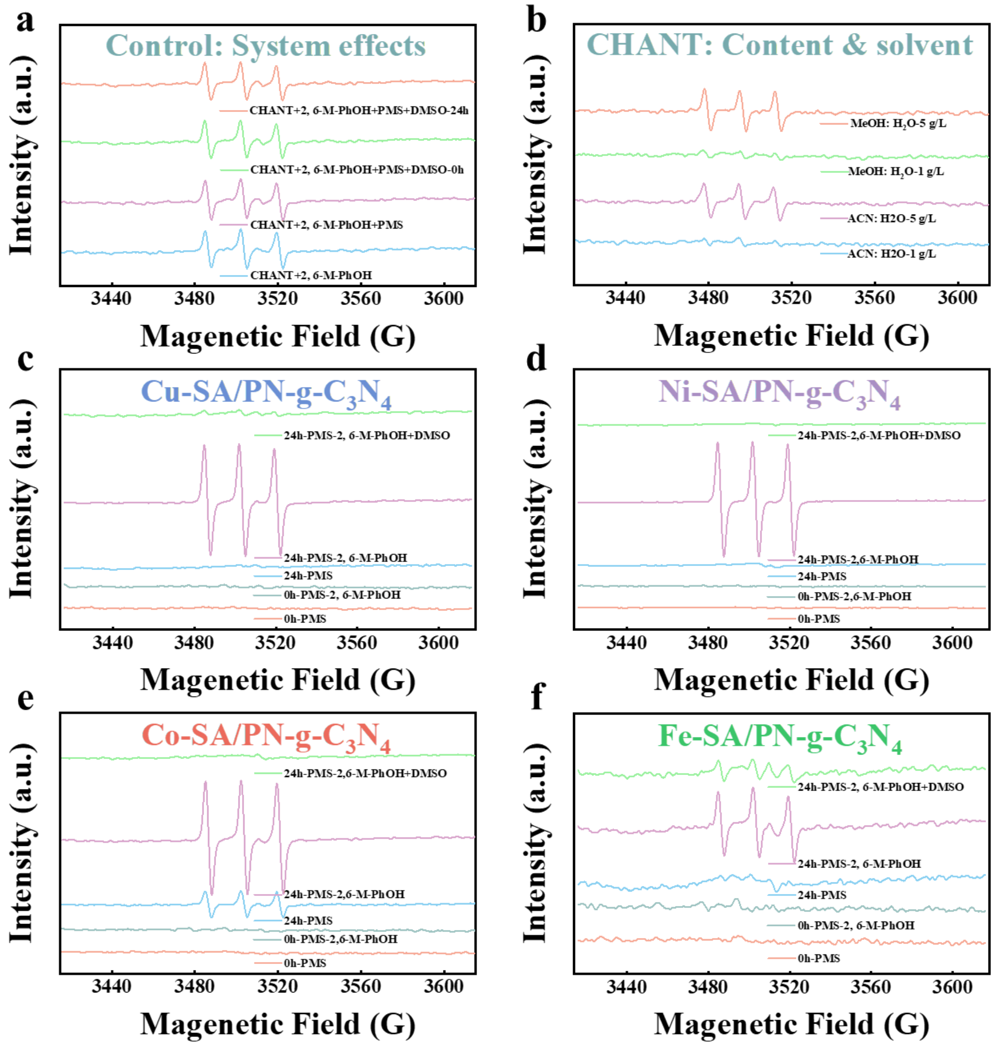

Supplementary Fig. 58 | The accompanying TEMPO release monitoring during CHANT trapping phenoxyl radicals. a-f, EPR spectra for phenoxy radicals trapping by CHANT. Control experiment **(a)** and neutralize CHANT signals with different amounts in different solvents **(b)**. The trapping results in the Cu **(c)**, Ni **(d)**, Co **(e)**, and Fe **(f)**-SA/PN-g-C_3N_4 systems. Reaction conditions: [Cat.] = 5.0 g/L, [PMS] = 10 mM, [2, 6-M-PhOH] = 5 mM, [CHANT] = 1 g L^{-1}, [DMSO] = 10 mM, initial pH = 7.0, T = 25 ± 2 °C.

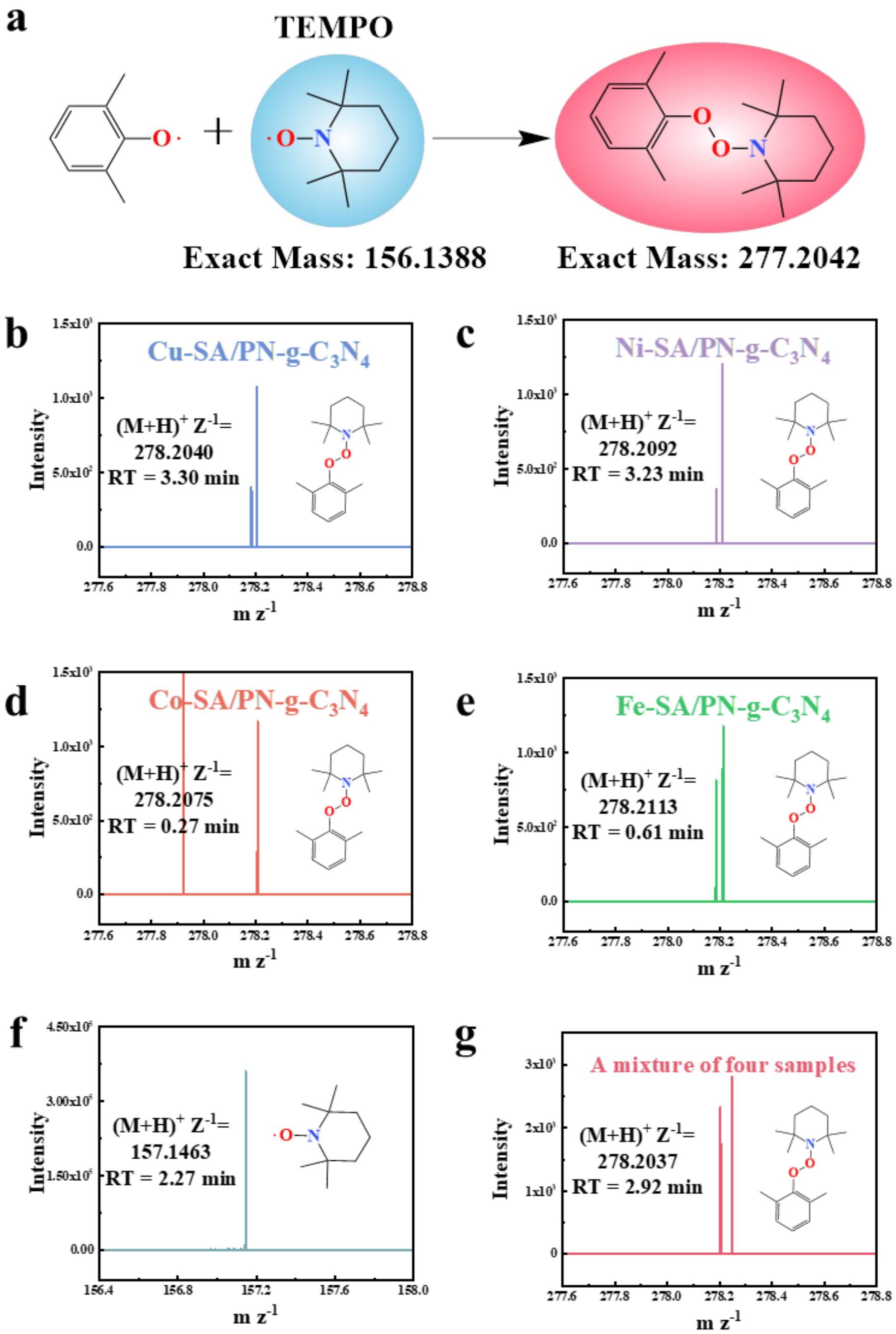

Supplementary Fig. 59 | The mechanism of TEMPO trapping phenoxyl radicals and identification of liquid-phase products. a-g, Trapping of phenoxy radicals by TEMPO cross-coupling reaction. Schematic diagram of reaction mechanism **(a)**. The trapping results in the Cu **(b)**, Ni **(c)**, Co **(d)**, and Fe **(e)**-SA/PN-g-C_3N_4 systems. TEMPO **(f)**, and a mixture of four samples **(g)**. Reaction conditions: [Cat.] = 5.0 g L^{-1}, [PMS] = 10 mM, [2, 6-M-PhOH] = 5 mM, [TEMPO] = 1.0 g L^{-1}, initial pH = 7.0, T = 25 ± 2 °C.

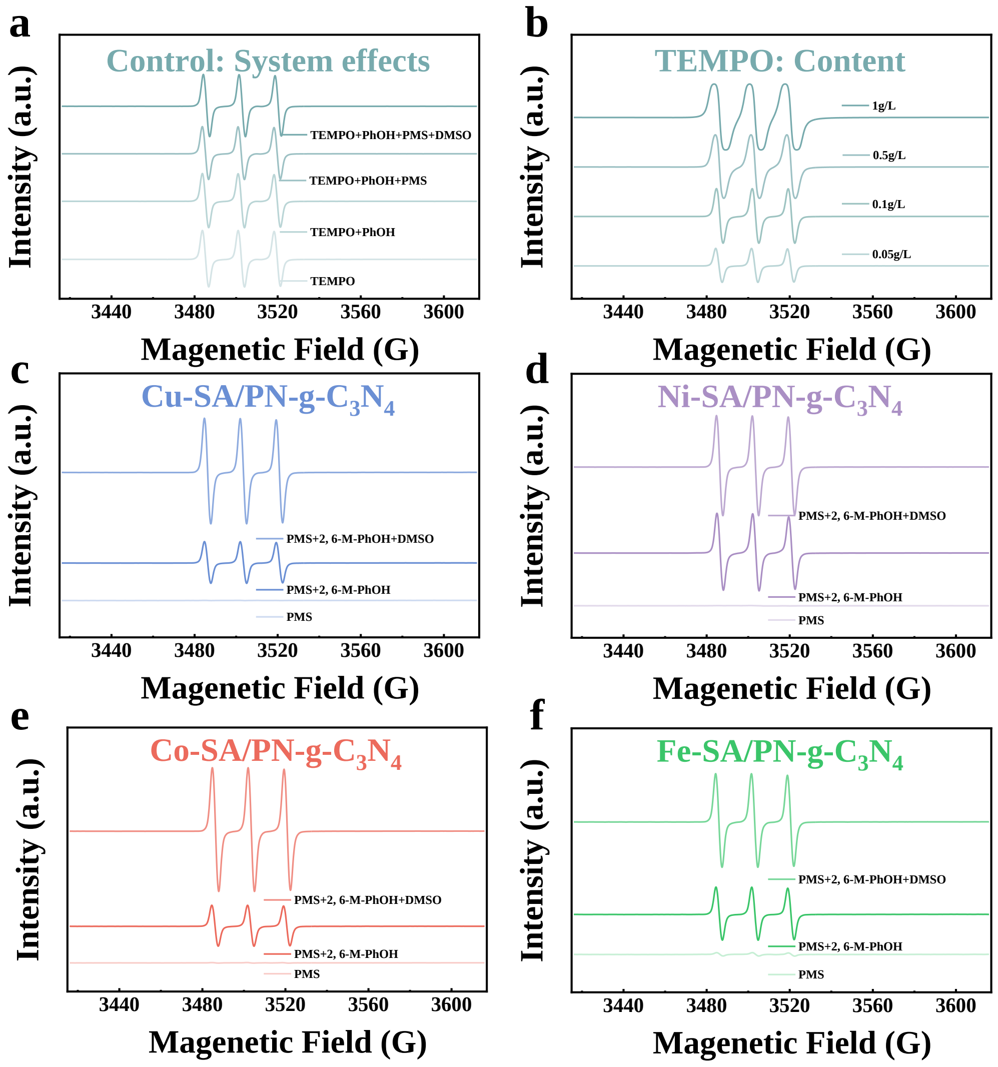

Supplementary Fig. 60 | Changes in EPR signal associated with the capture of phenoxyl radicals by TEMPO. a-f, EPR spectra of TEMPO cross-coupling for phenoxy radical capture. Control experiment **(a)** and TEMPO signals at different dosages of catalysts **(b)**. The trapping results in Cu **(c)**, Ni **(d)**, Co **(e)**, and Fe **(f)**-SA/PN-g-C_3N_4 systems. Reaction conditions: [Cat.] = 5.0 g L^{-1}, [PMS] = 10 mM, [2, 6-M-PhOH] = 5 mM, [TEMPO] = 1.0 g L^{-1}, [DMSO] = 10 mM, initial pH = 7.0, T = 25 ± 2 °C.

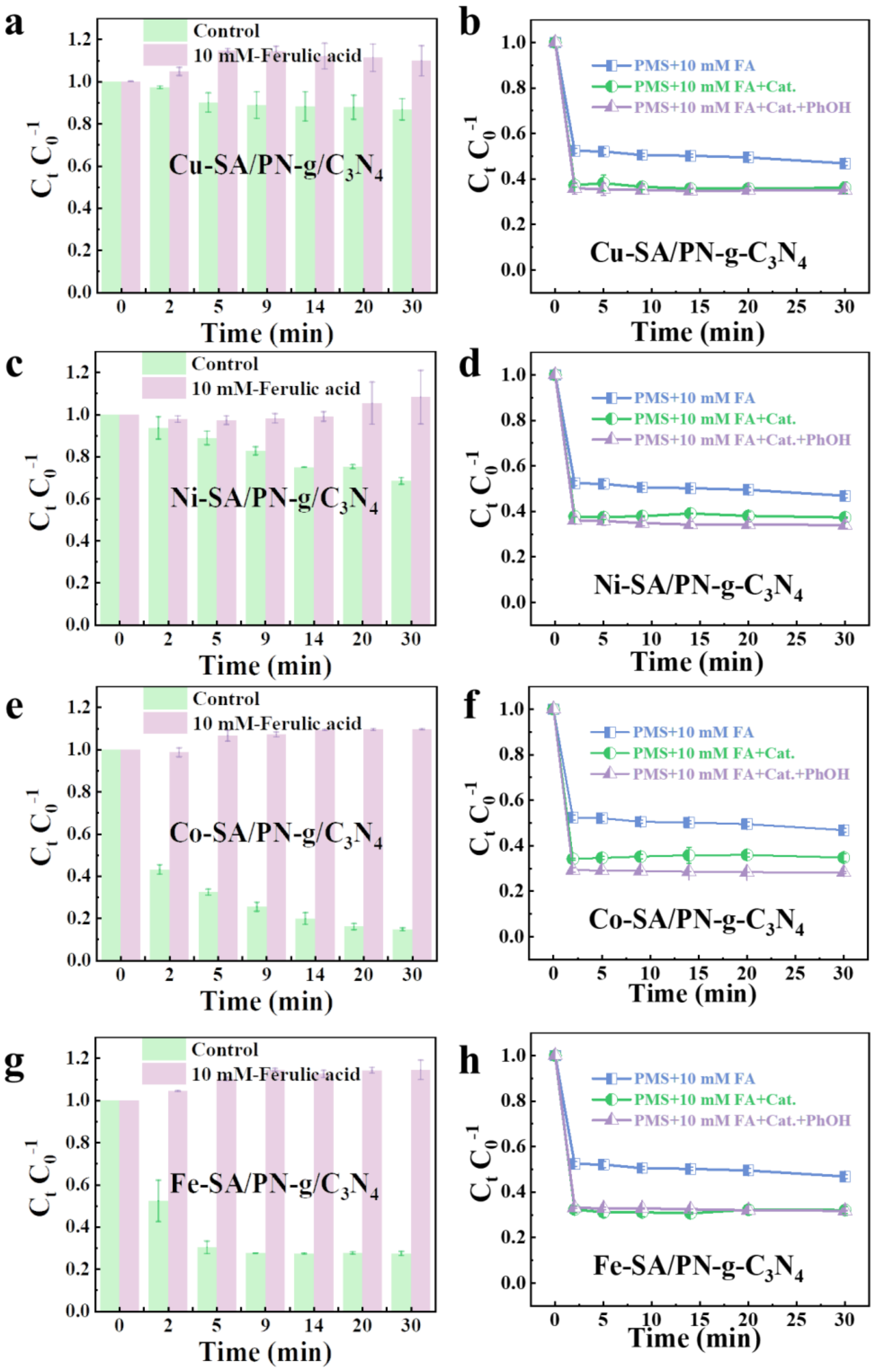

Supplementary Fig. 61 | FA is used as an inhibitor of phenoxyl radicals. The effect of FA on PhOH degradation (**a, c, e, and g**) and the corresponding PMS decomposition (**b, d, f, and h**) by adopting different TM-SA/PN-g/C_3N_4 catalysts. Reaction conditions: [Cat.] = 1.0 g L^{-1}, [PMS] = 1.0 mM, [PhOH] = 0.5 mM, [FA] = 10 mM, initial pH = 7.0, T = 25 ± 2 °C. Error bars represent the standard deviation, obtained by repeating the experiment two times.

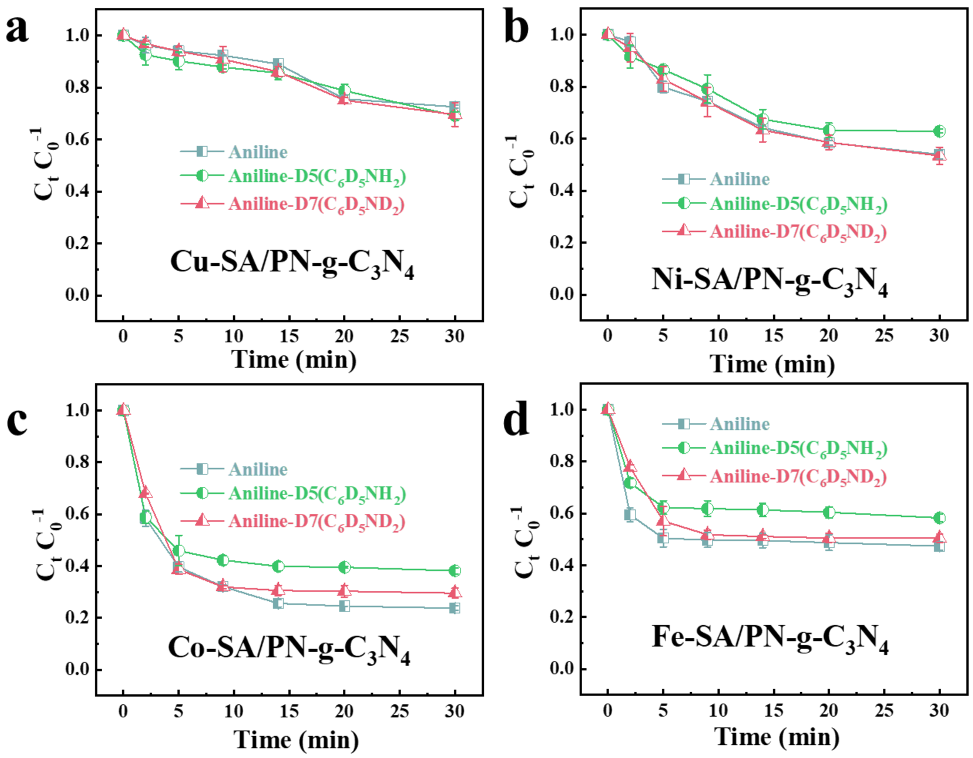

Supplementary Fig. 62 | Experiment on isotope substitution of aniline. a-d, H/D kinetic isotope effect for aniline oxidation through PMS activation by the Cu **(a)**, Ni **(b)**, Co **(c)**, and Fe **(d)**-SA/PN-g-C_3N_4 catalysts. Reaction conditions: [Cat.] = 1.0 g L^{-1}, [PMS] = 1.0 mM, [Aniline/Aniline-D5/Aniline-D7] = 0.5 mM, initial pH = 7.0, T = 25 ± 2 °C. Error bars represent the standard deviation, obtained by repeating the experiment two times.

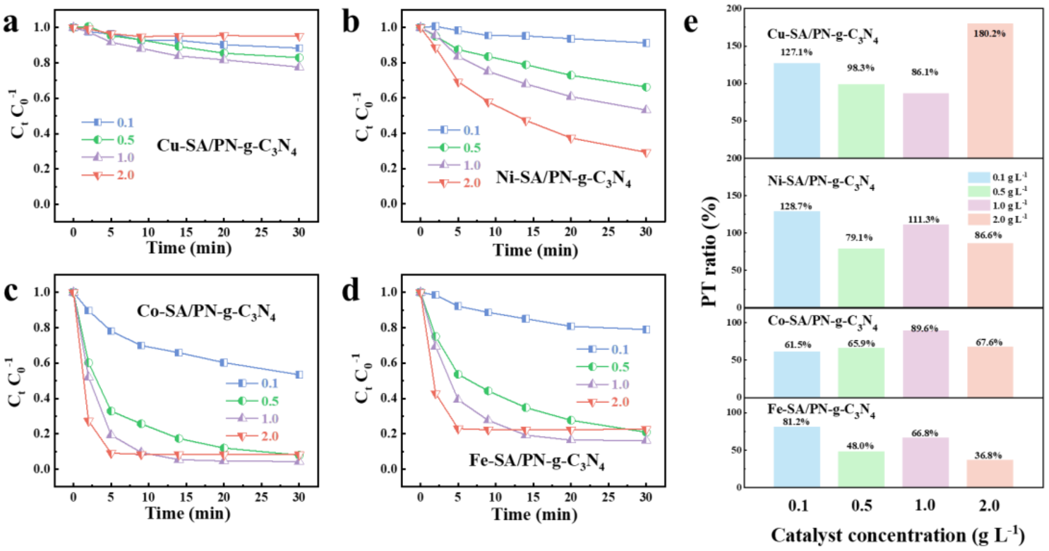

Supplementary Fig. 63 | Effect of catalyst concentration on PT ratio. a-d, The effect of catalyst dosage on PhOH degradation through PMS activation by the different TM-SA/PN-g-C_3N_4 catalysts. **e**, A summary of the corresponding PT ratio in the above reaction systems. Reaction conditions: [Cat.] =0.1, 0.5, 1.0, 2.0 g L^{-1}, [PMS] = 1.0 mM, [PhOH] = 0.5 mM, initial pH = 7.0, T = 25 ± 2 °C.

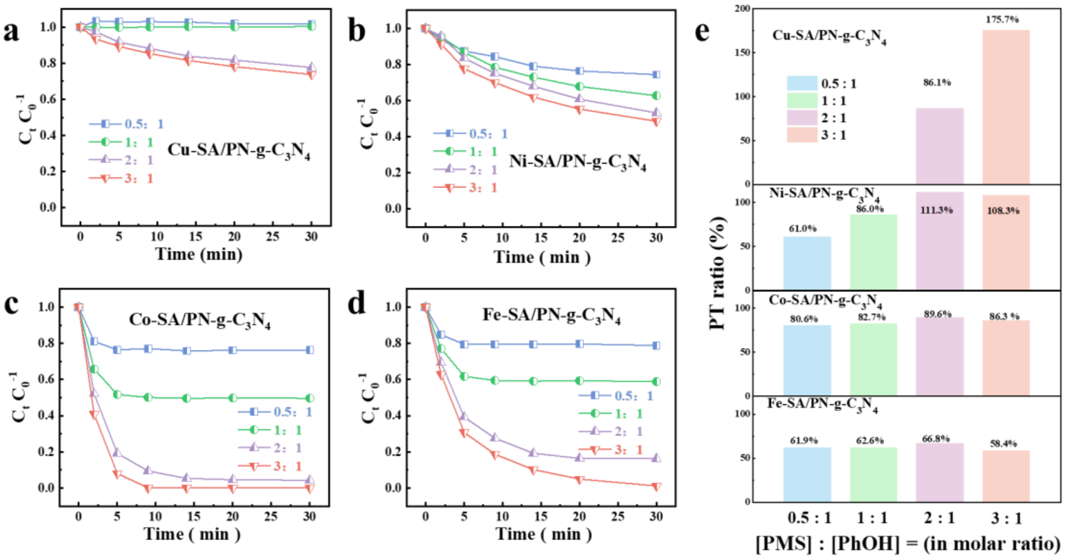

Supplementary Fig. 64 | Effect of oxidant and pollutant ratio on PT ratio. a-d, The effect of PMS concentration on PhOH degradation through PMS activation by the different TM-SA/PN-g-C_3N_4 catalysts. **e**, A summary of the corresponding PT ratio in the above reaction systems. Reaction conditions: [Cat.] = 1.0 g L^{-1}, [PMS] = 0.25 (0.5: 1), 0.5 (1: 1), 1.0 (2: 1), 1.5 (3: 1) mM, [PhOH] = 0.5 mM, initial pH = 7.0, T = 25 ± 2 °C.

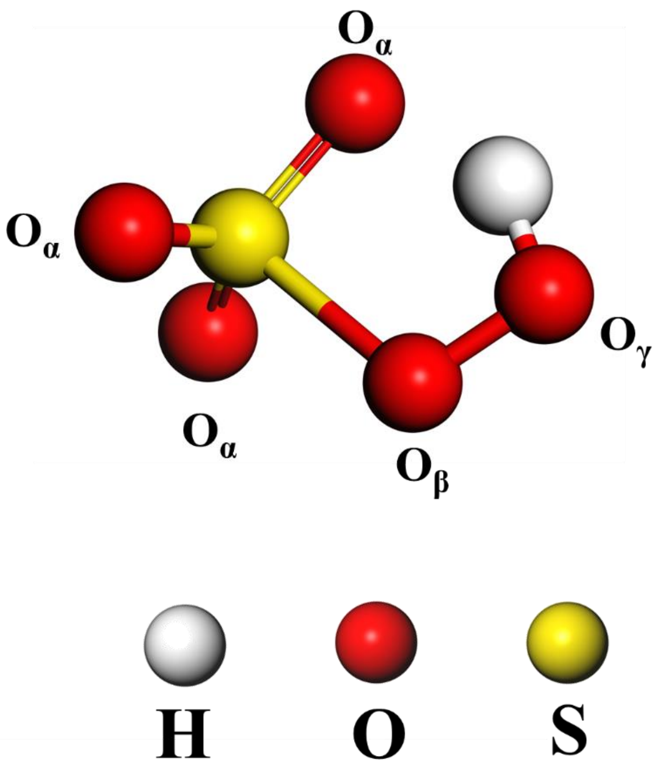

Supplementary Fig. 65 | A diagram for the three different categories of O atoms in PMS molecule. O_α: three terminal O atoms bonded to the S atom; O_β: one O atom bonded to both the S atom and another O atom; and O_γ: the O atom at the peroxide terminal, bonded to the H atom[8].

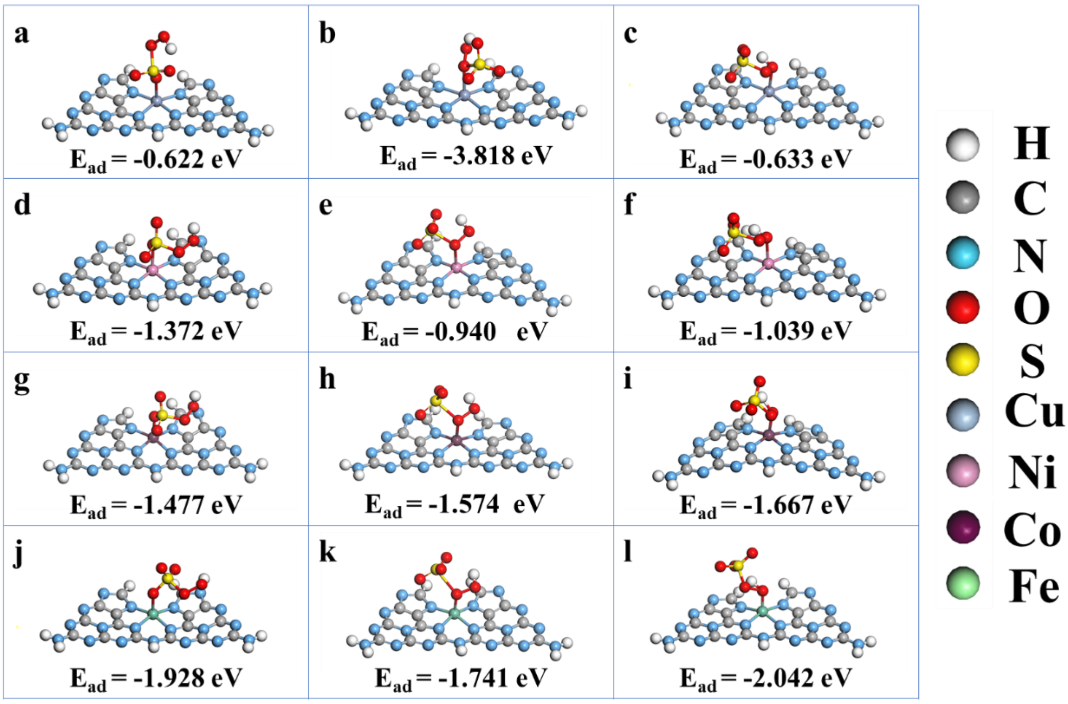

Supplementary Fig. 66 | Adsorption configuration of TM(TM=Cu, Ni, Co, Fe)-SA/PN-g-C_3N_4 on PMS. a-l, Computational models and the corresponding adsorption energies for PMS adsorption on the TM (Cu, Ni, Co, Fe)-N_4 sites by binding with different categories of O atom in PMS molecule. Cu-O_α **(a)**, Cu-O_β **(b)**, Cu-O_γ **(c)**, Ni-O_α **(d)**, Ni-O_β **(e)**, Ni-O_γ **(f)**, Co-O_α **(g)**, Co-O_β **(h)**, Co-O_γ **(i)**, Fe-O_α **(j)**, Fe-O_β **(k)**, and Fe-O_γ **(l)**.

Based on previous studies[8], we first defined the O species (O_α, O_β, and O_γ) on the PMS structure (Supplementary Fig. 65), and further evaluated the adsorption energy of different PMS oxygen adsorption configurations on TM(Cu, Ni, Co, Fe)-SA/PN-g-C_3N_4. Because the optimal adsorption configurations of the four metals are different (Cu-O_β, Ni-O_α, Co-O_γ, Fe-O_γ), to ensure the comparability of the calculation, O_γ is selected as the adsorption site, which is the medium adsorption configuration in Cu and Ni (Supplementary Fig. 65).

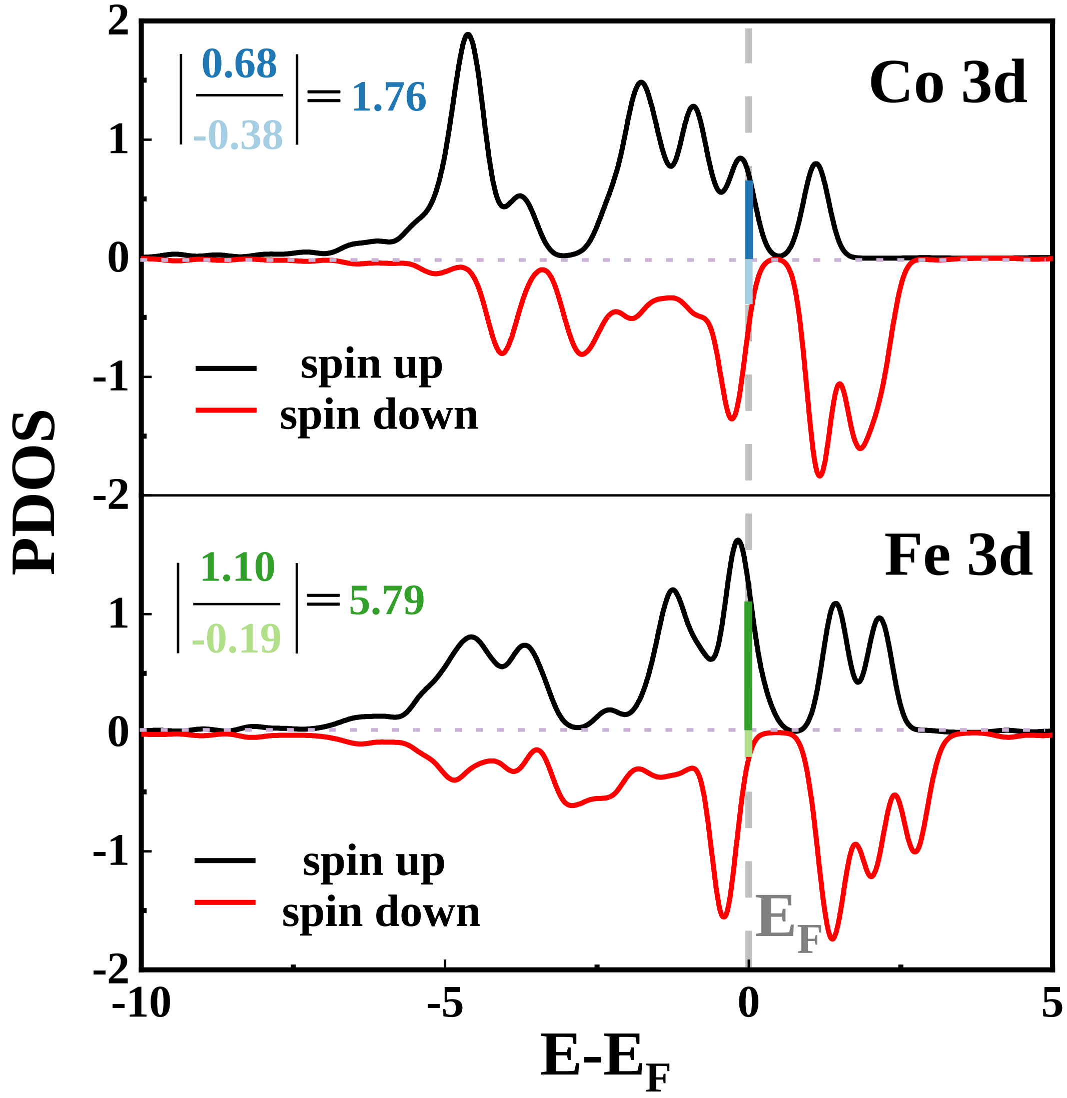

Supplementary Fig. 67 | The PDOS diagrams for Co(IV)=O and Fe(IV)=O.

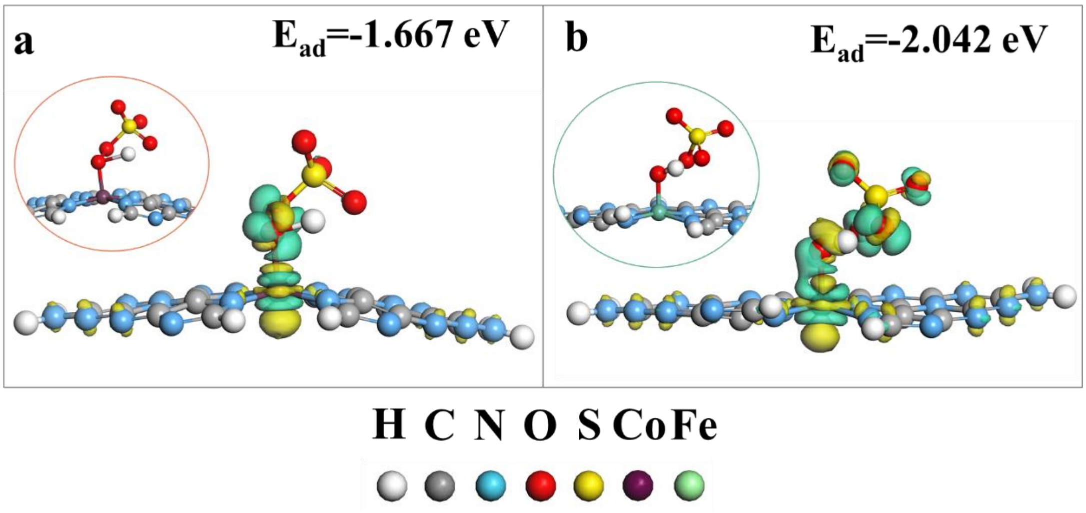

Supplementary Fig. 68 | Charge density difference diagram of PMS adsorption. a, b, Charge density differences and the corresponding adsorption energies for PMS activation on the Co-N_4 **(a)** and Fe-N_4 **(b)** sites.

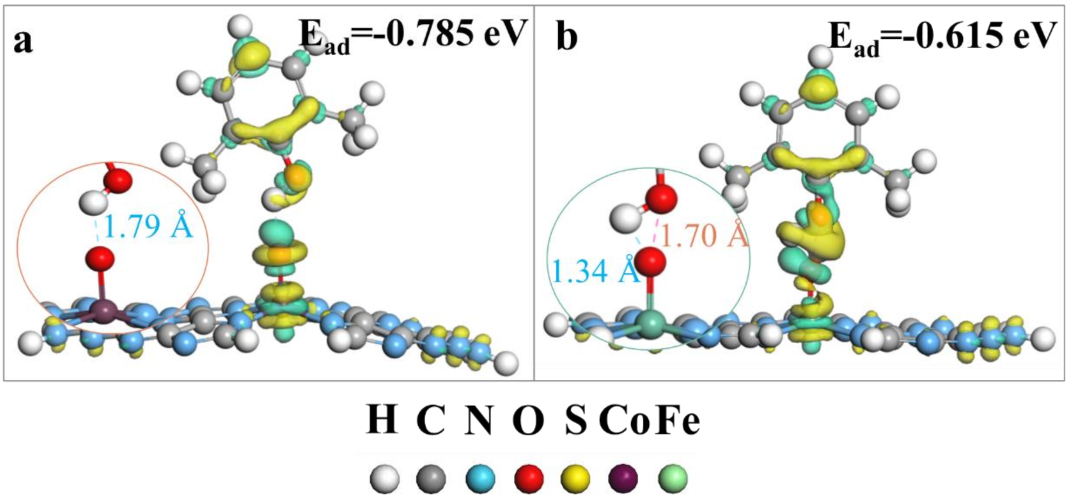

Supplementary Fig. 69 | Charge-density difference diagram of contaminant adsorbed by high-valent metal. Charge density difference and corresponding formation energy at the interface of (a) Co (IV)=O and (b) Fe (IV)=O after 2, 6-M-PhOH adsorption.

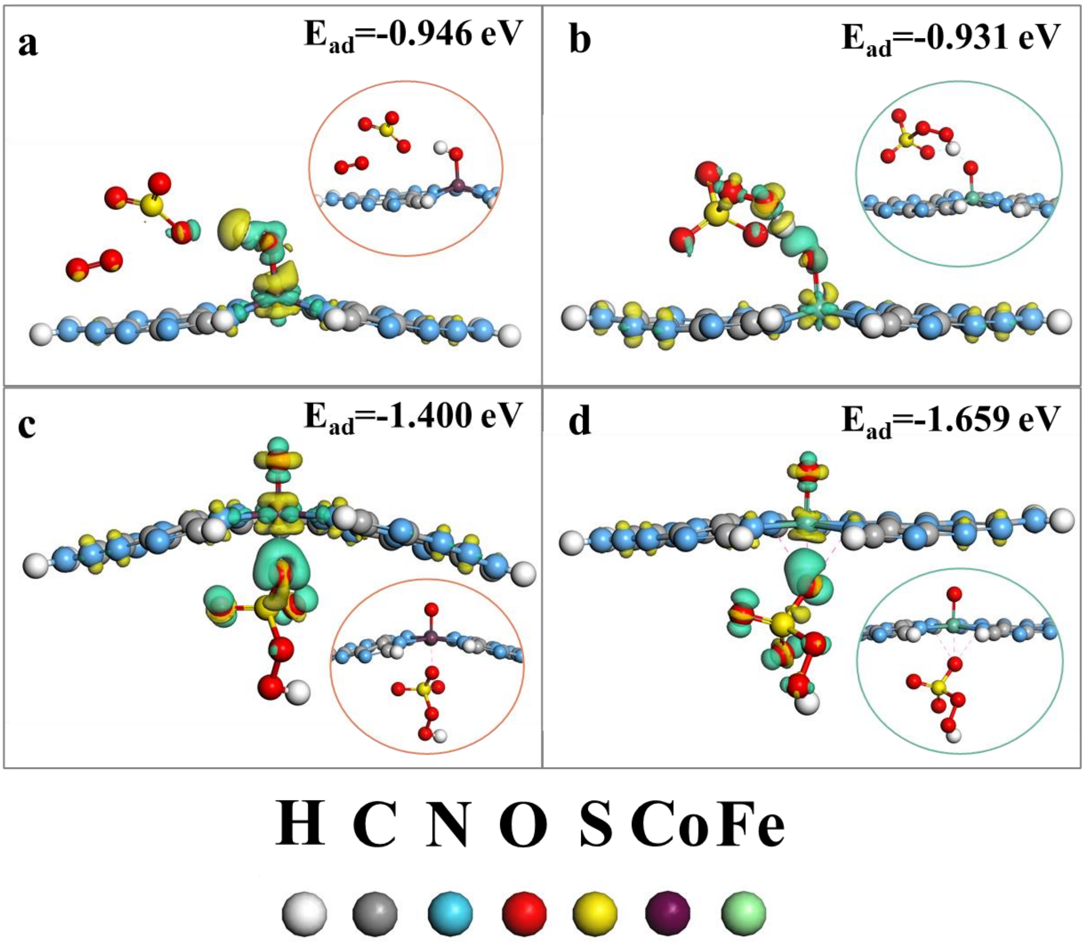

Supplementary Fig. 70 | Charge-density difference diagram of PMS adsorbed by high-valent metal. a-d, Charge density differences and the corresponding adsorption energies for PMS adsorption on the different adsorption sites (e.g., TM and O sites) of Co(IV)=O and Fe(IV)=O. Co(IV)=O-PMS* **(a)**, Fe(IV)=O-PMS* **(b)**, PMS*-Co(IV)=O **(c)** and PMS*-Fe(IV)=O **(d)**.

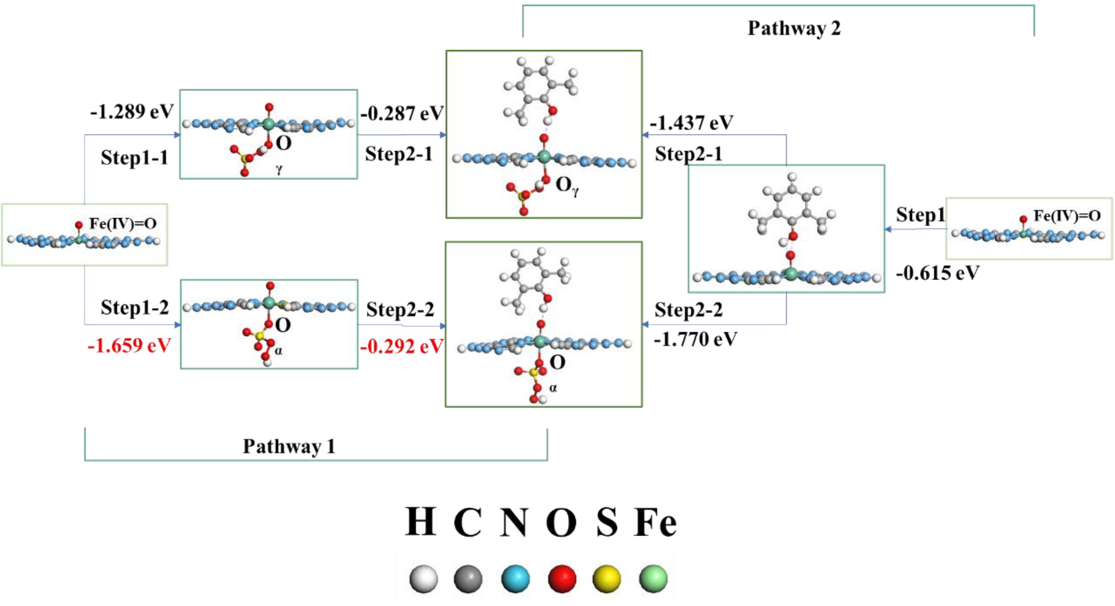

Supplementary Fig. 71 | Theoretical adsorption energy (ΔE_{ads}) of over-adsorption behavior that may occur on catalysts surface in Fe-SA/PN-g-C_3N_4.

According to the adsorption energies, Fe sites are prone to over-adsorption of PMS in the axial direction before and after adsorbing pollutants on the active sites. And the ability to over-adsorb PMS is three times that of pollutants.

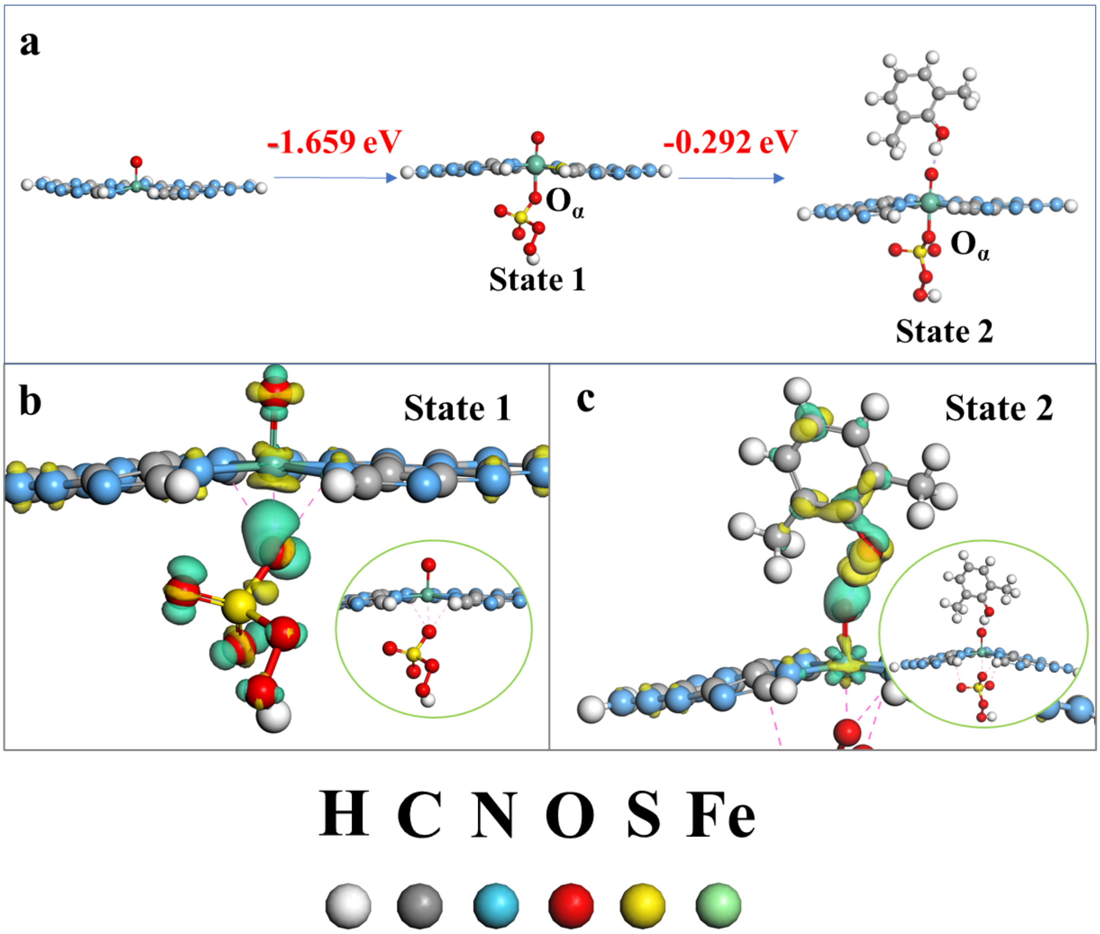

Supplementary Fig. 72 | Charge density difference and corresponding theoretical adsorption energy (ΔE_{ads}) of the most thermodynamically feasible over-adsorb path in Fe-SA/PN-g-C_3N_4.

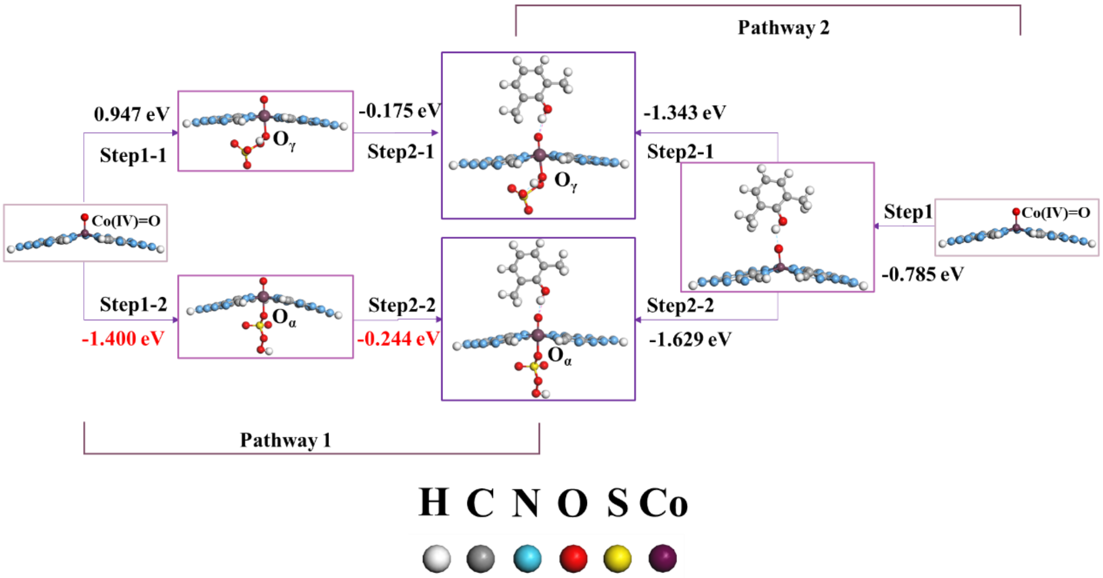

Supplementary Fig. 73 | Theoretical adsorption energy (ΔE_{ads}) of over-adsorption behavior that may occur on catalysts surface in Co-SA/PN-g-C_3N_4.

According to the adsorption energies, Co sites are prone to over-adsorption of PMS in the axial direction before and after adsorbing pollutants on the active sites. And the ability to over-adsorb PMS is two times that of pollutants. But it's less energy overall than the Fe system.

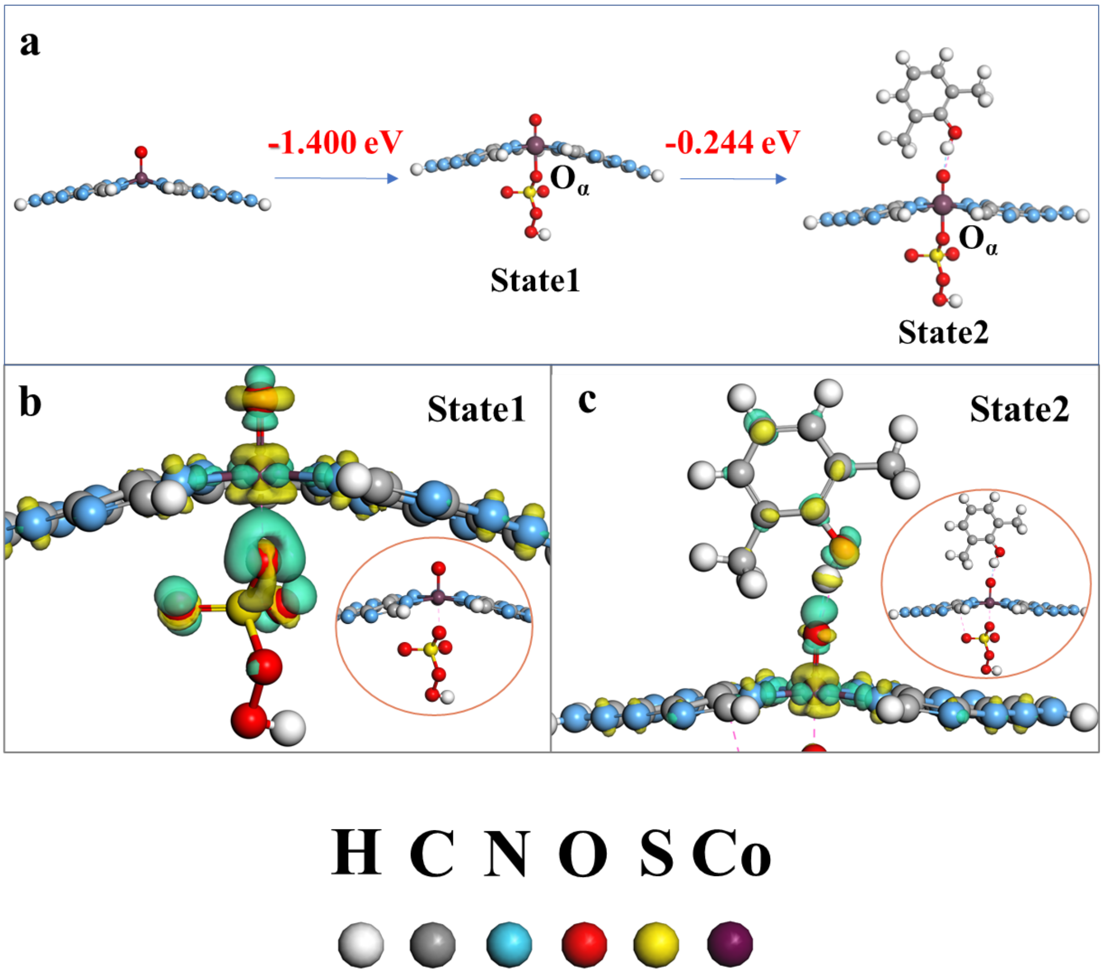

Supplementary Fig. 74 | Charge density difference and corresponding theoretical adsorption energy (ΔE_{ads}) of the most thermodynamically feasible over-adsorb path in Co-SA/PN-g-C_3N_4.

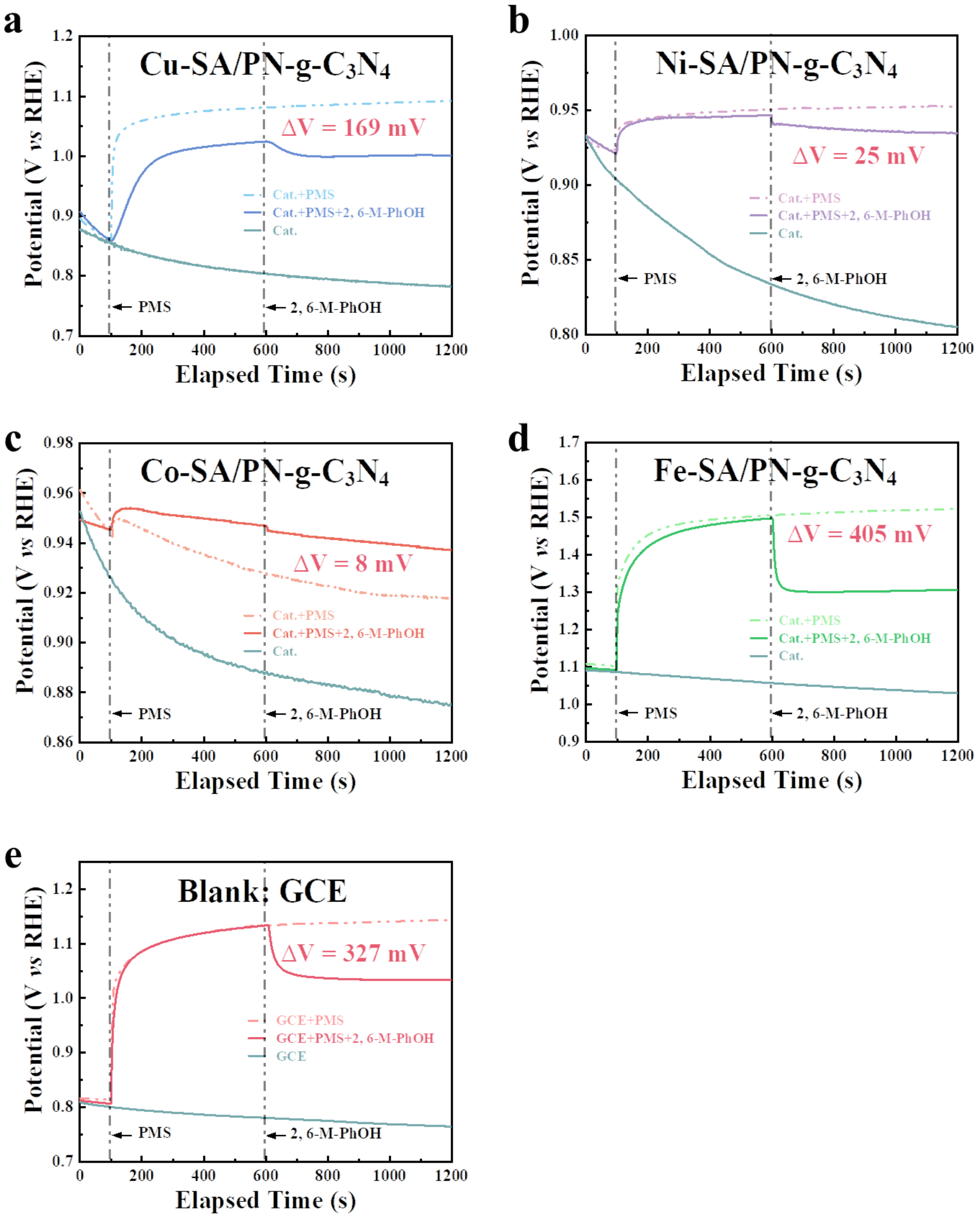

Supplementary Fig. 75 | The open circuit voltage measurements of the different TM (Cu, Ni, Co, Fe)-SA/PN-g-C_3N_4 catalysts in the presence of PMS and/or PhOH.

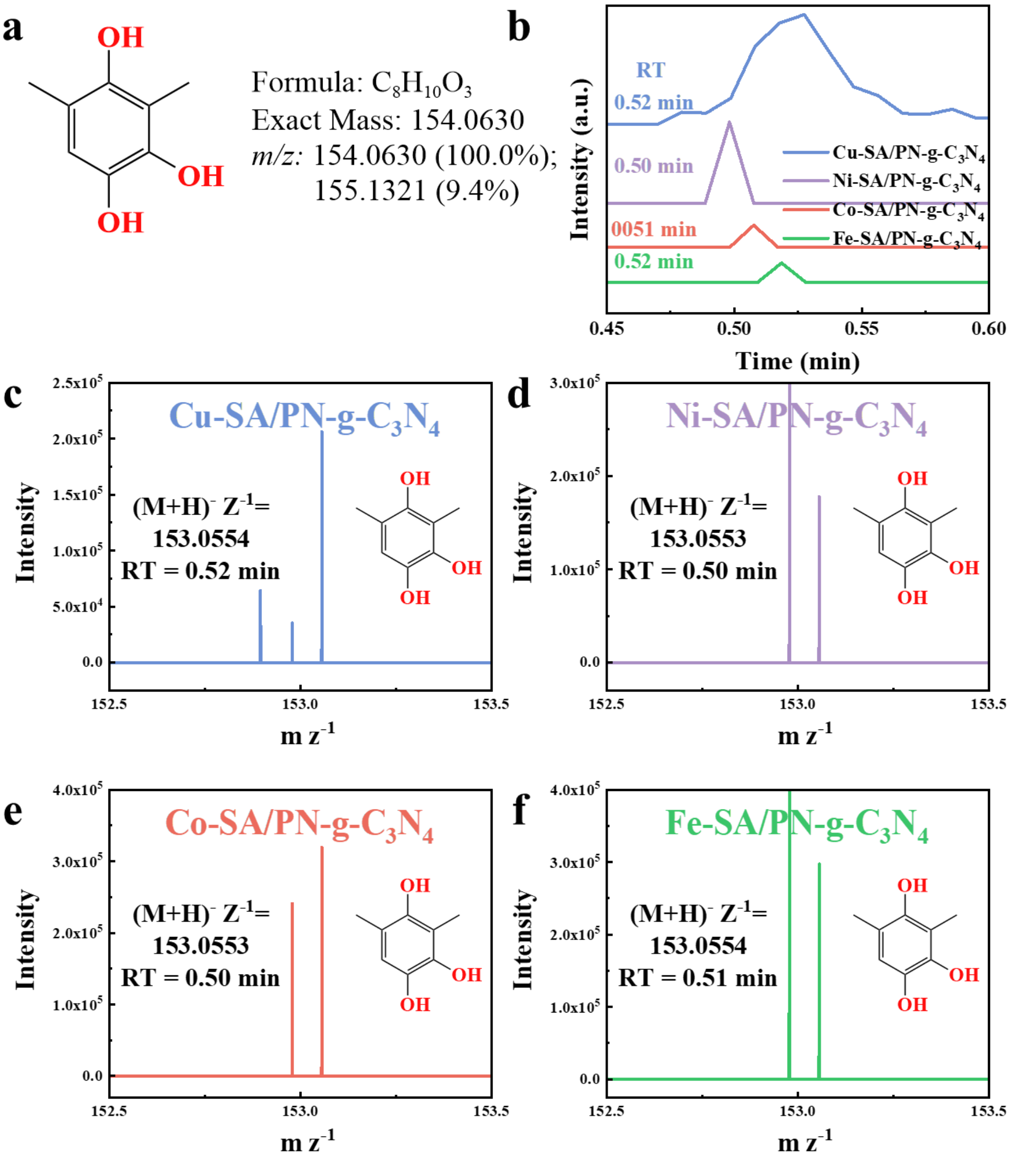

Supplementary Fig. 76 | The identification of the same degradation intermediates (1) in the different TM (Cu, Ni, Co, and Fe)-SA/PN-g-C_3N_4 systems. Reaction conditions: [Cat.] = 1.0 g L^{-1}, [PMS] = 1.0 mM, [2, 6-M-PhOH] = 0.5 mM, initial pH = 7.0, T = 25 ± 2 °C.

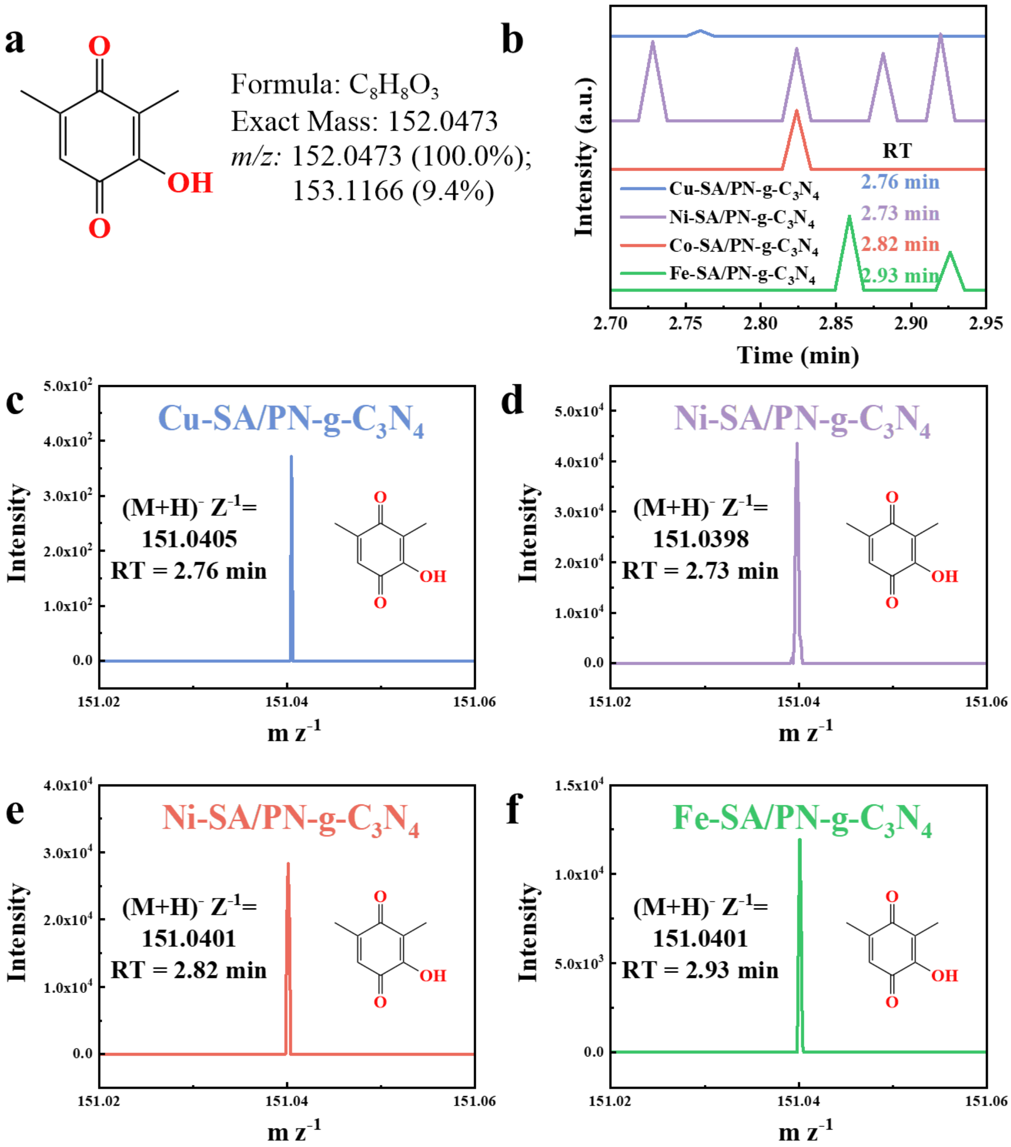

Supplementary Fig. 77 | The identification of the same degradation intermediates (2) in the different TM (Cu, Ni, Co, and Fe)-SA/PN-g-C_3N_4 systems. Reaction conditions: [Cat.] = 1.0 g L^{-1}, [PMS] = 1.0 mM, [2, 6-M-PhOH] = 0.5 mM, initial pH = 7.0, T = 25 ± 2 °C.

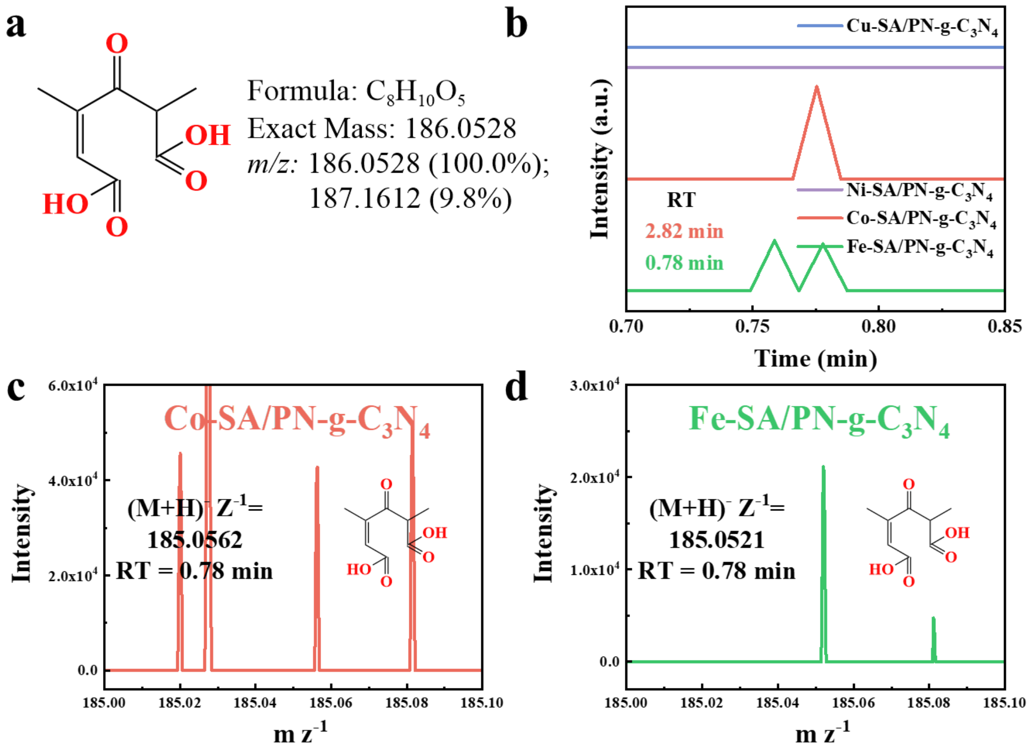

Supplementary Fig. 78 | The identification of the same degradation intermediates (1) in the different TM (Co, and Fe)-SA/PN-g-C_3N_4 systems. Reaction conditions: [Cat.] = 1.0 g L^{-1}, [PMS] = 1.0 mM, [2, 6-M-PhOH] = 0.5 mM, initial pH = 7.0, T = 25 ± 2 °C.

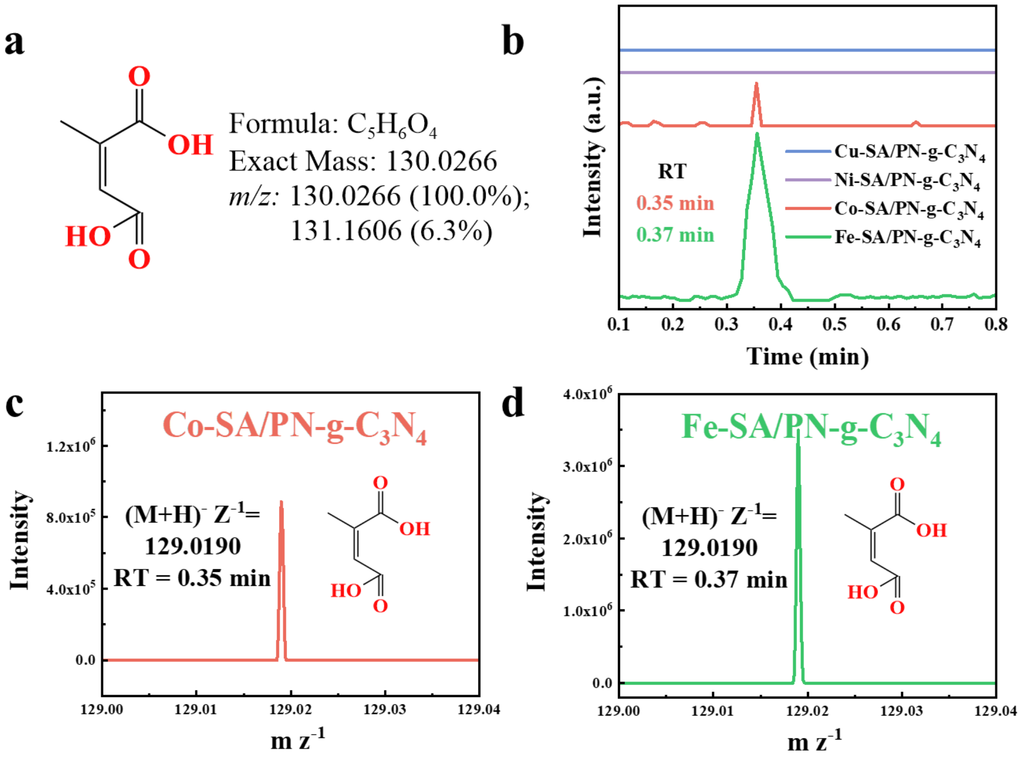

Supplementary Fig. 79 | The identification of the same degradation intermediates (2) in the different TM (Co, and Fe)-SA/PN-g-C_3N_4 systems. Reaction conditions: [Cat.] = 1.0 g L^{-1}, [PMS] = 1.0 mM, [2, 6-M-PhOH] = 0.5 mM, initial pH = 7.0, T = 25 ± 2 °C.

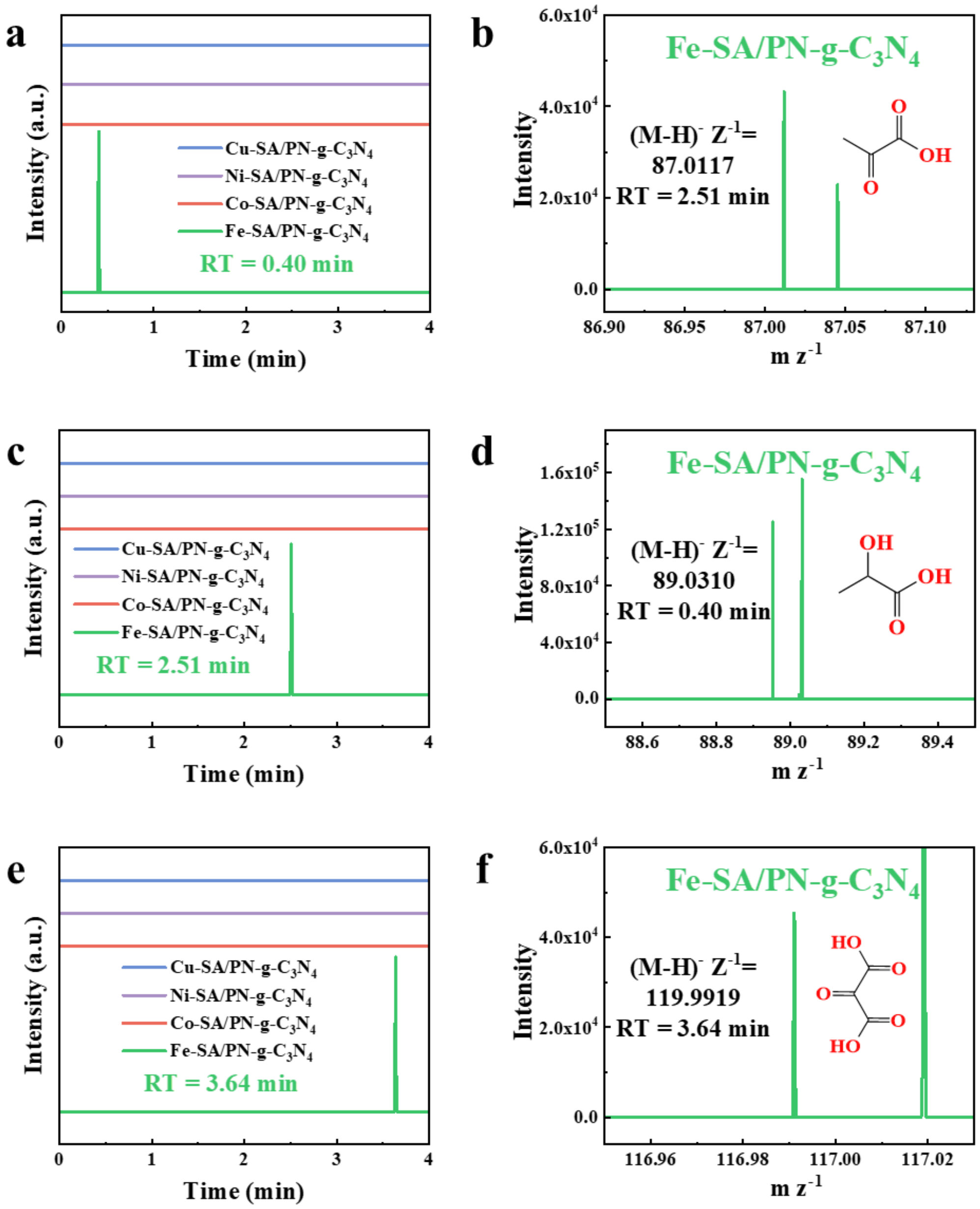

Supplementary Fig. 80 | The identification of different small organic acids in the Fe-SA/PN-g-C_3N_4/PMS system. Reaction conditions: [Cat.] = 1.0 g L^{-1}, [PMS] = 1.0 mM, [2, 6-M-PhOH] = 0.5 mM, initial pH = 7.0, T = 25 ± 2 °C.

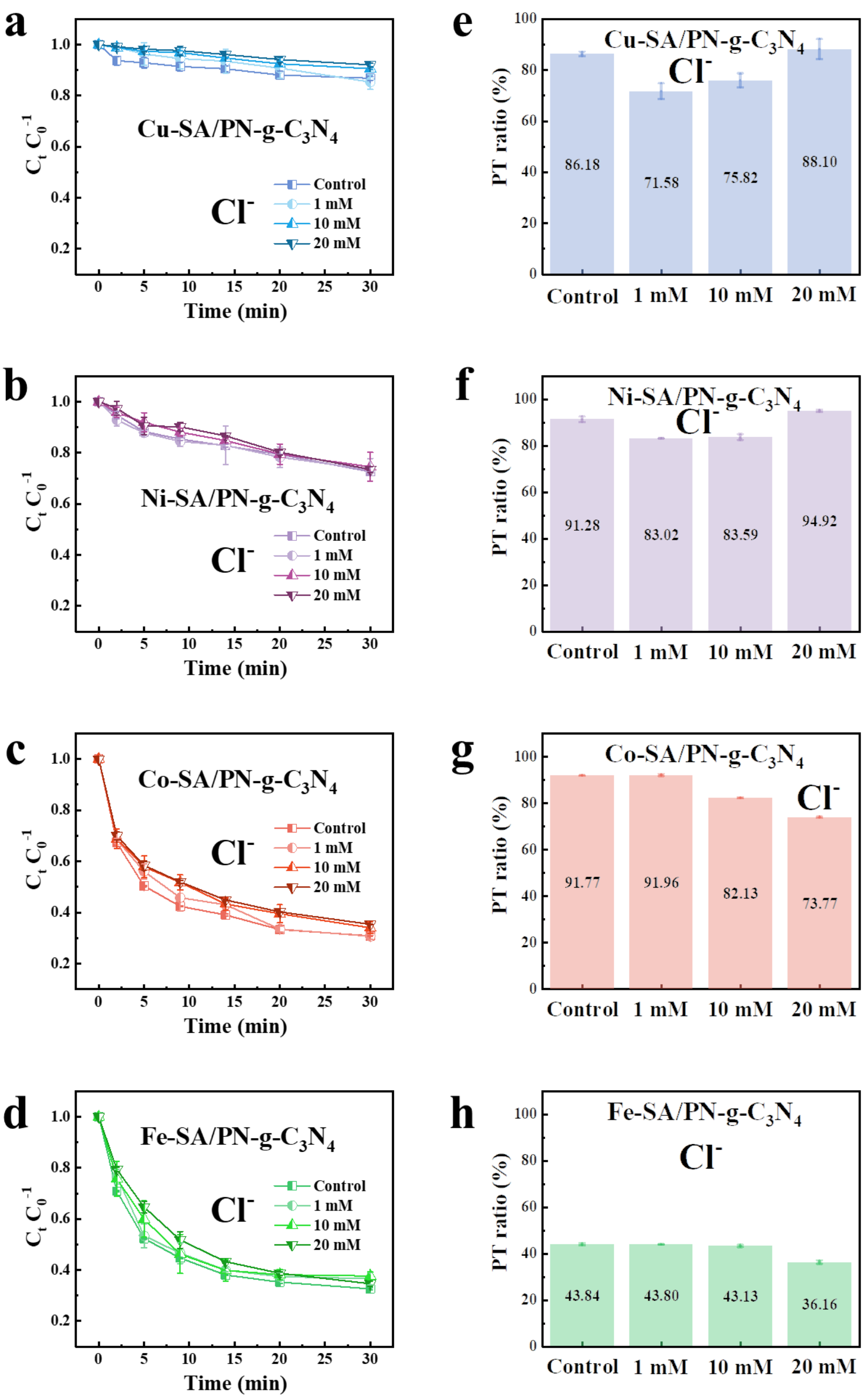

Supplementary Fig. 81 | Impact of Cl^- concentration on phenol degradation and PT ratios in TM-SA/PN-g-C_3N_4/PMS systems. a-d, The effect of Cl^- concentration on PhOH degradation in the four TM-SA/PN-g-C_3N_4/PMS systems. **e-h**, The corresponding PT ratios in the four reaction systems. Reaction conditions: [Cat.] = 1.0 g L^{-1}, [PMS] = 1.0 mM, [PhOH] = 0.5 mM, initial pH = 7.0, T = 25 ± 2 °C. Error bars represent the standard deviation, obtained by repeating the experiment two times.

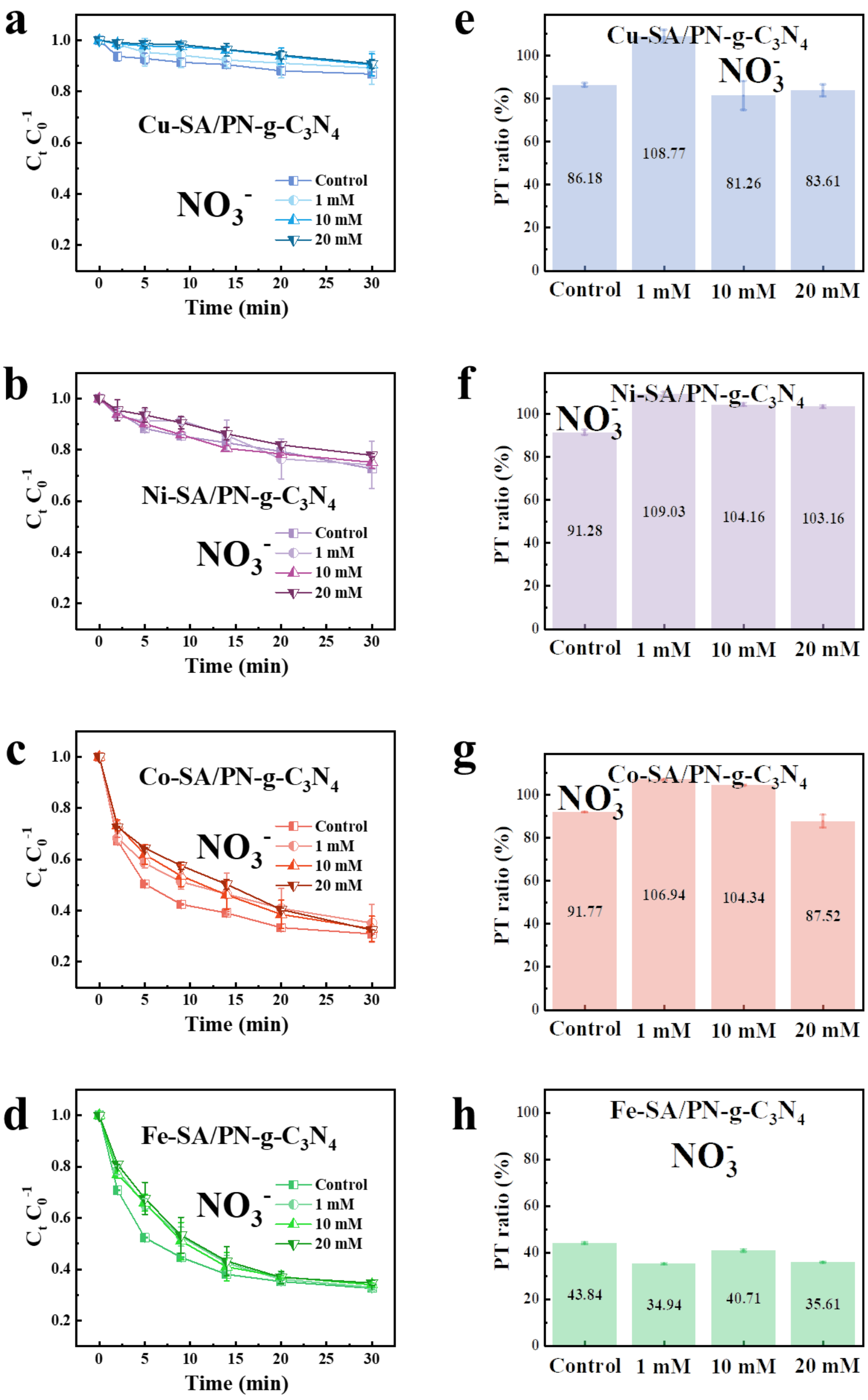

Supplementary Fig. 82 | Impact of NO_3^- concentration on phenol degradation and PT ratios in TM-SA/PN-g-C_3N_4/PMS systems. a-d, The effect of NO_3^- concentration on PhOH degradation in the four TM-SA/PN-g-C_3N_4/PMS systems. **e-h**, The corresponding PT ratios in the four reaction systems. Reaction conditions: [Cat.] = 1.0 g L^{-1}, [PMS] = 1.0 mM, [PhOH] = 0.5 mM, initial pH = 7.0, T = 25 ± 2 °C. Error bars represent the standard deviation, obtained by repeating the experiment two times.

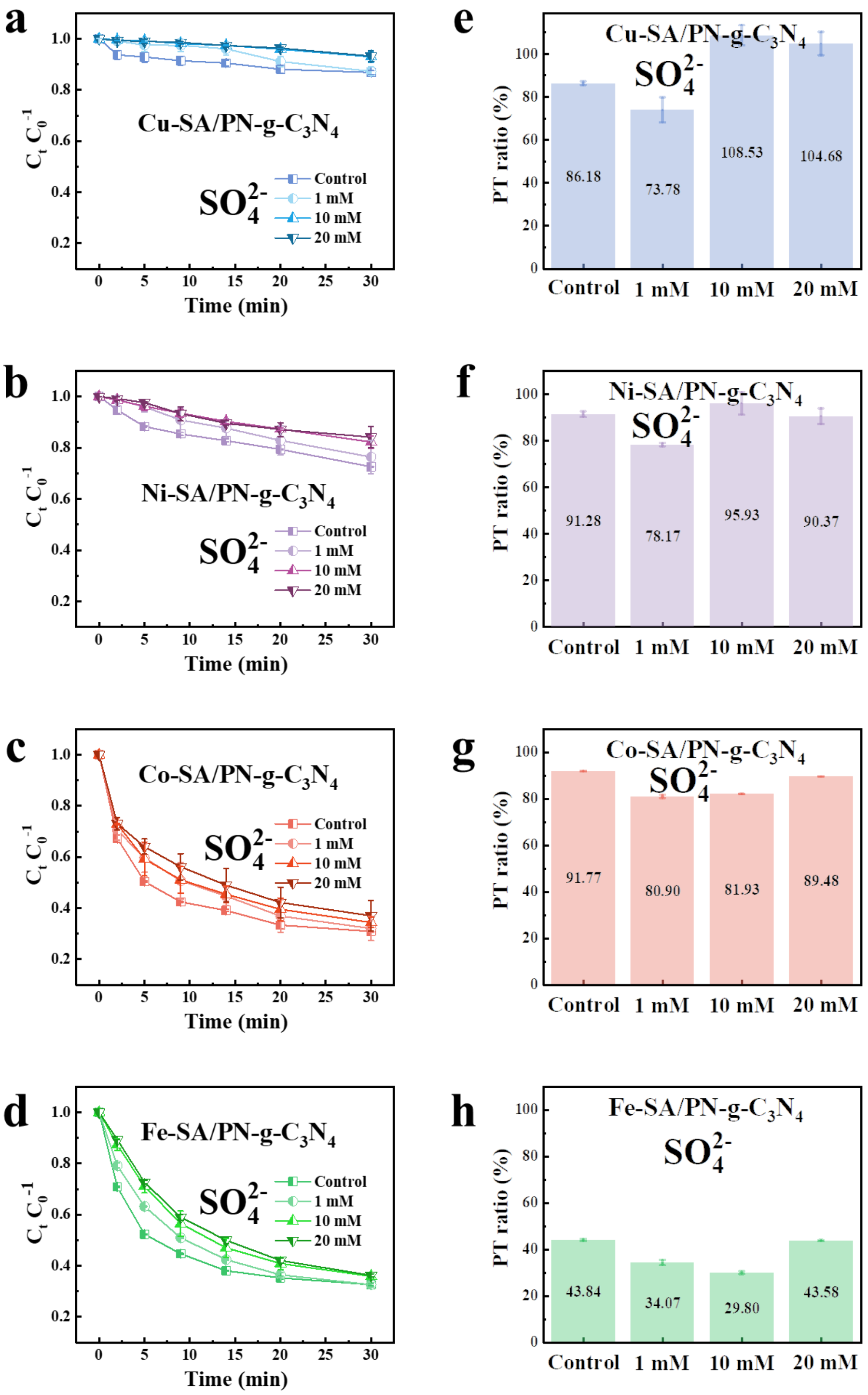

Supplementary Fig. 83 | Impact of SO_4^{2-} concentration on phenol degradation and PT ratios in TM-SA/PN-g-C_3N_4/PMS systems. a-d, The effect of SO_4^{2-} concentration on PhOH degradation in the four TM-SA/PN-g-C_3N_4/PMS systems. **e-h**, The corresponding PT ratio in the four reaction systems. Reaction conditions: [Cat.] = 1.0 g L^{-1}, [PMS] = 1.0 mM, [PhOH] = 0.5 mM, initial pH = 7.0, T = 25 ± 2 °C. Error bars represent the standard deviation, obtained by repeating the experiment two times.

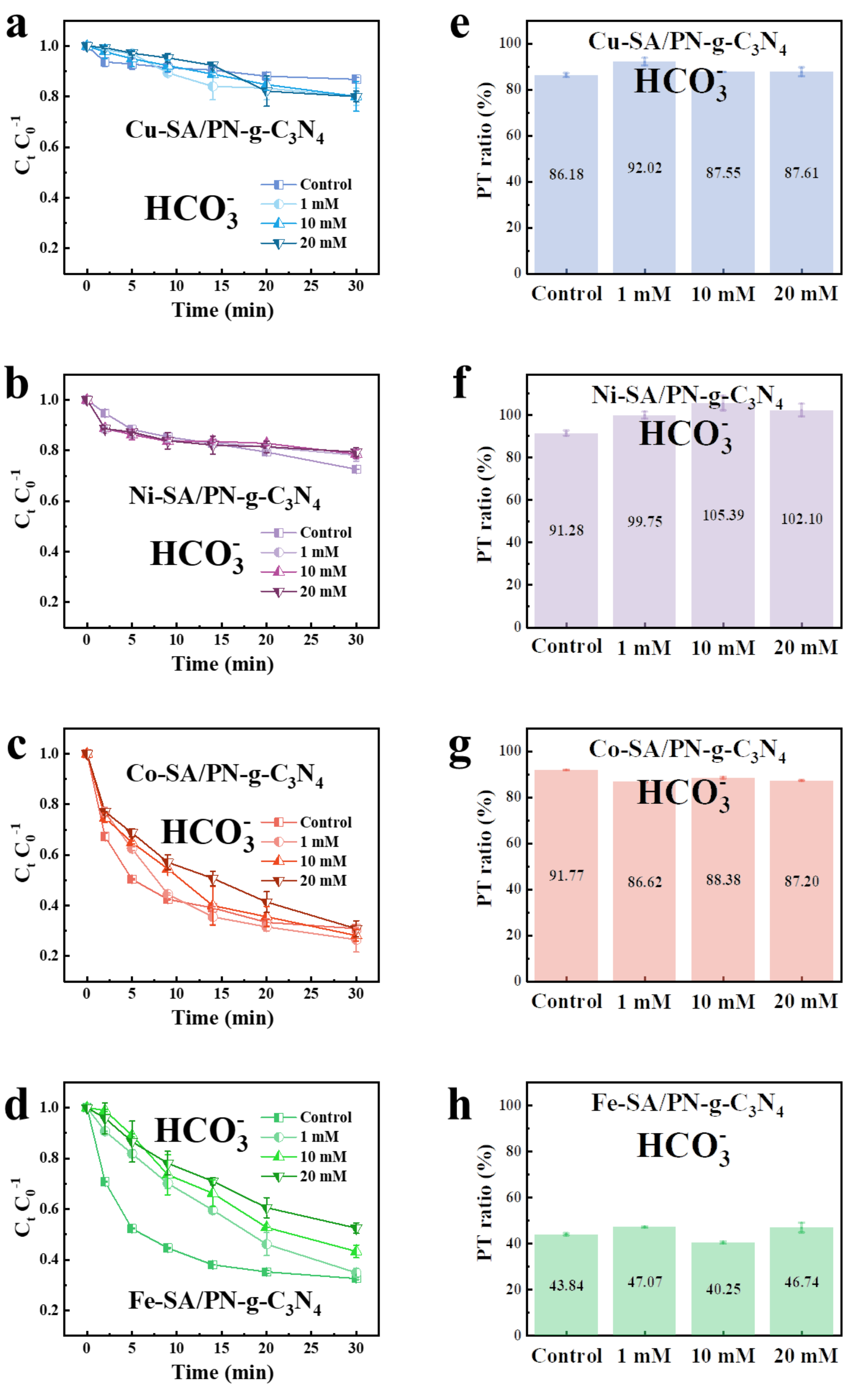

Supplementary Fig. 84 | Impact of HCO_3^- concentration on phenol degradation and PT ratios in TM-SA/PN-g-C_3N_4/PMS systems. a-d, The effect of HCO_3^- concentration on PhOH degradation in the four TM-SA/PN-g-C_3N_4/PMS systems. **e-h**, The corresponding PT ratio in the four reaction systems. Reaction conditions: [Cat.] = 1.0 g L^{-1}, [PMS] = 1.0 mM, [PhOH] = 0.5 mM, initial pH = 7.0, T = 25 ± 2 °C. Error bars represent the standard deviation, obtained by repeating the experiment two times.

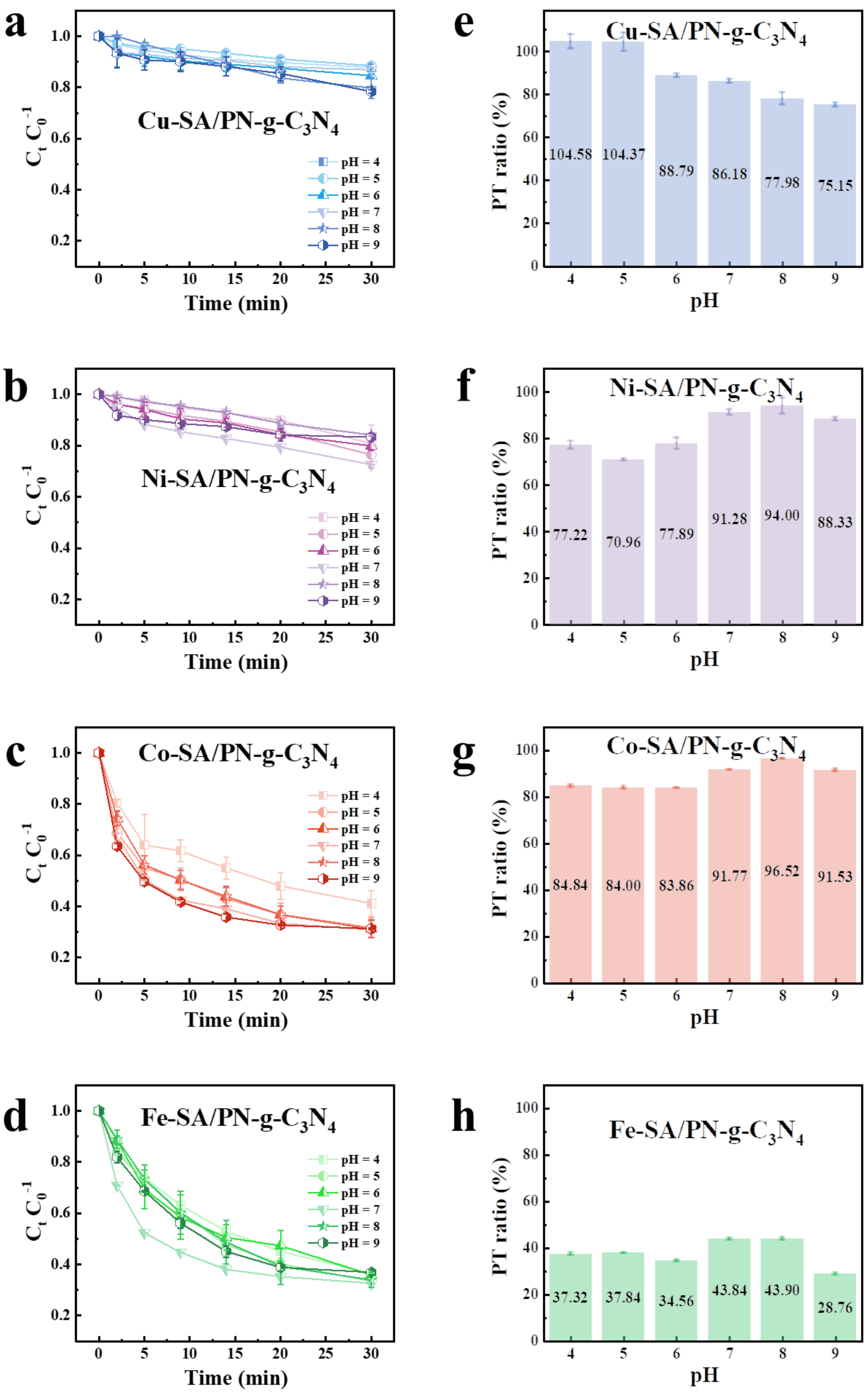

Supplementary Fig. 85 | Impact of pH on phenol degradation and PT ratios in TM-SA/PN-g-C_3N_4/PMS systems. a-d, The effect of pH on PhOH degradation in the four TM-SA/PN-g-C_3N_4/PMS systems. **e-h**, The corresponding PT ratio in the four reaction systems. Reaction conditions: [Cat.] = 1.0 g L-1, [PMS] = 1.0 mM, [PhOH] = 0.5 mM, initial pH = 7.0, T = 25 ± 2 °C. Error bars represent the standard deviation, obtained by repeating the experiment two times.

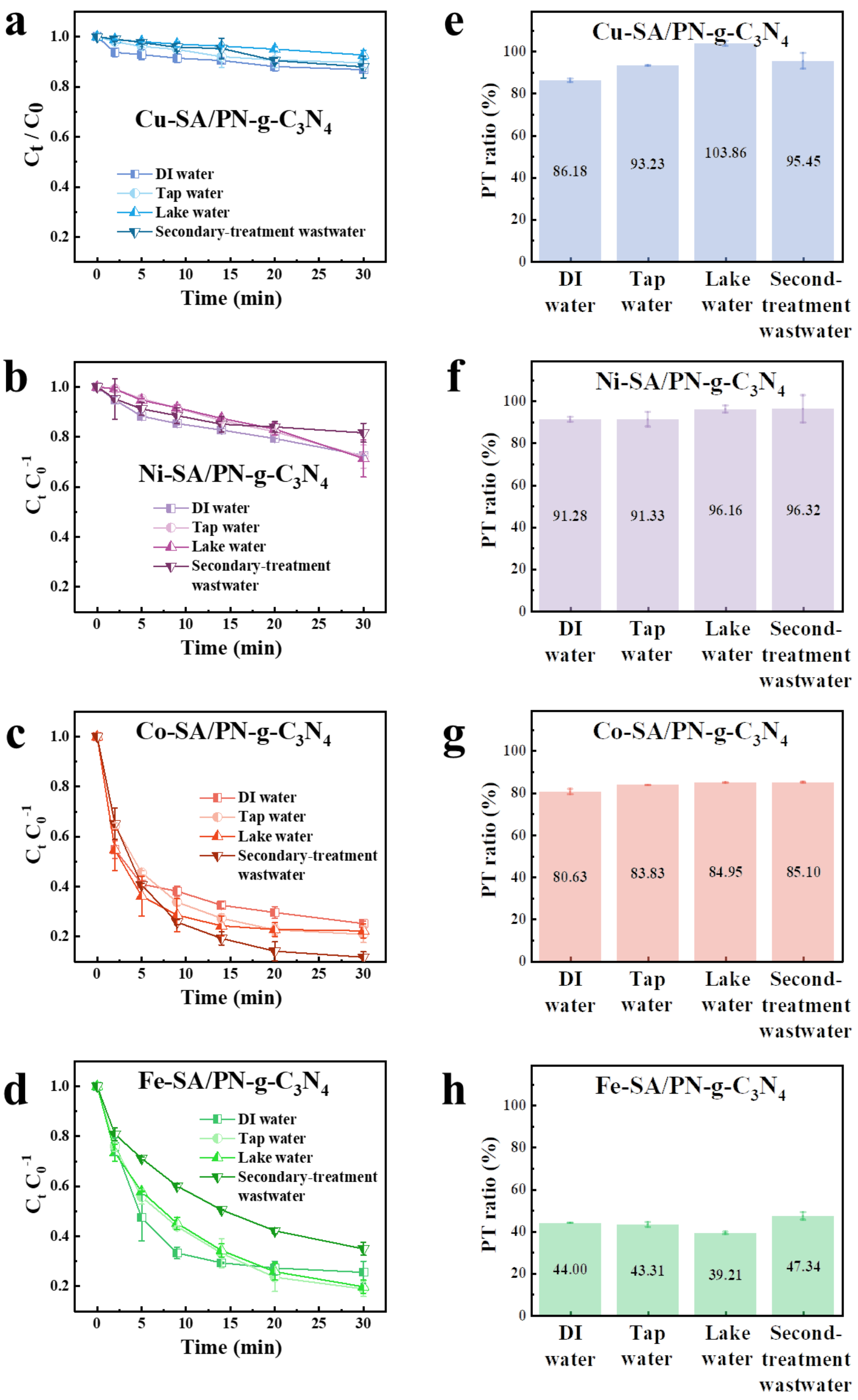

Supplementary Fig. 86 | Impact of water matrix on PhOH degradation and PT ratios in TM-SA/PN-g-C_3N_4/PMS systems. a-d, Effect of the water matrix on PhOH degradation in the four TM-SA/PN-g-C_3N_4/PMS systems. **e-h**, The corresponding PT ratios in the four reaction systems. Reaction conditions: [Cat.] = 1.0 g L^{-1}, [PMS] = 1.0 mM, [PhOH] = 0.5 mM, initial pH = 7.0, T = 25 ± 2 °C. Error bars represent the standard deviation, obtained by repeating the experiment two times.

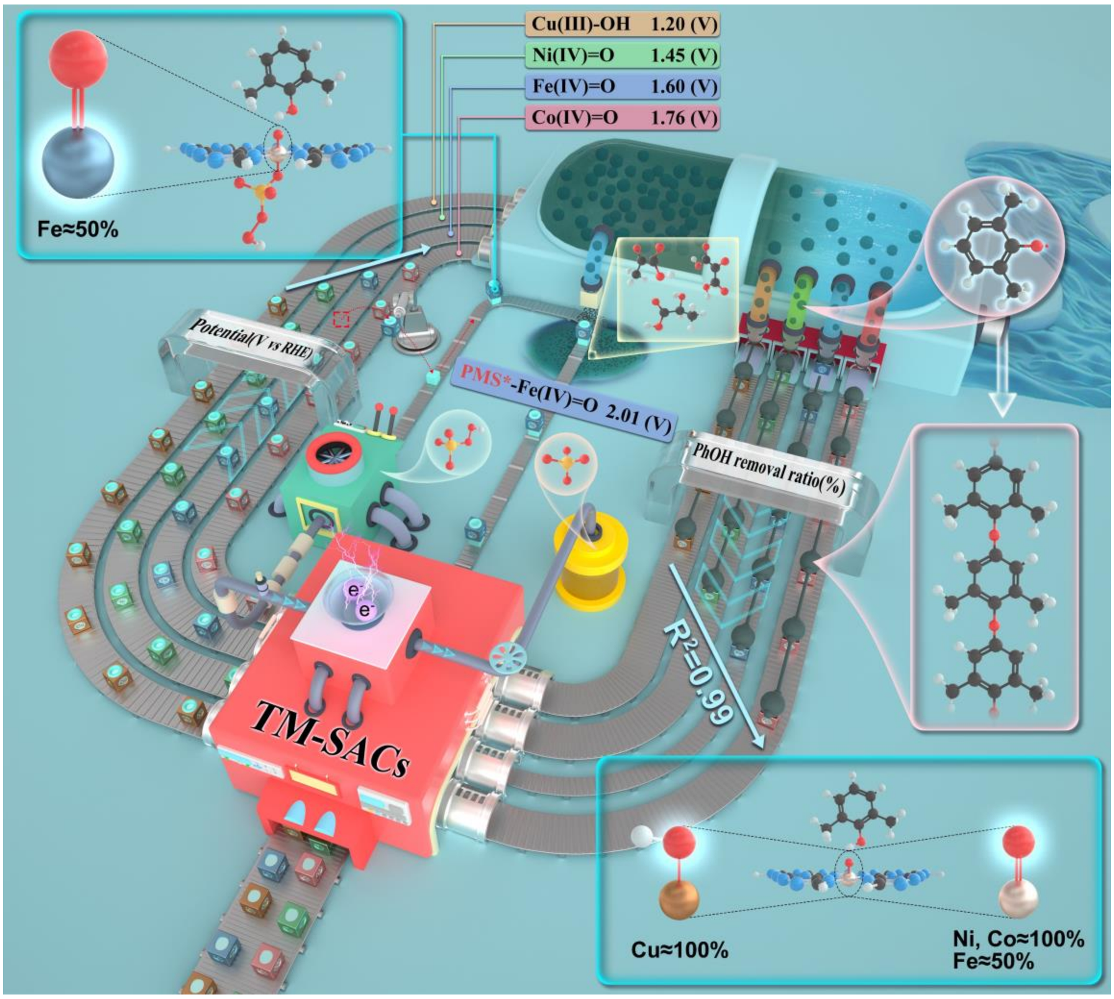

Supplementary Fig. 87 | Research summary diagram.

3 Supplementary Tables

Supplementary Table 1 | Summary of the electron-equivalent non-conservation data according to the literature

TM-SACs	Supporters	TOC removal efficiency	Oxidant: pollutant (in molar ratio)[c]	**PMS need**[d]		**PMS real**[e]	Ref
Cu	CN	72%	PMS: BPA=23.81:1	**25.92**	>	**23.81**	9
Co	MOFs	60.00%	PMS: CIP=29.76: 1	**34.20**	>	**29.76**	10
Fe	CNT	40.00%	PMS: BPA=20.00: 1	**14.40**	<	**20.00**	11

[a, b, c, e] These values were obtained from the corresponding literature.

[d] This value was calculated according to the amount of pollutant mineralization to CO_2 and H_2O. For the Fe-SACs/PMS/BPA system as an example, 1 mol BPA ($C_{15}H_{16}O_2$) → 15 mol CO_2 + 8 mol H_2O + 72 mol e^-; the e^- equivalence of the reductant was calculated from: [pollutant] × (TOC removal) × 72 = 1 × 40.00% × 72 = 28.80 mol. The amount of oxidizer required is [PMS] = 28.8 / 2 =14.40 mol.

In the degradation or mineralization process, the equivalence of the oxidant (i.e., the obtained electron equivalent of PMS) should be equal to or greater than that of the reductant (i.e., the given electron equivalent of pollutant). However, Supplementary Table 1 shows that the theoretical needed dosage of PMS in the Fe system for pollutant degradation is lower than the actual consumption amount of PMS, whereas opposite situations were observed in the Cu and Co systems. These results suggested that pollutants might undergo the degradation/mineralization pathway in the Fe-based catalytic system, but underwent the PT process in the Cu/Co-based catalytic systems.

Supplementary Table 2 | EXAFS fitting parameters at the Co, Ni, Cu, Fe K-edge for various samples

**Sample**	**Shell**	***CN*[a]**	***R*(Å)[b]**	**σ^2(Å^2)[c]**	**ΔE_0(eV)[d]**	***R* factor**
Cu foil	Cu-Cu	12*	2.54±0.01	0.008±0.0004	0.8±0.1	0.003
CuPc	Cu-N	4.1±0.7	1.94±0.01	0.001±0.0008	1.8±0.7	0.013
Cu sample	Cu-N	3.9±0.5	1.96±0.01	0.008±0.001	-7.9±1.7	0.014
Ni Foil	Ni-Ni	12*	2.48±0.01	0.005±0.0002	0.7±0.02	0.002
NiPc	Ni-N	3.9±0.8	1.88±0.01	0.002±0.002	-4.4±3.6	0.016
Ni sample	Ni-N	4.1±0.7	2.04±0.01	0.01±0.002	-6.4±1.8	0.02
Co foil	Co-Co	12*	2.49±0.01	0.0061±0.0004	-5.9±0.5	0.004
CoPc	Co-N	4.0±0.9	1.89±0.01	0.002±0.002	3.5±3.4	0.017
Co sample	Co-N	3.9±0.5	2.03±0.01	0.009±0.002	-3.2±1.5	0.016
Fe Foil	$Fe-Fe_1$	8*	2.46±0.01	0.004±0.002	5.3±2.9	0.006
	$Fe-Fe_2$	6*	2.84±0.01	0.005±0.003	5.3±4.3	
FePc	Fe-N	4.0±0.5	1.95±0.01	0.008±0.002	-1.6±2.3	0.009
Fe sample	Fe-N	3.9±1.2	1.89±0.01	0.01±0.004	-12.8±4.2	0.012

CN[a], coordination number; *R*[b], the distance between absorber and backscatter atoms; σ^{2c}, Debye-Waller factor to account for both thermal and structural disorders; $\Delta E_0{}^d$, inner potential correction; *R* factor indicates the goodness of the fit; S_0^2 is the amplitude reduction factor.

S_0^2 was fixed to 0.71 for Cu, 0.88 for Ni, 0.60 for Co, and 0.61 for Fe, according to the experimental EXAFS fit of Cu foil, Ni foil, Co foil, and Fe foil by fixing CN as the known crystallographic value.

Supplementary Table 3 | Theoretical adsorption and desorption energy of optimized configurations of PMS adsorbed on different TM (Co, Fe, Ni, Cu)-N_4 active sites.

**Configuration**	**$\Delta E_{adorption}$ (eV)- O_γ**	**$\Delta E_{desorption}$ (eV)**
Cu- N_4	-0.633	-1.839
Ni- N_4	-1.039	-1.516
Co- N_4	-1.667	-1.432
Fe- N_4	-2.042	-2.218

4 Supplementary Notes

4.1 Supplementary Note 1 | Identification of the TM (Cu, Ni, Fe)-N_4 site

Consistent with Co-SA/PN-g-C_3N_4, no metal clusters were observed in the other three catalysts (Cu, Ni, and Fe-SA/PN-g-C_3N_4), and the metals were uniformly dispersed in the form of single atoms according to various characterizations (Supplementary Figs. 1-8). The XANES spectra show that the valence states of the transition metals in TM-SA/PN-g-C_3N_4 were generally lower than those of their precursors (Cu: Cu^{+}~Cu^{2+}, Ni: Ni^{0}~Ni^{2+}, and Fe: Fe^{2+}~Fe^{3+}), which signifies notable metal-support interactions within the TM-SA/PN-g-C_3N_4 catalysts (Supplementary Fig. 7). In addition, the EXAFS fitting results indicate that the coordination numbers of the three TM-SA/PN-g-C_3N_4 catalysts were calculated to be approximately 4.0 (Supplementary Figs. 9-10, Supplementary Table 2). A similar TM-N_4 structure was verified for the three TM-SA/PN-g-C_3N_4 (TM = Cu, Ni, Fe) catalysts as that of Co-SA/PN-g-C_3N_4 based on the TOF-SIMS characterizations (Supplementary Fig. 11): Cu-N_4 (Cu-N_4^-: 118.03, Cu-N_4^+: 120.03), Ni-N_4 (Ni-N_4^-: 113.92, Ni-N_4^+: 115.03), and Fe-N_4 (Fe-N_4^-: 110.04, Fe-N_4^+: 112.94).

4.2 Supplementary Note 2 | Confirming the critical role of high-valent metals (Cu(III)-OH, Fe(IV)=O, and Ni(IV)=O) for pollutant removal

A combination of quenching and pre-oxidation experiments, EPR, Raman, XAS, and XPS characterizations were conducted to confirm the critical role of high-valent metals for pollutant removal.

(i) Excluding the contribution of free radicals:

Indeed, in the Co-SA/PN-g-C_3N_4/PMS system, MeOH and TBA had obvious inhibitory effects on the PhOH degradation rate, but they had negligible effects on the PhOH removal efficiency (approximately 5~10%) within 30 min. This result suggests that the primary mechanism of inhibition by these alcohols was likely due to competitive adsorption at the catalyst surface, which retarded the reaction kinetics,

rather than directly scavenging free radicals and thereby reducing the total amount of PhOH removed (Supplementary Fig. 16).

This interpretation is further substantiated by the disproportionately greater inhibitory effect of TBA compared to ethyl acetate (EA). In typical radical-based AOPs, EA, a scavenger of both $SO_4^{\bullet-}$ and $^{\bullet}OH$, is expected to have a more pronounced inhibitory effect than TBA, which primarily scavenges $^{\bullet}OH$[12]. However, the abnormally greater inhibitory effect of TBA in the Co-SA/PN-g-C_3N_4/PMS system implies that $SO_4^{\bullet-}$ and $^{\bullet}OH$ were not the major reactive species. Instead, this observation could be attributed to the fact that TBA has a greater surface affinity for Co-SA/PN-g-C_3N_4 due to its higher dielectric constant compared to MeOH (12.47 vs 33.0)[13]. In this regard, TBA, which has a higher surface affinity, could inhibit the surface polymerization reaction.

Although the EPR signal for $^{\bullet}OH$ was observed in filtrate of Cu-SA/PN-g-C3N4 system, but its intensity was not affected by the extra addition of pollutants and alcohols, indicating the absence of free radical mechanism. The emergency of $^{\bullet}OH$ might be attributed to secondary oxygen-active species from the conversion of high-valent metals (eq. S5, Supplementary Fig. 25).

$$TM^{(n+2)} + OH^{-} \rightarrow TM^{(n+1)} + \cdot OH \qquad TM = Cu,\ Ni,\ Co,\ Fe \tag{S5}$$

(ii) Excluding the contribution of $^1O_2$:

The $^1O_2$ signal was observed in the EPR spectrum, and it showed a significant decrease upon the addition of PhOH in the Co-SA/PN-g-C_3N_4 system (Supplementary Fig. 17). Nevertheless, the solvent exchange (H_2O to D_2O) did not enhance PhOH degradation, suggesting the negligible contribution of $^1O_2$ [14] (Supplementary Fig. 17).

(iii) Excluding the contribution of $O_2^{\bullet-}$:

To confirm the generation of $O_2^{\bullet-}$ in the four TM-SA/PN-g-C_3N_4/PMS systems, EPR trapping experiments in MeOH were conducted [5]. The results show that a negligible sextet peak for $O_2^{\bullet-}$ was detected in the four reaction systems (Supplementary Fig. 19), thus excluding the occurrence of $O_2^{\bullet-}$.

(iv) Excluding the contribution of surface-attached radicals:

Extra-added fluoride ions (F^-) with remarkable surface affinity can desorb surface radicals into aqueous solution[17]. However, the EPR signals for $SO_4^{\bullet -}$ and $^{\bullet}OH$ were not observed in the filtrate (Supplementary Fig. 20), suggesting that no surface-attached $SO_4^{\bullet -}$ or $^{\bullet}OH$ was present in the four TM-SA/PN-g-C_3N_4/PMS systems.

(v) Confirmation of the contribution of high-valent metals:

The quenching experimental results indicate that high-valent metals might be the key reactive species for organic pollutant degradation (Supplementary Fig. 16). The rationality of the such results were verified by the PMS decomposition experiments (Supplementary Fig. 21), which confirm that the catalytic activation of PMS by the different SACs in the presence of DMSO was still observed.

PMSO probing experiment provided solid evidence for the presence of high-valent mentals. The $TM_{(Ni, Co, Fe)}$ (IV)=O species can oxidize PMSO to methyl phenyl sulfone ($PMSO_2$) through the oxygen transfer pathway, while such a product is not formed through Cu(III)-OH oxidation, as Cu(III)-OH fails to form the stable metal-oxo species. The PMSO removal experiment shows that only 40% of the initial PMSO was transferred to $PMSO_2$ in the Cu-SACs/PMS system, while the transformation ratio reached 100% in the $TM_{(Ni, Co, Fe)}$-SACs/PMS systems (Supplementary Fig. 26), validating the generation of high-valent metal species via PMS activation. The presence of high-valent metals and their crucial role in pollutant degradation was further confirmed by the Raman characterizations. As shown in Supplementary Fig. 27, the addition of PMS resulted in the emergency of new peaks at 617, 618, and 664 cm^{-1} for Cu, Ni, and Fe-SA/PN-g-C_3N_4, respectively, indicating the generation of the corresponding high-valent metals. As expected, these Raman peaks disappeared upon the addition of pollutants.

We monitored the valence state of the single metal sites during a two-step reaction process using XPS and XAS characterizations (initially, the catalyst underwent PMS oxidation to form high-valent metals, which subsequently reacted with pollutants). With XPS a systematic analysis of the catalyst's valence state at three stages was conducted: before-reaction, pre-oxidation, and after-reaction. The results indicate a general trend of increasing and then decreasing valence states for metals. Notably, the characteristic

peak of Fe(IV)=O can be directly distinguished at the pre-oxidation stage in the XPS Fe 2*p* spectra[15] (Supplementary Fig. 30d). We then conducted XAS characterization of the catalysts at the pre-oxidation stage, which show direct evidence for the formation of the high-valent metal species (e.g., Cu(III), Ni(IV), Co(IV), and Fe(IV)) (Supplementary Fig. 28)[16-18]. Furthermore, we adopted a pre-oxidized catalyst to achieve direct degradation of pollutants, highlighting the crucial role of the high-valent metals. Although PN-g-C_3N_4 also exhibited a 10% degradation efficiency after pre-oxidation, the pre-oxidized TM (Cu, Ni, Co, and Fe)-SA/PN-g-C_3N_4 still exhibited a degradation efficiency of nearly 20% when excluding substrate influence (Supplementary Fig. 29).

The above results highlighted the critical role of high-valent metal species for pollutant degradation in the TM-SACs/PMS systems. These high-valent metal species can only oxidize the organics with electron-donating groups (e.g., PhOH and 2,6-M-PhOH), but failed to degrade the organic with an electron-withdrawing group (e.g, benzoic acid, BA) (Supplementary Fig. 31). Moreover, the high-valent metal species also only formed in the activation process of PMS instead of PDS, a widely used oxidant with the similar elementary composition of PMS. When using PDS as an oxidant in the SACs-catalytic systems, the pollutant could be hardly degraded due to the negligible activation of PDS (Supplementary Fig. 34), also, the Raman signals for the high-valent metal species were not observed (Supplementary Fig. 35).

4.3 Supplementary Note 3 | Polymerization removal of pollutants in TM-SA/PN-g-C_3N_4 catalytic PMS systems

(i) Validation of pollutant polymerization in different systems

The polymerization removal of pollutants in the Cu, Ni, and Fe-SA/PN-g-C_3N_4 catalytic PMS systems was verified via a series of experimental and characterization methods. First, TOC and pollutants were synchronously removed in the three reaction systems (Supplementary Figs. 32-33), which is a typical characteristic for the polymerization removal of the pollutant. Such results were confirmed by the SEM-EDS

and XPS analyses, which show a significantly increase in the content of C and O after the pollutant removal reaction, attributing to the benzene ring of pollutants and the C-O-C structure of surface polymers (Supplementary Figs. 38-46). TGA analyses demonstrate that the thermogravimetric losses were 3.26, 6.15 and 7.02% for the Cu, Ni, and Fe-based SACs, respectively (Supplementary Figs. 47-48), affirming the accumulation of organic matter on the catalyst surface. Furthermore, the surface accumulated polymers were eluted and identified to possess a PPO structure, with an *m/z* of 120.3, 120.2, and 120.3 for Cu, Ni, and Fe, respectively, as characterized by the MALDI-TOF-MS (Supplementary Fig. 49). The linked polyphenyl ether (PPO) structure of the surface polymers was further confirmed by a combination of NMR and FT-IR characterizations (Supplementary Figs. 50-51). The M_n values of the PPO products of Cu, Ni, and Fe-SA/PN-g-C_3N_4 were 4647, 4159, and 3345 Da, respectively (Supplementary Fig. 52).

(ii) Effect of polymers on the exposure of the reactive sites

To confirm whether the reactive sites of the catalysts were impacted by the polymer, cyclic tests were conducted on the TM (Co and Fe)-SA/PN-g-C_3N_4 catalysts. In the tests the stability and performance of these catalysts in repeated PhOH degradation cycles were assessed. The results reveal a decrease in the PhOH removal efficiency of 12.95% and 4.43% after 5 consecutive reaction cycles for Co-SA/PN-g-C_3N_4 and Fe-SA/PN-g-C_3N_4, respectively, validating the superior stability of the catalysts for cyclic PhOH degradation (Supplementary Fig. 53a-b). These results also suggest that the presence of polymers did not adversely affect the reactive sites on the catalyst surface, thus maintaining their catalytic efficiency in our experiments.

(iii) Catalyst regeneration

To regenerate the catalysts, we employed the two methods as depicted in Supplementary Fig. 53 c. In Method 1, tetrahydrofuran (THF) served as the solvent to dissolve and remove polymers accumulated on the catalyst surface. Method 2 involved the use of an atmospheric pyrolysis strategy, where the catalysts were subjected to a temperature of 350 °C for 30 min, effectively decomposing the surface-bound polymers. These two regeneration methods could completely recover the catalytic activities of the

catalysts to their original levels, as evidenced in Supplementary Fig. 53 a-b.

(iv) Collection of the surface-accumulated polymers

The surface-accumulated polymers could be facilely collected by an elution-drying protocol. The postreaction catalysts were collected and repeatedly washed with THF to obtain a yellow solution; the supernatant was then dried in an oven at 80 °C to obtain the solid polymers. The recovery ratios for these polymers were quantified as 81.57%, 81.30%, 88.43%, and 65.88% for Cu, Ni, Fe, and Co-SACs, respectively (Supplementary Fig. 54, and Methods 1.7).

4.4 Supplementary Note 4 | High-valent metals facilitate the oxidative polymerization of pollutants by generating phenoxyl radicals

The use of a CHANT trapping agent can simultaneously provide the results of UPLC-MS and EPR evidences for the presence of phenoxyl radicals. As shown in Supplementary Fig. 57, the reaction of a phenoxyl radical with CHANT yielded a stable, non-radical product and released persistent TEMPO radicals, which were detected by UPLC-MS with $(M+H)^+/Z$ values of 288.1928, 288.1928, 288.1929, and 288.1929 for the Cu, Ni, and Fe systems, respectively, and a mixture of the four samples. EPR results demonstrate the stability of the TEMPO signal produced by CHANT, which remains unaffected by various systems (Supplementary Fig. 58). After 24-h reaction, TEMPO signals were detected in all TM (Cu, Ni, Co, Fe) systems, and the presence of DMSO was found to inhibit the EPR signal.

TEMPO, as a traditional cross-coupling agent, was also used to capture phenoxyl radicals. The results show that phenoxyl radicals could cross-couple with TEMPO to form a stable adduct with $(M+H)^+/Z$ values of 278.2040, 278.2092, 278.2113, and 278.2037 for Cu, Ni, Fe-system, and a mixture of the four samples, respectively (Supplementary Fig. 59). Meanwhile, the addition of DMSO significantly increased the signal intensity of TEMPO (Supplementary Fig. 60), this could be attributed to that the high-valent metals was consumed by DMSO, leading to the decreased generation of phenoxyl radicals and lower consumption of TEMPO.

The role of phenoxyl radicals in governing the removal of pollutant was evaluated using FA as an inhibitor. When ca. 10 mM FA was added, the degradation reaction was immediately inhibited (Supplementary Fig. 61 a, c, g for Cu, Ni, and Fe-system), and the PMS could still be effectively activated by the catalyst rather than consumed by the inhibitor itself (Supplementary Fig. 61 b, d, f, h).

4.5 Supplementary Note 5 | A note on the PT ratio

The PT ratios for PhOH and 2,6-M-PhOH in the TM (Cu, Ni, Co, and Fe)-SA/PN-g-C_3N_4 catalytic PMS systems were determined, which depicted that the Fe-SACs/PMS system has the lowest PT ratios for the two organics (Supplementary Figs. 32, 33). Furthermore, the effects of catalyst dosage and PMS concentration on PT ratio for 2,6-M-PhOH were also studied. The results showed that the Fe-SACs/PMS system always maintained the specificity of the lowest PT ratio in the studied reaction systems. In addition, we also noted that the PT ratio was slightly affected by the reaction conditions in the four TM (Cu, Ni, Co, and Fe)-SA/PN-g-C_3N_4 catalytic PMS systems, indicating the universality of the PT process in the Fenton-like systems (Supplementary Figs. 63, 64).

4.6 Supplementary Note 6 | Revelation of the over-oxidation feature in the Fe-SACs catalytic system

As depicted in Supplementary Fig. 68, Fe exhibited significant charge transfer upon PMS adsorption and was highly susceptible to the formation of high-valent metal species. However, when it generated high-valent metal and then adsorbed pollutants, the adsorption energy was lower than that of Co, which is consistent with the kinetic trend, indicating that the degradation of 2, 6-M-PhOH was mainly caused by high-valent metal (Supplementary Fig. 69).

First, the generation of high-valent Fe was the primary mechanism. In addition to the experimental evidence provided by Raman, PMSO probe, pre-oxidation, XAS, XPS, etc., more importantly, DFT calculations reveal that the formation of Fe(IV)=O species

in the process, both the adsorption (Fig. 6(c)) and desorption (Supplementary Table 3) of PMS by the Fe-SA/PN-g-C_3N_4 were the easiest, indicating that the over-oxidation behavior occurred after the generation of high-valent metals.

Next, we determined the over-adsorption configuration of PMS, as shown in Supplementary Fig. 70, where PMS significantly favored the axial direction of Fe(IV)=O, forming the PMS*-Fe(IV)=O complex, rather than Fe(IV)= O-PMS *(-1.659 eV > -0.931 eV). As a control, the formation of PMS*-Co(IV)=O was much more difficult. In summary, the Fe in Fe(IV) = O more easily received electrons than O, which resulted in the over-adsorption of PMS, which further led to an increase in the oxidation ability of the system to over-oxidize pollutants to the small molecular acids, thus reducing the PT ratio.

Finally, we determined the degradation sequence of pollutants in the formation of over-oxidation behavior. The results show that Fe(IV)=O first combined with the O_α of PMS in the axial direction to form the PMS*-Fe(IV)=O complex, and then in the other axial direction, it bound with 2,6-M-PhOH (PMS*-Fe(IV)=O-2,6-M-PhOH), resulting in over-oxidation of pollutants. In contrast, the energy of this process was much lower in the Co system (Supplementary Fig. 71-74).

Supplementary References

1. Liang, C., Huang, C. F., Mohanty, N. & Kurakalva, R. M. A rapid spectrophotometric determination of persulfate anion in ISCO. *Chemosphere*. **73**, 1540-1543 (2008).
2. Clark, S. J. et al. First principles methods using CASTEP. *Z. Krist. Cryst. Mater.* **220**, 567-570 (2005).
3. Blochl, P. E., Jepsen, O. & Andersen, O. K. Improved tetrahedron method for brillouin-zone integrations. *Phys. Rev. B. Condens. Matter*. **49**, 16223-16233 (1994).
4. Grimme, S. Semiempirical GGA-type density functional constructed with a long-range dispersion correction. *J. Comput. Chem.* **27**, 1787-1799 (2006).
5. Chen, F. et al. Molecular engineering toward pyrrolic N-rich M-N_4 (M = Cr, Mn, Fe, Co, Cu) single-atom sites for enhanced heterogeneous fenton-like reaction. *Adv. Funct. Mater.* **31**, 2007877 (2021).
6. Jones, G. et al. First principles calculations and experimental insight into methane steam reforming over transition metal catalysts. *J. Catal.* **259**, 147-160 (2008).
7. Jang, D. et al. Water-assisted formation of amine-bridged carbon nitride: a structural insight into the photocatalytic performance for H_2 evolution under visible light. *Appl. Catal., B*. **310**, (2022).
8. Wu, X. & Kim, J. Outlook on single atom catalysts for persulfate-based advanced oxidation. *Acs Es&T Eng.* **2**, 1776-1796 (2022).
9. Pan, J. et al. Improving peroxymonosulfate activation by copper ion-saturated adsorbent-based single atom catalysts for the degradation of organic contaminants: electron-transfer mechanism and the key role of Cu single atoms. *J. Mater. Chem. A*. **9**, 1164-11613 (2021).
10. Mi, X. et al. Almost 100% peroxymonosulfate conversion to singlet oxygen on single-atom CoN_{2+2} sites. *Angew. Chem. Int. Ed. Engl.* **60**, 4588-4593 (2021).
11. Qian, K. et al. Single-atom Fe catalyst outperforms its homogeneous counterpart for activating peroxymonosulfate to achieve effective degradation of organic contaminants. *Environ. Sci. Technol.* **55**, 7034-7043 (2021).
12. Gu, C. et al. Slow-release synthesis of Cu single-atom catalysts with the optimized geometric

structure and density of state distribution for Fenton-like catalysis. *Proc. Natl. Acad. Sci. U. S. A.* **120**, (2023).

13. Huang, M. et al. In situ-formed phenoxyl radical on the CuO surface triggers efficient persulfate activation for phenol degradation. *Environ. Sci. Technol.* **55**, 15361-15370 (2021).
14. Shao, P. et al. Revisiting the graphitized nanodiamond-mediated activation of peroxymonosulfate: singlet oxygenation versus electron transfer. *Environ. Sci. Technol.* **55**, 16078-16087 (2021).
15. Hu, H., Zhang, Q., Wang, C., Chen, M. & Wang, Q. Facile synthesis of $CaMn_{1-x}Fe_xO_3$ to incorporate Fe(IV) at high ratio in perovskite structure for efficient in situ adsorption-oxidation of As(III). *Chem. Eng. J.* **435**, (2022).
16. Sun, H., Xu, X., Song, Y., Zhou, W. & Shao, Z. Designing high-valence metal sites for electrochemical water splitting. *Adv. Funct. Mater.* **31**, 2009779 (2021).
17. Zheng, X. et al. Theory-driven design of high-valence metal sites for water oxidation confirmed using in situ soft X-ray absorption. *Nat. Chem.* **10**, 149-154 (2018).
18. Zhang, N. et al. Lattice oxygen activation enabled by high-valence metal sites for enhanced water oxidation. *Nat. Commun.* **11**, 4066 (2020).
